# A computational analysis of *in vivo* VEGFR activation by multiple co-expressed ligands

**DOI:** 10.1371/journal.pcbi.1005445

**Published:** 2017-03-20

**Authors:** Lindsay E. Clegg, Feilim Mac Gabhann

**Affiliations:** 1 Institute for Computational Medicine, Institute for NanoBioTechnology, and Department of Biomedical Engineering, Johns Hopkins University, Baltimore, Maryland, United States of America; 2 Department of Materials Science and Engineering, Johns Hopkins University, Baltimore, Maryland, United States of America; Stanford University, UNITED STATES

## Abstract

The splice isoforms of vascular endothelial growth A (VEGF) each have different affinities for the extracellular matrix (ECM) and the coreceptor NRP1, which leads to distinct vascular phenotypes in model systems expressing only a single VEGF isoform. ECM-immobilized VEGF can bind to and activate VEGF receptor 2 (VEGFR2) directly, with a different pattern of site-specific phosphorylation than diffusible VEGF. To date, the way in which ECM binding alters the distribution of isoforms of VEGF and of the related placental growth factor (PlGF) in the body and resulting angiogenic signaling is not well-understood. Here, we extend our previous validated cell-level computational model of VEGFR2 ligation, intracellular trafficking, and site-specific phosphorylation, which captured differences in signaling by soluble and immobilized VEGF, to a multi-scale whole-body framework. This computational systems pharmacology model captures the ability of the ECM to regulate isoform-specific growth factor distribution distinctly for VEGF and PlGF, and to buffer free VEGF and PlGF levels in tissue. We show that binding of immobilized growth factor to VEGF receptors, both on endothelial cells and soluble VEGFR1, is likely important to signaling *in vivo*. Additionally, our model predicts that VEGF isoform-specific properties lead to distinct profiles of VEGFR1 and VEGFR2 binding and VEGFR2 site-specific phosphorylation *in vivo*, mediated by Neuropilin-1. These predicted signaling changes mirror those observed in murine systems expressing single VEGF isoforms. Simulations predict that, contrary to the ‘ligand-shifting hypothesis,’ VEGF and PlGF do not compete for receptor binding at physiological concentrations, though PlGF is predicted to slightly increase VEGFR2 phosphorylation when over-expressed by 10-fold. These results are critical to design of appropriate therapeutic strategies to control VEGF availability and signaling in regenerative medicine applications.

## Author summary

Angiogenesis, the growth of new blood vessels from the existing vasculature, is critical for maintenance of health and response to injury. In ischemic disease, this process is impaired, but therapies targeting a key family of proteins, the vascular endothelial growth factors (VEGF), have failed to translate clinically. This suggests a need for deeper understanding of the complex regulation underlying angiogenic signaling. Here, we translate a previously developed and validated model of VEGF family signaling into a human, whole-body framework. The different splice isoforms of VEGF and the related PlGF proteins have different affinities for the extracellular matrix (ECM) and the co-receptor Neuropilin-1. Using our model, we examine the effect of these different binding properties on the distribution of each isoform in tissue, and subsequent receptor signaling. The model predicts isoform-specific receptor activation that is consistent with observed vascular phenotypes in mice expressing a single VEGF isoform; non-ECM-binding isoforms lead to signaling that promotes cell proliferation, while strong ECM-binding promotes migratory signaling and increased vessel branching. This understanding is critical for design of biomaterials that manipulate VEGF-ECM binding to control growth factor delivery, and for understanding of splicing-induced changes in VEGF family signaling in different tissues and in disease.

## Introduction

Angiogenesis, the growth of new capillaries from the existing vasculature, is critical for maintenance of health and response to injury, as well as being a component of many diseases. However, regulation of angiogenesis is highly complex [1], and not fully understood. This complexity is a key reason for the lack of approved, effective therapies to promote angiogenesis for tissue engineering applications [2–4], for wound healing [5], or for ischemic diseases such as peripheral artery disease (PAD) [6], despite much research and multiple clinical trials [7]. Thus, a more complete, mechanistic understanding of the regulation of angiogenesis is crucial to designing more effective pro-angiogenic therapies.

Key to angiogenesis is the vascular endothelial growth factor (VEGF) family, including VEGF-A, VEGF-B, VEGF-C, VEGF-D, and placental growth factor (PlGF). VEGF-A (hereafter referred to as VEGF), considered the primary pro-angiogenesis VEGF ligand, has multiple splice isoforms, the most prevalent in humans being VEGF_121_, VEGF_165_, and VEGF_189_. Constitutive dimers of these splice isoforms bind to VEGF-receptor 1 (VEGFR1) and VEGF-receptor 2 (VEGFR2). Upon ligand binding, VEGF receptors dimerize, transphosphorylate, and initiate downstream signaling [8–10].

The longer two prevalent human VEGF isoforms (VEGF_165_ and VEGF_189_) contain heparin-binding domains, allowing for binding to heparan sulfate proteoglycans (HSPGs) in the extracellular matrix (ECM). These isoforms also have binding sites for the coreceptor Neuropilin-1 (NRP1), which regulates VEGF affinity for VEGFR2 and influences VEGFR2 trafficking, though the less-common heparin-binding VEGF_145_ does not bind to NRP1 [11, 12]. These isoform-specific properties have physiological significance; upon secretion into the extracellular space, VEGF_121_, which does not bind to the ECM or to NRP1, forms shallow gradients and diffuses away from the source of production, while VEGF_189_, which binds strongly to the ECM and also binds NRP1, forms steep interstitial gradients and remains close to the site of production [13].

In addition, mice and tumors expressing single VEGF isoforms have distinct phenotypes. Expression of only VEGF_121_ leads to formation of high diameter vessels with low branching density, while expression of only VEGF_189_ results in highly branched networks of very thin vessels. By contrast, expression of VEGF_165_ alone results in a phenotypically normal vasculature, with balanced branching and diameters [14–17]. In addition to regulating VEGF distribution, it has recently been shown that the immobilization of VEGF to ECM proteins or to a surface alters the site-specific phosphorylation profile of VEGFR2 *in vitro*. While phosphorylation of tyrosine Y1175, which leads to ERK1/2 activation and cell proliferation, is similar whether VEGF is immobilized or presented in solution, phosphorylation of Y1214, which leads to phosphorylation of p38 and cell migration, increases when VEGF is immobilized [18, 19]. This shift in signaling, which parallels the phenotypes seen with single VEGF isoform expression, can be explained by reduced internalization of VEGFR2 bound to immobilized VEGF, altering the exposure of VEGFR2 to specific phosphatases, as we recently demonstrated via a computational model of VEGFR2 signaling *in vitro* [20].

PlGF is not as well-studied as VEGF-A, in part because it is not required for normal murine development and homeostasis [21], and in part because PlGF binds only to VEGFR1, and not to VEGFR2, which is often considered to be the primary signaling receptor [22]. Like VEGF, PlGF has multiple splice isoforms, namely PlGF1 and PlGF2, with only the longer isoform (PlGF2) binding to ECM proteins strongly, and also to NRP1 [23, 24]. Despite being dispensable for murine development, PlGF expression is different in humans than mice [25], and increasing evidence implicates PlGF in disease [26]. Structural similarity also allows VEGF and PlGF to form heterodimers when produced in the same cells [27, 28]. There is high inter-study and intra-study variability in measurements of PlGF in human plasma and serum [29–41], many of which are from pregnant women, but levels of PlGF in healthy subjects are generally higher than those of VEGF-A (in 6 of 8 studies reviewed in [42] where both VEGF and PlGF in plasma or serum were measured [29–36]), and lower than those of soluble VEGFR1 (in 4 of 5 studies reviewed in [42] measuring both PlGF and sR1 in human plasma or serum [32, 34–37]).

VEGFR1 is also understudied compared to VEGFR2. VEGFR1 kinase activity appears to be weaker than that of VEGFR2, but VEGFR1 binds to VEGF more strongly than VEGFR2 [10]. While VEGFR1 kinase activity is not required for normal murine development [43], it appears to be important in the adult vasculature [44–46]. Like the VEGF ligands, VEGF receptors have alternative splice forms. Specifically, soluble VEGFR1 (sR1) is a naturally-occurring splice isoform lacking the transmembrane and intracellular domains but maintaining the ligand-, NRP1-, and HSPG-binding sites of VEGFR1. sR1 is secreted by endothelial cells into the extracellular space [47, 48]. There, sR1 can bind to the ECM [49] and/or bind to VEGF and PlGF, potentially preventing these growth factors from binding to cell surface receptors. Additionally, sR1 may heterodimerize with cell surface receptor monomers, forming non-signaling complexes [50]. While VEGF binding to VEGFR1 is thought by some to be anti-angiogenic, PlGF-induced VEGFR1 activation is generally considered to be pro-angiogenic [2]; the tyrosine phosphorylation patterns on VEGFR1 induced by VEGF and PlGF are different [44], and PlGF-VEGFR1 signaling is pro-angiogenic in zebrafish [51]. It has been hypothesized, based on *in vitro* data and overexpression studies, that PlGF and VEGFB binding to VEGFR1 induces pro-angiogenic effects by occupying VEGFR1, shifting VEGF from VEGFR1 to VEGFR2 [44, 52–54].

Though the contributions of VEGF, PlGF, growth factor immobilization, sR1, NRP1, VEGFR1, and VEGFR2 to VEGF-mediated signaling have all been studied *in vitro* (and to a limited extent *in vivo*), the combined regulation of these cues in the context of the human body is not well-understood. Compared to *in vitro* studies, physiological ligand concentrations are very low, many different growth factors are constantly being produced, consumed, and transported throughout the body, and the time-scales of interest are far longer [55]. Computational models provide a key tool to study the combined effects of many forms of regulation within a single framework, and to scale between model systems and human patients.

### Objectives

The primary objectives of this study were: (1) to predict the distribution of VEGF and PlGF within the body, (2) to understand the effect of VEGF and PlGF on the balance of VEGFR1 and VEGFR2 ligation and VEGFR2 phosphorylation, (3) to quantify the effect of matrix-bound VEGF & PlGF binding to endothelial and soluble receptors on VEGFR signaling, and (4) to study the impact of changes in VEGF & PlGF isoform expression on absolute and relative VEGFR1 & VEGFR2 activation and site-specific phosphorylation of VEGFR2, as a result of isoform-specific matrix- and NRP1-binding properties, all within the context of a healthy human body.

The computational systems pharmacology model developed in this study is based on previously-developed computational models of VEGF distribution and receptor binding *in vivo*. These models have included VEGF_165_, VEGF_121_, VEGFR1, VEGFR2, soluble VEGFR1 (sR1), NRP1, and sites in the interstitial matrix to which some growth factors and sR1 can bind [56–58]. The distribution of these proteins and their complexes has been examined in tissues of therapeutic interest (healthy or PAD calf [58], or tumor [59, 60]), the blood, and non-diseased tissue (main body mass) [56, 57], in humans or mice [61, 62], incorporating transport between these compartments via vascular permeability and lymphatic drainage of tissues, and clearance of proteins from the plasma. By including multiple tissue compartments, we can compare quantities in a tissue of interest to those in the bulk of body tissue.

In the present study, we greatly expand upon previous models to further capture the complexity of VEGF distribution and VEGF receptor activation in the body. For the first time, we include two isoforms of placental growth factor (PlGF1 & PlGF2), and the VEGF isoform VEGF_189_. Additionally, we account for binding of matrix-immobilized ligands in the endothelial basement membrane (EBM) to cell-surface receptors (VEGFR1 & VEGFR2), binding of immobilized ligands throughout the interstitial space to soluble sR1, and the ability of sR1, when sequestered in the interstitial matrix, to bind some VEGF isoforms. To capture these effects, we simulate receptor trafficking and VEGFR2 tyrosine site-specific phosphorylation following ligand binding or unbinding explicitly, implementing the reactions in a previously-developed *in vitro* computational model that captures differences in VEGFR2 phosphorylation following stimulation with soluble or matrix-bound VEGF_165_ [20]. Finally, we leverage recent measurements to update endothelial cell surface receptor densities [63].

## Methods

### Compartmental model formulation

**Fig 1:**
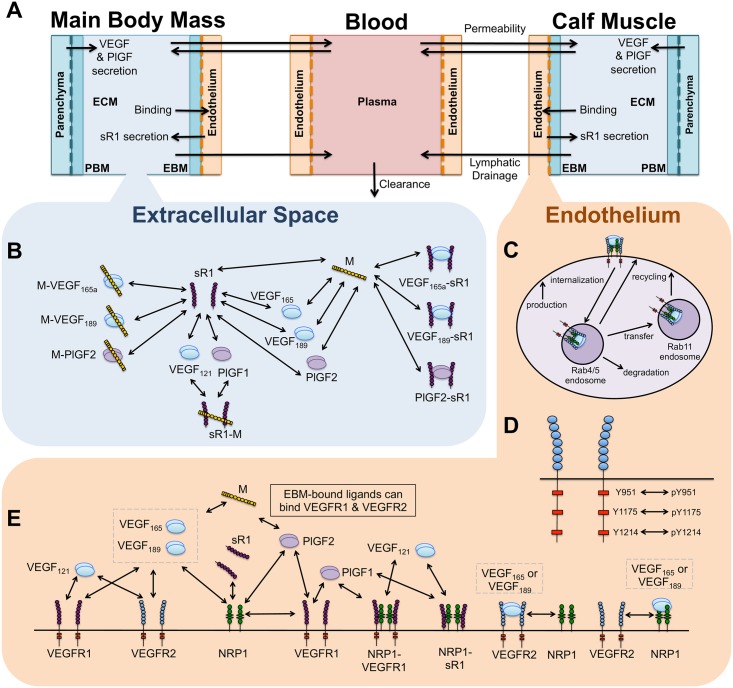
Schematics of molecular detail and structure of multi-scale computational model. **(A)** Whole-body compartmental model structure and mass flow. VEGF and PlGF are secreted from parenchymal cells, and sR1 is secreted by endothelial cells into the tissue interstitial space. Ligands and sR1 can then bind to EC receptors (leading to internalization and degradation), and can be transported between the tissue and blood via bi-directional vascular permeability or lymphatic draining of tissues into the circulation. Soluble species in the blood can be directly cleared from the blood. **(B)** Molecular interactions in tissue interstitial space between VEGF_121_, VEGF_165_, VEGF_189_, PlGF1, PlGF2, NRP1, sR1, and extracellular HSPGs/GAGs (M). It is assumed that, similar to NRP1-VEGFR1 complexes, VEGF_121_ and PlGF1 can bind to sR1-M. ECM-bound VEGF_165_, VEGF_189_, and PlGF2 can also bind to sR1. **(C)** Trafficking processes simulated in endothelial cells. **(D)** Site-specific phosphorylation and dephosphorylation of VEGFR2. **(E)** Abluminal (tissue-side) endothelial cell-surface molecular interactions between VEGF_121_, VEGF_165_, VEGF_189_, PlGF1, PlGF2, VEGFR1, VEGFR2, NRP1, sR1, and extracellular HSPGs/GAGs in the endothelial basement membrane (EBM).

To capture the pharmacokinetics of VEGF, PlGF, and sR1 distribution in the human body, we divide the body into three compartments: a healthy calf muscle (gastrocnemius + soleus muscles), blood, and the main body mass (the rest of the tissues), approximated with the properties of skeletal muscle (Fig 1A). Transport between compartments occurs via bi-directional vascular permeability and lymphatic drainage of tissues (into the blood), while growth factors and sR1 are cleared from the blood (via the liver and kidneys), using rates previously determined (S10 Table). Each tissue compartment includes physiological proportions of interstitial space, extracellular matrix (ECM), endothelium, other parenchymal cells, and basement membranes for both the endothelium and parenchyma (endothelial- EBM, and parenchymal- PBM).

Within each tissue, we incorporate molecularly-detailed pharmacodynamics, including secretion into the interstitial space of VEGF and PlGF by parenchymal cells and sR1 by endothelial cells. In the interstitium, these diffusible proteins can then bind to heparan sulfate proteoglycans (HSPGs) in the ECM and basement membranes (see Fig 1B, S11 Table), bind to receptors on endothelial cells (ECs), or be removed from the compartment via physiological transport processes (Fig 1A). VEGF and PlGF isoforms have different affinities for matrix sites and for the coreceptor NRP1, which are included (Table 1), to account for isoform-specific ligand distribution and receptor activation. On the surface of and within endothelial cells, we simulate binding of sR1 to NRP1, binding of PlGF to VEGFR1 and/or NRP1, and binding of VEGF to VEGFR1, VEGFR2, and/or NRP1, based on the binding properties of each protein (summarized in Tables 1–3 and Fig 1E).

**Table 1:** Binding/Unbinding reactions: K_D_.

K_D_	Description	VEGF_165_	VEGF_121_	VEGF_189_	PlGF1	PlGF2	Units	Ref
**L-R1**	Ligand binding to VEGFR1	3.3 x 10^−11^	3.3 x 10^−11^	3.3 x 10^−11^	**2.3 x 10**^**−10**^	**2.3 x 10**^**−10**^	M	[45, 57]
**L-R2**	Ligand binding to VEGFR2	1.0 x 10^−10^	1.0 x 10^−10^	1.0 x 10^−10^	**-**	**-**	M	[45, 57]
**L-N1**	Ligand binding to NRP1	1.2 x 10^−9^	-	**1.2 x 10**^**−10**^	**-**	**1.0 x 10**^**−7**^	M	[64, 65]
**L-sR1**	Ligand binding to sR1	3.3 x 10^−11^	3.3 x 10^−11^	3.3 x 10^−11^	**2.3 x 10**^**−10**^	**2.3 x 10**^**−10**^	M	[57]
**L-M**	Ligand binding to M (ECM/BM)	**6.1 x 10**^**−8**^	**-**	**6.1 x 10**^**−9**^	**-**	**4.6 x 10**^**−9**^	M	[66]
**(M-L)-R1**	M-bound ligand binding to R1	**3.3 x 10**^**−11**^	**-**	**3.3 x 10**^**−11**^	**-**	**2.3 x 10**^**−10**^	M	
**(M-L)-R2**	M-bound ligand binding to R2	**1.0 x 10**^**−10**^	**-**	**1.0 x 10**^**−10**^	**-**	**-**	M	
**(M-L)-sR1**	M-bound ligand binding to sR1	**3.3 x 10**^**−11**^	**-**	**3.3 x 10**^**−11**^	**-**	**2.3 x 10**^**−10**^	M	
**M-(L-R1)**	M binding to ligand-R1 complex	**6.1 x 10**^**−8**^	**-**	**6.1 x 10**^**−9**^	**-**	**4.6 x 10**^**−9**^	M	
**M-(L-R2)**	M binding to ligand-R2 complex	**6.1 x 10**^**−8**^	**-**	**6.1 x 10**^**−9**^	**-**	**-**	M	
**M-(L-sR1)**	M binding to L in L-sR1 complex	**6.1 x 10**^**−8**^	**-**	**6.1 x 10**^**−9**^	**-**	**4.6 x 10**^**−9**^	M	
**(L-sR1)-M**	M binding to sR1 in L-sR1	**-**	**2.4 x 10**^**−8**^	**-**	**2.4 x 10**^**−8**^	**-**	M	
**(M-sR1)-L**	Ligand binding to sR1 in M-sR1	**-**	**3.3 x 10**^**−11**^	**-**	**2.3 x 10**^**−11**^	**-**	M	
**(N1-L)-R2**	VEGFR2 binding to ligand-NRP1	1.0 x 10^−17^	-	1.0 x 10^−17^	**-**	**-**	moles/cm^2^	
**N1-(L-R2)**	NRP1 binding to ligand-VEGFR2	3.2 x 10^−17^	-	3.2 x 10^−17^	**-**	**-**	moles/cm^2^	
**(L-R1)-N1**	NRP1 binding to ligand-VEGFR1	-	1.0 x 10^−16^	-	**1.0 x 10**^**−16**^	**-**	moles/cm^2^	
**(L-sR1)-N1**	NRP1 binding to ligand-sR1	-	1.0 x 10^−16^	-	**1.0 x 10**^**−16**^	**-**	M	
**(N1-R1)-L**	Ligand binding to NRP1-R1	-	3.3 x 10^−11^	-	**2.3 x 10**^**−10**^	**-**	M	
**(N1-sR1)-L**	Ligand binding to NRP1-sR1	-	3.3 x 10^−11^	-	**2.3 x 10**^**−11**^	**-**	M	
								
**Other**	NRP1-VEGFR1 coupling	N1-R1	1.0 x 10^−16^	moles/cm^2^				[57]
	NRP1-sR1 coupling	sR1-N1	1.8 x 10^−9^	M				[64, 65]
	M binding to sR1	sR1-M	2.4 x 10^−8^	M				

Notes:

1. L: ligand, column-specific

2. Ordering shows where the bond is. For example: in M-(L-sR1): M binding to L for VEGF_165_, VEGF_189_, & PlGF2. Whereas, in (L-sR1)-M, M binding to sR1 for VEGF_121_, PlGF1.

3. All rates are the same inside endosomes as on cell surface. Unit conversions (see [20]) were required to convert all k_on_ (and thus K_D_) into context-specific units, as in previous compartment models. K_D_ in moles/cm^2^ = K_D_ in moles/L * (1 L/ 1000 cm^3^) * (1/ESAV) where ESAV is the endothelial surface area to volume ratio, given in S7 Table.

**Bold:** new parameters (to compartment model)

**Table 2:** Binding/Unbinding reactions: k_on_.

k_on_	VEGF_165_	VEGF_121_	VEGF_189_	PlGF1	PlGF2	Units
**L-R1**	3.0 x 10^7^	3.0 x 10^7^	3.0 x 10^7^	**1.5 x 10**^**6**^	**1.5 x 10**^**6**^	M^-1^ s^-1^
**L-R2**	1.0 x 10^7^	1.0 x 10^7^	1.0 x 10^7^	**-**	**-**	M^-1^ s^-1^
**L-N1**	5.0 x 10^5^	-	**1.4 x 10**^**6**^	**-**	**1.0 x 10**^**4**^	M^-1^ s^-1^
**L-sR1**	3.0 x 10^7^	3.0 x 10^7^	3.0 x 10^7^	**1.5 x 10**^**6**^	**1.5 x 10**^**6**^	M^-1^ s^-1^
**L-M**	**1.6 x 10**^**5**^	**-**	**1.6 x 10**^**5**^	**-**	**2.2 x 10**^**5**^	M^-1^ s^-1^
**(M-L)-R1**	**3.0 x 10**^**7**^	**-**	**3.0 x 10**^**7**^	**-**	**1.5 x 10**^**6**^	M^-1^ s^-1^
**(M-L)-R2**	**1.0 x 10**^**7**^	**-**	**1.0 x 10**^**7**^	**-**	**-**	M^-1^ s^-1^
**(M-L)-sR1**	**3.0 x 10**^**7**^	**-**	**3.0 x 10**^**7**^	**-**	**1.5 x 10**^**6**^	M^-1^ s^-1^
**M-(L-R1)**	**1.6 x 10**^**5**^	**-**	**1.6 x 10**^**5**^	**-**	**2.2 x 10**^**5**^	M^-1^ s^-1^
**M-(L-R2)**	**1.6 x 10**^**5**^	**-**	**1.6 x 10**^**5**^	**-**	**-**	M^-1^ s^-1^
**M-(L-sR1)**	**1.6 x 10**^**5**^	**-**	**1.6 x 10**^**5**^	**-**	**2.2 x 10**^**5**^	M^-1^ s^-1^
**(L-sR1)-M**	**-**	**4.2 x 10**^**5**^	**-**	**4.2 x 10**^**5**^	**-**	M^-1^ s^-1^
**(M-sR1)-L**	**-**	**3.0 x 10**^**7**^	**-**	**1.5 x 10**^**6**^	**-**	M^-1^ s^-1^
**(N1-L)-R2**	1.0 x 10^14^	-	1.0 x 10^14^	**-**	**-**	(moles/cm^2^)^-1^ s^-1^
**N1-(L-R2)**	3.1 x 10^13^	-	3.1 x 10^13^	**-**	**-**	(moles/cm^2^)^-1^ s^-1^
**(L-R1)-N1**	-	1.0 x 10^14^	-	**1.0 x 10**^**14**^	**-**	(moles/cm^2^)^-1^ s^-1^
**(L-sR1)-N1**	-	1.0 x 10^14^	-	**1.0 x 10**^**14**^	**-**	M^-1^ s^-1^
**(N1-R1)-L**	-	3.0 x 10^7^	-	**1.5 x 10**^**6**^	**-**	M^-1^ s^-1^
**(N1-sR1)-L**	-	3.0 x 10^7^	-	**1.5 x 10**^**6**^	**-**	M^-1^ s^-1^
						
**Other**	N1-R1	1.0 x 10^14^	(moles/cm^2^)^-1^ s^-1^			
	sR1-N1	5.6 x 10^6^	M^-1^ s^-1^			
	sR1-M	4.2 x 10^5^	M^-1^ s^-1^			

**Bold:** new parameters (to compartment model)

**Table 3:** Binding/Unbinding reactions: k_off_.

k_off_	VEGF_165_	VEGF_121_	VEGF_189_	PlGF1	PlGF2	Units
**L-R1**	1.0 x 10^−3^	1.0 x 10^−3^	1.0 x 10^−3^	**3.5 x 10**^**−4**^	**3.5 x 10**^**−4**^	s^-1^
**L-R2**	1.0 x 10^−3^	1.0 x 10^−3^	1.0 x 10^−3^	**-**	**-**	s^-1^
**L-N1**	6.0 x 10^−4^	-	**1.7 x 10**^**−4**^	**-**	**1.0 x 10**^**−3**^	s^-1^
**L-sR1**	1.0 x 10^−3^	1.0 x 10^−3^	1.0 x 10^−3^	**3.5 x 10**^**−4**^	**3.5 x 10**^**−4**^	s^-1^
**L-M**	**1.0 x 10**^**−2**^	**-**	**1.0 x 10**^**−3**^	**-**	**1.0 x 10**^**−3**^	s^-1^
**(M-L)-R1**	**1.0 x 10**^**−3**^	**-**	**1.0 x 10**^**−3**^	**-**	**3.5 x 10**^**−4**^	s^-1^
**(M-L)-R2**	**1.0 x 10**^**−3**^	**-**	**1.0 x 10**^**−3**^	**-**	**-**	s^-1^
**(M-L)-sR1**	**1.0 x 10**^**−3**^	**-**	**1.0 x 10**^**−3**^	**-**	**3.5 x 10**^**−4**^	s^-1^
**M-(L-R1)**	**1.0 x 10**^**−2**^	**-**	**1.0 x 10**^**−3**^	**-**	**1.0 x 10**^**−3**^	s^-1^
**M-(L-R2)**	**1.0 x 10**^**−2**^	**-**	**1.0 x 10**^**−3**^	**-**	**-**	s^-1^
**M-(L-sR1)**	**1.0 x 10**^**−2**^	**-**	**1.0 x 10**^**−3**^	**-**	**1.0 x 10**^**−3**^	s^-1^
**(L-sR1)-M**	**-**	**1.0 x 10**^**−2**^	**-**	**1.0 x 10**^**−2**^	**-**	s^-1^
**(M-sR1)-L**	**-**	**1.0 x 10**^**−3**^	**-**	**3.5 x 10**^**−4**^	**-**	s^-1^
**(N1-L)-R2**	1.0 x 10^−3^	-	1.0 x 10^−3^	**-**	**-**	s^-1^
**N1-(L-R2)**	1.0 x 10^−3^	-	1.0 x 10^−3^	**-**	**-**	s^-1^
**(L-R1)-N1**	-	1.0 x 10^−2^	-	**1.0 x 10**^**−3**^	**-**	s^-1^
**(L-sR1)-N1**	-	1.0 x 10^−2^	-	**1.0 x 10**^**−3**^	**-**	s^-1^
**(N1-R1)-L**	-	1.0 x 10^−3^	-	**3.5 x 10**^**−4**^	**-**	s^-1^
**(N1-sR1)-L**	-	1.0 x 10^−3^	-	**3.5 x 10**^**−4**^	**-**	s^-1^
						
**Other**	N1-R1	1.0 x 10^−2^	s^-1^			
	sR1-N1	1.0 x 10^−2^	s^-1^			
	sR1-M	1.0 x 10^−2^	s^-1^			

**Bold:** new parameters (to compartment model)

Endothelial cell surface receptors are continually produced, internalized, recycled, and degraded, with trafficking rates that depend on ligation status and complex formation with NRP1 (Fig 1C). We include detailed VEGFR2 trafficking based on a previous *in vitro* computational model (S8 Table). Surface receptor production rates were tuned to match experimental measurements of cell surface receptor levels in human umbilical vein endothelial cells (Table 4). We also explicitly include phosphorylation and site-specific dephosphorylation of VEGFR2 (Fig 1D), which is dependent on receptor trafficking, with higher net activation at Y1214 than Y1175 on the cell surface, and higher Y1175 phosphorylation in early (Rab4/5) endosomes (S9 Table), as a result of differential dephosphorylation of Y1175 and Y1214 on the cell surface and in early endosomes [20]. This allows us to study phosphorylation explicitly, instead of using receptor occupancy as a surrogate, and to look at relative activation of downstream signaling pathways leading to proliferation (pY1175 via ERK1/2) and migration (pY1214 via p38).

**Table 4:** Targets & secretion/production rates at steady-state.

Species	Target Location	Target Value	Target Units	Fit Production/Secretion Rates	Production Units	Ref
**VEGFR1**	Main Body Mass	1800	Surface receptors/EC	**1.162**	Change from No VEGF SS	[67]
	Calf	1800	Surface receptors/EC	**1.32**	Change from No VEGF SS	[67]
**VEGFR2**	Main Body Mass	5800	Surface receptors/EC	**32.09**	Change from No VEGF SS	[67]
	Calf	5800	Surface receptors/EC	**53.95**	Change from No VEGF SS	[67]
**NRP1**	Main Body Mass	70,000	Surface receptors/EC	**1.295**	Change from No VEGF SS	[63]
	Calf	70,000	Surface receptors/EC	**1.502**	Change from No VEGF SS	[63]
**sR1**	Plasma	100	pM	0.0893	molec/EC/s	[57]
**PlGF**	Plasma	10	pM	**0.0146**	molec/MD/s	[42]
PlGF1				**15%**	% of Prod	[68]
PlGF2				**85%**	% of Prod	[68]
**VEGF**	Plasma	1.5	pM	0.2830	molec/MD/s	[57]
VEGF_165_				77%	% of Prod	[69]
VEGF_121_				8%	% of Prod	[69]
VEGF_189_				**15%**	% of Prod	[69]

**Bold:** new parameters (to compartment model)

SS: steady-state

MD: myonuclear domain (portion of a skeletal muscle myocyte associated with a single nucleus)

Due to the spatially-averaged nature of this model, gradients and heterogeneity in growth factor, soluble receptor, and cell surface receptor patterning are neglected. Instead, we examine the tissue-averaged behavior within the context of the human body. We neglect secretion of sR1 directly into the bloodstream, receptors present on the luminal side of ECs, and degradation of growth factors by proteases. All parameters are based on or fit to experimental data, either newly here or previously for other computational models. By building on previous modeling efforts, we have built more molecular detail into our models, while adding only a modest number of new parameters (indicated in bold in Tables 1–4 and S7 Table).

To simulate the time-course of each molecular species in each tissue and the blood, this model includes 635 nonlinear ordinary differential equations that are solved simultaneously. The model equations can be found in S1 Equations. The full set of differential equations was solved in Fortran using the Livermore Solver for Ordinary Differential Equations with Automatic method switching for stiff and nonstiff problems (LSODA), on a laptop PC, with a relative error tolerance of 10^−6^.

### Model parameterization

#### Geometry

The geometric parameterization is taken, without modification, from a previous 3-compartment model of a healthy 70 kg human [57], and is detailed in S7 Table. Briefly, histological cross-sections of human gastrocnemius muscle and vastus lateralis muscle were used to parameterize the “calf muscle” and “main body mass” compartments, respectively. These cross-sections and other measurements were used to estimate the relative fractions of muscle volume occupied by myocytes, capillaries (separated into vascular space and endothelium), and interstitial space. Estimates of endothelial and myocyte basement membrane thickness, cell surface areas and volumes, and the volume fractions of ECM protein and fluid in interstitial space were also used to parameterize the tissue compartments. For full details, see [57]. The blood is taken to be 5L, with 60% of that volume being plasma.

#### Binding and coupling kinetics

In this model, we include five growth factor ligands (L), each with different receptor-binding, matrix-binding, and NRP1-binding properties (Fig 1B and 1E). Our goal is to understand how these isoform-specific properties lead to differential ligation and activation of VEGFR1 and VEGFR2. We assume all ligands and receptors are pre-dimerized, neglecting the formation of ligand or receptor heterodimers, and assume the same binding properties for sR1 as endothelial VEGFR1 [70]. NRP1 can bind directly to VEGFR1 (and we assume sR1) [71], while VEGF is required to bridge NRP1 and VEGFR2. While VEGF binds to both VEGFR1 and VEGFR2, PlGF binds to only VEGFR1. The shorter PlGF1 does not bind to NRP1 or to the matrix (M), but we assume that PlGF1, like VEGF_121_, does bind to VEGFR1 and NRP1 simultaneously. VEGF_121_ does not bind to the matrix, and its ability to bind NRP1 [72] alone is neglected, as it has previously been shown to have very little effect on VEGFR signaling *in vivo* [57]. For both PlGF and VEGF, the longer isoforms (VEGF_165_, VEGF_189_, and PlGF2) bind to the matrix, PlGF2 more strongly than VEGF_165_ [66]. These longer isoforms also bind NRP1, but not NRP1-VEGFR1 complexes (though this remains unproven for PlGF2). Reflecting our previous *in vitro* computational model, we account for binding of matrix-bound ligands to VEGFR2 (previously demonstrated [18, 19]) and VEGFR1 (assumed to occur). We assume that endothelial basement membrane-bound growth factor within 25nm of the cell surface is accessible to cell surface receptors, based on the length of the extracellular domain of the related RTKs ErbB2 and ErbB3 (11.3–16.4nm) [73–75], and assuming some flexibility in cell position and shape. We calculated the resulting fraction of EBM accessible to cell surface receptors (S7 Table), and scaled the corresponding reaction on-rates (Table 2, see S1 Equations). Similarly, we allow matrix-immobilized VEGF_165_, VEGF_189_, or PlGF2 to bind to sR1, creating matrix-ligand-sR1 (M-L-sR1) complexes, which cannot bind cell surface receptors, and are therefore effectively sequestered. As VEGFR1 can bind to NRP1 without ligand, and the NRP1- and heparin-binding domains of VEGFR1 overlap, we also examine the impact of allowing matrix-bound sR1 to bind VEGF_121_ and PlGF1 in the interstitial space, allowing these non-matrix-binding ligands to be sequestered. In all cases, in the absence of evidence to the contrary, we assume that matrix-immobilization does not affect the affinity of any interactions.

The binding and unbinding rates for VEGF and PlGF to VEGFR1, VEGFR2, and sR1 are kept the same as in previous models [45, 57], as summarized in Tables 1–3 (new parameters in bold). Though we have not previously included PlGF in a compartment model, PlGF binding to VEGFR1 has been modeled *in vitro* [45], and the parameter values are matched to this study. The affinity of PlGF2 for NRP1 is based on experimental measurements of PlGF2 binding to the NRP1 extracellular domain [65]. Slightly different affinities are used for VEGF binding to matrix sites and to NRP1 than in previous compartment models, in order to use measurements from a single source for both VEGF_165_ and VEGF_189_ (NRP1-binding) [64], or for VEGF and PlGF (matrix-binding) [66]. Since VEGF_189_ is known to bind the ECM more strongly than VEGF_165_, but an affinity is not available, we assume 10x stronger binding, similar to the difference in VEGF_165_ and VEGF_189_ affinity for NRP1 [64]. As in previous models [57], lacking a measured affinity for sR1 binding to matrix, we assume a value similar to that for VEGF, as both interactions occur via heparin-binding domains.

#### Receptor trafficking and VEGFR2 phosphorylation

We added receptor trafficking and VEGFR2 phosphorylation to the model, in order to track site-specific phosphorylation of VEGFR2 explicitly, rather than simply receptor occupancy. This is more accurate, as *in vitro* VEGFR2 phosphorylation decreases faster than can be accounted for by ligand depletion or receptor degradation [20]. We implemented these reactions as previously described in an *in vitro* model [20] for VEGFR2, accounting for ligand-induced changes in internalization, recycling, and degradation, as well as preferential recycling of VEGFR2 complexes containing NRP1 via a Rab11-dependent pathway. The trafficking rate constants are given in S8 Table. Though VEGFR1 trafficking is known to be distinct from that of VEGFR2 [76, 77], we lack sufficient data to build or validate a model of VEGFR1 trafficking. As such, a structure for VEGFR1 trafficking was incorporated for future use, but results are presented only for cell surface VEGFR1.

Site-specific phosphorylation of VEGFR2 on three tyrosine sites is included: Y951, Y1175, and Y1214. We approximate phosphorylation and dephosphorylation as first order processes, and assume that these processes occurred independently on each tyrosine. The phosphorylation rate is assumed to be zero for unoccupied VEGFR2, and fast (1 s^-1^) for ligated VEGFR2. The dephosphorylation rates do not depend directly on the VEGF isoform, but vary by tyrosine site and subcellular location (S9 Table), as previously fit and validated [20] using experimental observations of increased pY1214 following stimulation with immobilized VEGF compared to free VEGF in solution [18], and enabling site-specific phosphorylation patterns to depend on the mixture of matrix- binding and non-matrix-binding isoforms available to VEGFR2. Given limited data available for phosphorylation of Y951 upon which to fit the model, this analysis focuses on VEGFR2 activation on Y1175 and Y1214.

#### Transport

Inter-compartmental transport parameters are taken from a previous model [57] (see S10 Table). Vascular permeability was estimated based on the Stokes-Einstein radii for each protein. Here, we assume the same permeability for PlGF as VEGF, as they have similar molecular weights and are structurally related. Lymphatic drainage transports proteins from tissue compartments to the blood in a tissue-mass-dependent and a protein-size-independent fashion. We use the estimated lymphatic flow rates for a supine, awake 70 kg human [57].

#### Protein expression levels

We assume the same densities of interstitial matrix sites available to bind VEGF, PlGF, and sR1 in the ECM and basement membranes as used in previous models [57] (see S11 Table). Briefly, ECM binding-site density is based on measured FGF binding sites [78, 79], while basement membrane binding site densities are estimated based on Engelbreth-Holm-Swarm sarcomas in diabetic mice [80]. Endothelial cell surface VEGFR1, VEGFR2, and NRP1 target levels were chosen to match median experimental (FACS) measurements in human umbilical vein endothelial cells [63, 67], which represented our best information to date on receptor levels in humans; these values are summarized in Table 4. Total receptor levels are not directly controlled, but remain within a reasonable range. The VEGF and PlGF secretion rates by myocytes and endothelial secretion of sR1 into the interstitial space were adjusted to match experimentally measured plasma protein levels (Table 4). Plasma levels are used as targets because no interstitial measurements of sR1 or PlGF levels are available, and plasma VEGF levels are better characterized than tissue interstitial levels. Target levels of plasma VEGF and sR1 are unchanged from previous models [57], and a plasma PlGF target concentration of 10pM was selected. The secretion of different VEGF isoforms and PlGF isoforms are maintained at fixed ratios, based on experimental measurements in mice (VEGF) and humans (PlGF) [68, 69]. Production rates for VEGFR1, VEGFR2, and NRP1 were adjusted independently in the calf muscle and the main body mass to meet target values in each tissue while also meeting plasma ligand targets. As VEGF, PlGF, and sR1 secretion are fit only to plasma measurements, we assume the same secretion rates per cell in both tissue compartments.

## Results

### Ligand secretion and receptor production rates for baseline typical healthy human

The ligand secretion and receptor production rates necessary to hit baseline (healthy) targets had to be fit simultaneously, due to the highly non-linear nature of the system. At our baseline steady-state, the VEGF production rate is 0.2830 molecules/myonuclear domain/s, the PlGF production rate is 0.0146 molecules/myonuclear domain/s, and the sR1 production rate is 0.0893 molecules/EC/s (see Table 4). The VEGF and sR1 production rates here are higher than previous estimates. This is unsurprising, given the changes in receptor levels, trafficking, and growth factor isoforms. Surprisingly, the PlGF production rate is lower than that for VEGF, despite a higher target plasma level (see Flux Analysis section for the mechanism by which this occurs).

**Fig 2:**
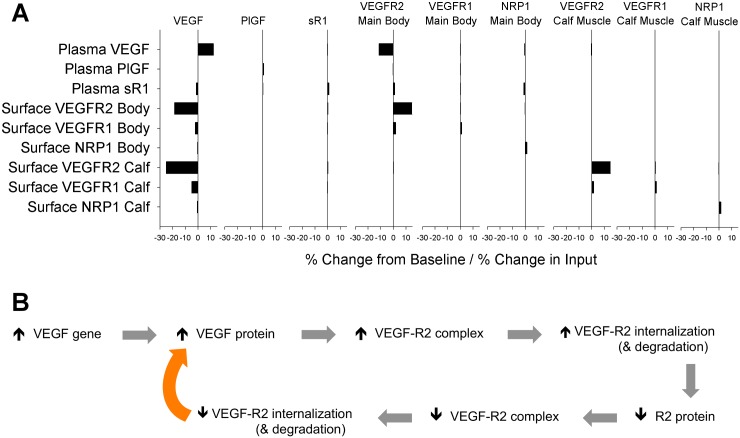
Nonlinearity of ligand & sR1 secretion and EC receptor production rates in the model. **(A)** One at a time, each baseline ligand secretion or receptor production rate (inputs- listed across the top), was increased by 2%, then decreased by 2%. For each perturbation, the change in plasma ligand and EC surface receptor levels (outputs- listed on the left) in in both the main body mass (“Body”) and calf muscle (“Calf”) were obtained. The average change in output from baseline levels was calculated, and divided by the change in input (+/-2%) to give the relative change in output per % change in input. **(B)** Schematic of positive feedback in VEGF gene and protein levels in the model. An increase in VEGF expression increases local VEGF protein, increasing VEGF binding to VEGFR2, and subsequent internalization and degradation. This decreases total VEGFR2 protein levels, leading to reduced VEGF-VEGFR2 complex formation, which reduces net endothelial consumption of VEGF protein. To accommodate, in the model, VEGFR2 expression was increased until target baseline levels were achieved for all ligands and receptors. A similar positive feedback loop exists for changes in VEGFR2 expression.

To illustrate the nonlinearity of our model, we perturbed each ligand secretion and receptor production rate slightly (2%), and examined changes in plasma ligand and tissue receptor levels. As shown in Fig 2A, plasma VEGF and tissue VEGFR2 are highly sensitive to changes in either VEGF secretion or VEGFR2 production in the main body mass, with changes of 11–25% per percent change in input. As VEGF levels increase, more VEGFR2 becomes occupied, internalized, and degraded, reducing VEGFR2 levels and decreasing VEGF consumption (Fig 2B and S1 Fig). Similarly, as VEGFR2 production increases, more VEGF is bound to VEGFR2, internalized, and degraded, reducing VEGF levels and thus increasing EC surface VEGFR2. This super-sensitivity was not present in previous models, where surface VEGFR2 levels were fixed (see S1 Fig). This new, emergent result suggests that, lacking upregulation of VEGFR2 in response to VEGF, VEGFR2 levels would be highly sensitive to even small fluctuations in local VEGF concentration (Fig 2), highlighting the importance of dynamic adjustments to ligand and receptor expression *in vivo*. In the calf muscle, perturbing VEGFR2 production has a large impact on EC surface VEGFR2, but little effect on plasma VEGF, due to the smaller size of the compartment. Changes in receptor production in one tissue compartment have little effect on receptor levels in the other tissue compartment.

**Fig 3:**
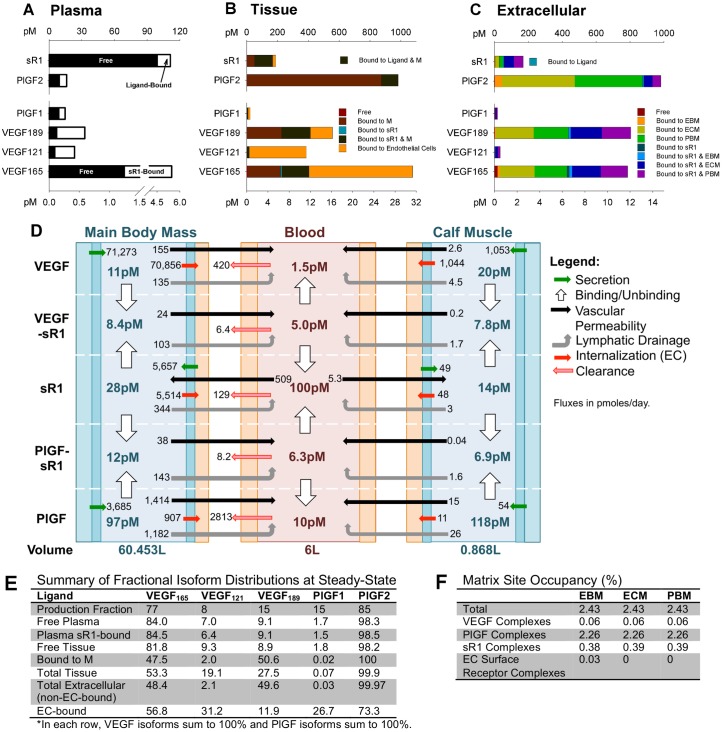
Pharmacokinetics of VEGF, PlGF, and sR1 at steady-state. **(A)** Predicted free and sR1-bound ligands, and free and ligand-bound sR1 in plasma. **(B)** Predicted VEGF, PlGF, and sR1 distribution in healthy tissue in “Main Body Mass” compartment, shown in pM of tissue. **(C)** Extracellular (not bound to or inside ECs) VEGF, PlGF, and sR1 in “Main Body Mass” compartment, in pM of tissue. **(D)** Steady-state net flow profiles for VEGF, PlGF, sR1, and sR1-ligand complexes between the calf muscle, blood, and main body mass. All VEGF isoforms are aggregated, as are both PlGF isoforms. Green arrows represent production, red arrows EC consumption, black arrows bi-directional vascular permeability, gray arrows lymphatic drainage, and pink arrows with red outlines direct clearance from blood. The white arrows show the net association or dissociation of VEGF-sR1 and PlGF-sR1 complexes in each compartment. Displayed concentrations are free ligand, sR1, or complex in interstitial fluid or plasma. The numbers under each compartment are the respective compartment volumes. Flows are given in pmoles/day. **(E)** Comparison of VEGF and PlGF isoform distribution with relative isoform production rates demonstrates locations and complexes where each isoform is under- or over-represented relative to the fraction of total VEGF or PlGF production. **(F)** Matrix site occupancy in the EBM, ECM, and PBM.

In this model, we assume the same rates for ligand production in both the healthy calf muscle and the main body mass. As such, perturbing the VEGF secretion rate (in both compartments) alters the receptor levels in both tissues (Fig 2). Due to differences in the geometric parameterizations of the calf and other tissues (S7 Table), using the same ligand secretion rates results in different interstitial VEGF, sR1, and PlGF levels (Fig 3D). We focus primarily on quantities measured in the “Main Body Mass” compartment, which, due to its larger size, represents the primary determinant of plasma VEGF, sR1, and PlGF levels.

### Pharmacokinetics: Where are VEGF, PlGF, and sR1 in the body?

After establishing the secretion and production rates required to achieve basal targets, we next examined the steady-state distribution of VEGF, PlGF, and sR1.

#### Plasma: Differential isoform representation compared to relative expression levels

In the plasma, free VEGF protein is predicted to be 84% VEGF_165_, 7% VEGF_121_, and 9% VEGF_189_; thus VEGF_189_ (the strongest ECM-binding isoform) is underrepresented compared to the production fractions of 77%, 8%, and 15%, respectively (Fig 3A and 3E). Conversely, the ECM-binding PlGF2 isoform is overrepresented in plasma (98% of free plasma PlGF), compared to its production (85% of PlGF production), reflecting its overrepresentation in the tissue extracellular space (see Fig 3). In agreement with previous models, 77% of plasma VEGF and 39% of PlGF are bound to sR1. A total of 10% of plasma sR1 is bound to ligand, with 44% of this bound to VEGF and 56% bound to PlGF, suggesting that PlGF interacts with sR1 to a comparable extent as VEGF.

#### Tissue (Main Body Mass): ECM-binding drives distinct VEGF & PlGF isoform distribution

The model predicts that the total and relative levels of matrix-bound and free growth factor are dictated by ECM binding properties (Fig 3C). While the model predicts that the majority of VEGF_121_, VEGF_165_, and PlGF1 are bound to endothelial cells (96%, 62%, and 58%, respectively- see Fig 3B) in the main body mass, large portions of the heparin-binding isoforms, VEGF_165_, VEGF_189_, and PlGF2, are bound to the ECM and basement membranes (36%, 74%, and 99.6% of total in tissue, respectively), alone or in complex with sR1 (Fig 3B). Most of the immobilized growth factor is in the ECM and parenchymal BM (Fig 3C), inaccessible to EC receptors, but available for proteolytic release. Total extracellular (non-EC-bound) VEGF is 48% VEGF_165_, only 2% VEGF_121_, and 50% VEGF_189_, while extracellular PlGF is 99.97% PlGF2 (Fig 3E). As these percentages suggest, most extracellular heparin-binding growth factor is matrix bound (alone or in complex with sR1): 96% of VEGF_165_, 99.6% of VEGF_189_, and 99.7% of PlGF2. However, 93% of VEGF_121_ and 80% of PlGF1 are also sequestered (via immobilized sR1) in our simulations. The total amount of sequestered VEGF_121_ and PlGF1 is small (Fig 3C), but still significant compared to the corresponding free growth factor concentrations in solution. Indeed, only 7.8% of tissue PlGF1 and <1% of every other isoform is predicted to be “free” in solution. This is consistent with previous results [57] in suggesting that, unlike cell culture experiments, ligand-receptor binding is limited by ligand availability in the body. The model predicts that 90% of sR1 in tissue is matrix-bound (Fig 3B), while only 0.45% is free (bound to neither matrix nor ligand), and 0.32% bound to ligand alone, implicating the ECM in regulation of sR1 distribution as well.

**Fig 4:**
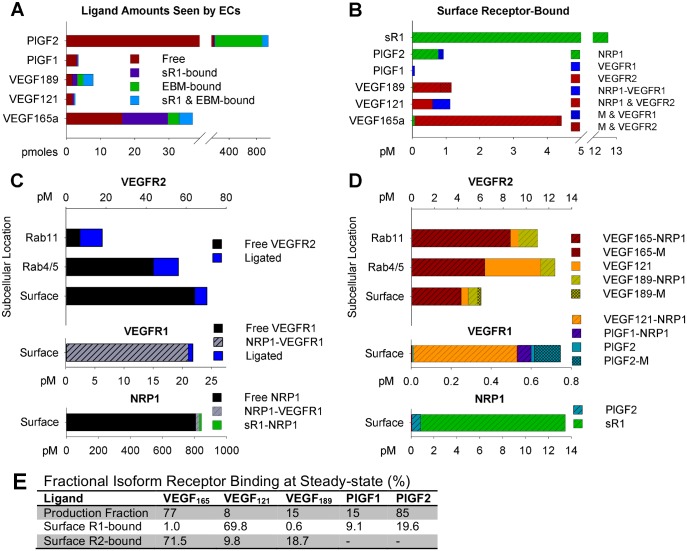
Pharmacodynamics of ligand binding to VEGFR1 and VEGFR2. **(A)** Total soluble growth factor (in available interstitial fluid) and immobilized growth factor (in innermost 25nm of EBM) accessible to ECs. Growth factor bound to EC receptors is not included in this plot. **(B)** Break-down of EC surface-bound ligand, by isoform. Note the difference in quantities of total ligated VEGFR2, VEGFR1, and NRP1 (**panel C**). **(C)** Occupancy of VEGFR2, VEGFR1, and NRP1 on ECs, broken down by ligand and NRP1-binding. VEGFR2 occupancy is shown on the cell surface, in early signaling endosomes (Rab4/5), and in recycling endosomes (Rab11), while VEGFR1 and NRP1 are shown only on the cell surface. Quantities are given in pM of total tissue in the “Main Body Mass” compartment. **(D)** VEGFR2, VEGFR1, and NRP1 ligation on ECs, excluding receptor not bound to ligand. Complexes not listed in the legend are present at levels too low to be seen in the figure. **(E)** Break-down of percentage of EC surface VEGFR1 and VEGFR2 ligation comprised by each isoform, compared to the relative production of each isoform. Production fractions are calculated separately for VEGF and PlGF, while for receptor binding the combined distribution is shown.

While a large fraction of growth factor is immobilized, predicted matrix site occupancy is low (2.4%- see Fig 3F). This is higher than in previous models, as a result of the inclusion of PlGF and immobilized complexes containing both growth factor and sR1. In the endothelial BM, most (93%) occupied sites contain PlGF; 16% contain sR1, and 2.3% VEGF. While only 1.1% of occupied EBM sites include ligand bound to cell surface receptors, the large number of binding sites in the endothelial BM makes even this small fraction physiologically relevant (see Fig 4).

#### Flux analysis: Differential transport of VEGF & PlGF

By calculating the net transport, consumption, and clearance of each protein or complex (Fig 3D), we can examine the contributions of each dynamic process to the steady-state distribution. At steady-state, the model predicts a concentration of 11pM VEGF in the available interstitial fluid of the main body mass, similar to previous models. The levels in the calf muscle are higher (20pM), due to a higher myocyte volume fraction and resulting higher production per unit tissue volume. While other quantities also varied between the two compartments, all trends and net flux directions were the same. In agreement with previous model predictions, free sR1 levels are higher in plasma than in tissue, while PlGF levels, like VEGF levels, are higher in tissue. These concentration differences lead to predicted transendothelial intravasation (net transfer from tissue to blood) of VEGF and PlGF, while free sR1 is predicted to extravasate (net transfer from blood to tissue). The fraction of sR1 bound to ligand is similar in plasma and tissue interstitial fluid (42% in the main body mass, 51% in calf muscle), with substantial contributions by both VEGF and PlGF. The large majority of VEGF and sR1 produced are consumed locally by endothelial cells (99% of VEGF and 98% of sR1 in the “Main Body Mass”), accounting for the high sensitivity of interstitial VEGF to VEGFR2 production (see Fig 2). Conversely, the model predicts that only 25% of PlGF is consumed by ECs, due to much lower total binding to EC receptors than VEGF. This accounts for the low PlGF production rate required to match target plasma levels, and suggests that PlGF may be primarily cleared via transendothelial transport and lymphatic drainage into plasma, followed by clearance from the blood, or by cell types not included in this model (e.g. monocytes & macrophages).

### Pharmacodynamics: What controls VEGFR1 and VEGFR2 activation?

Having examined the distribution of VEGF, PlGF, and sR1, we next zoomed in to examine the effect of these proteins and their distributions on the binding and activation of endothelial VEGFR1 and VEGFR2 within healthy tissue.

#### Growth factors levels are limiting for *in vivo* EC receptor activation

At steady state, cell surface ligation of VEGFR2 is predicted to be close to an order of magnitude higher than cell surface ligation of VEGFR1 (Fig 4D), due in part to higher levels of EC surface VEGFR2 (5800 VEGFR2/cell vs. 1800 VEGFR1/cell). As a result, the majority of EC consumption of VEGF occurs via VEGFR2, explaining why VEGF levels are more sensitive to changes in production of VEGFR2 than VEGFR1 (Fig 2). Overall, the model predicts low cell surface receptor occupancies of 3.4% for VEGFR1 and 8.7% for VEGFR2 (4.5% VEGFR1 and 14% VEGFR2 in calf muscle), and somewhat higher but still low total (surface + endosomal) VEGFR2 occupancy (20%), suggesting that ligands do not compete for receptor binding (Fig 4C). This prediction is conservative; model VEGF levels are in fact higher than estimates of free interstitial VEGF via microdialysis, and plasma target levels for VEGF and PlGF assume that no sR1-bound ligand was detected. While sR1 is known to interfere with VEGF ELISA measurements, likely at least a portion of this bound VEGF is in fact detected, thus placing our calibrated model at the top of the possible VEGF range.

#### NRP1- & ECM-binding drive VEGF & PlGF isoform binding to VEGFR1 and VEGFR2

The majority of non-ligand-bound VEGFR1 is predicted to be in complex with NRP1 (99.1%). NRP1 remains mostly free (95.3%) (Fig 4C), with some binding to sR1 and PlGF2 to form non-signaling complexes (Fig 4D). The isoform-specific NRP1 binding properties of VEGF and PlGF make NRP1 a strong regulator of ligand-binding to VEGFR1 and VEGFR2. The model predicts that VEGF_165_ and VEGF_189_, which bind to VEGFR2 and NRP1 simultaneously, bind almost exclusively to VEGFR2 (Fig 4D). Conversely, VEGF_121_, which binds to NRP1-VEGFR1 complexes, comprise 70% of ligand bound to VEGFR1 (Fig 4D), while PlGF makes up only 29% of the ligand bound to VEGFR1 at steady-state (Fig 4E). This result explains the lower predicted occupancy of VEGFR1 than VEGFR2; VEGF_121_ and PlGF1, the only ligands to bind VEGFR1 and NRP1 simultaneously, represent a small fraction of total ligand (Fig 4A). The dominance of VEGF_121_ binding to endothelial VEGFR1 is in contrast to the relatively even binding of VEGF and PlGF to sR1 (Fig 2), and occurs because most tissue PlGF is PlGF2, which cannot bind to NRP1-VEGFR1 complexes on endothelial cells.

While all soluble growth factors are accessible to EC receptors in this model (assuming a well mixed compartment, i.e. nonlimiting fast diffusion), cell surface receptors are only allowed to bind to immobilized ligands in the innermost 25nm of endothelial BM. A substantial fraction of both soluble and endothelial BM-bound growth factor is bound to sR1, and thus inaccessible to EC receptors (Fig 4A). Of the remaining growth factor, the model predicts that the amount of available free growth factor exceeds the amount of available immobilized growth factor for all VEGF isoforms, but not for PlGF2 (Fig 4A) However, within the 25nm space adjacent to endothelial cells, the concentration of available immobilized growth factor far exceeds the predicted concentration of free growth factor for all matrix-binding isoforms (S2A Fig).

Of the 0.03% of basement membrane sites bound to ligand-cell surface receptor complexes, 23% are immobilized PlGF2 bound to VEGFR1, 20% are VEGF_165_-R2 complexes, and 56% are VEGF_189_-R2 complexes. While more of these complexes are bound to VEGFR2, VEGFR1 has a higher fraction of ligand-receptor complexes bound to immobilized ligands (18% versus 6.9%- see Fig 4D). This is due the lower total number of ligand-VEGFR1 complexes, combined with higher tissue levels and stronger matrix binding by PlGF2 compared to VEGF. If we assumed all endothelial BM-bound growth factors were accessible to receptors (as opposed to the closest 25nm), 50% of ligated VEGFR1 would be bound to immobilized PlGF, and 17% of ligated VEGFR2 would be bound to immobilized VEGF_165_ or VEGF_189_.

#### NRP1 regulates isoform-specific trafficking and phosphorylation of VEGFR2

In addition to guiding receptor ligation, NRP1 also regulates VEGFR2 trafficking [11], speeding up recycling of ligated VEGFR2. This leads to predicted accumulation of VEGF_121_-VEGFR2 complexes in early signaling (Rab4/5) endosomes, while VEGF_165_-VEGFR2 and VEGF_189-_VEGFR2 are recycled back to the cell surface, leading to a more even distribution between the cell surface and early endosomes (Fig 4D). As such, changes in relative levels of VEGF isoforms are predicted to alter not only the tissue distribution of ligand and the balance of VEGFR1 and VEGFR2 activation, but also the subcellular localization of VEGFR2.

**Fig 5:**
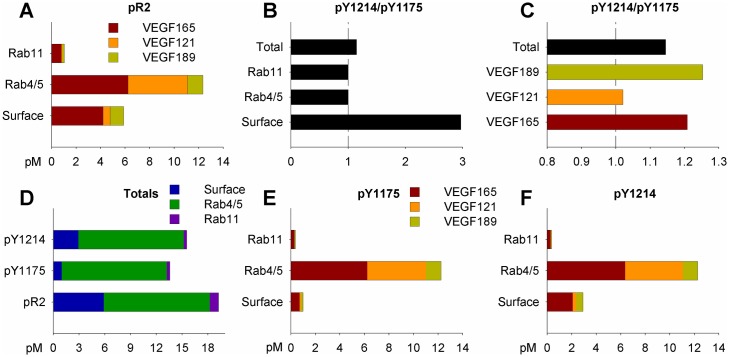
VEGF isoform-specific trafficking and site-specific phosphorylation of VEGFR2 *in vivo*. **(A)** VEGF isoform-specific NRP1-binding properties result in isoform-specific trafficking of VEGFR2. **(B)** Subcellular location-specific dephosphorylation rates for Y1175 and Y1214 (S9 Table) lead to preferential activation of tyrosine 1214 on the EC surface, compared to signaling in endosomes. **(C)** Isoform-specific trafficking and location-specific dephosphorylation combine to result in isoform-specific trends in relative activation of VEGFR2 on tyrosine 1175 and tyrosine 1214. **(D)** Total VEGFR2 phosphorylation, on at least one tyrosine (pR2) and specifically on Y1175 or Y1214, across all subcellular locations. **(E-F)** Distribution of pY1175 (**E**) and pY1214 (**F**), by VEGF isoform and location.

We previously showed that changes in site-specific phosphorylation of VEGFR2 as a function of VEGF_165_ immobilization to a surface or in a gel could be explained by prolonged retention of immobilized VEGF-VEGFR2 complexes at the cell surface [20], increasing net phosphorylation on Y1214 and promoting pro-migratory signaling. Here, we examined whether this translated to VEGF isoform-specific trends in site-specific phosphorylation of VEGFR2 in a physiological context. Indeed, we see that the faster dephosphorylation of tyrosine Y1175 than Y1214 on the cell surface, and vice versa in early (Rab4/5) signaling endosomes (Fig 5B), leads to different relative levels of VEGFR2 activation on Y1175 and Y1214 as a function of the bound ligand; the heparin-binding VEGF isoforms (VEGF_165_ and VEGF_189_) lead to higher net activation on Y1214, while VEGF_121_ shifts relative activation towards Y1175 (Fig 5C).

### Complex, coordinated regulation of VEGFR1 and VEGFR2 signaling

It is clear that the different proteins—ligands, soluble receptors, and co-receptors—regulating VEGFR1 and VEGFR2 activation do not act in isolation. Changes to any single feature affect the total multi-factor system in a way that is difficult to predict without the use of a computational model. Here, we perturb several interactions that are of interest therapeutically, and/or are included in this model for the first time.

#### PlGF does not displace VEGF from VEGFR1 to increase VEGFR2 signaling *in vivo*

**Fig 6:**
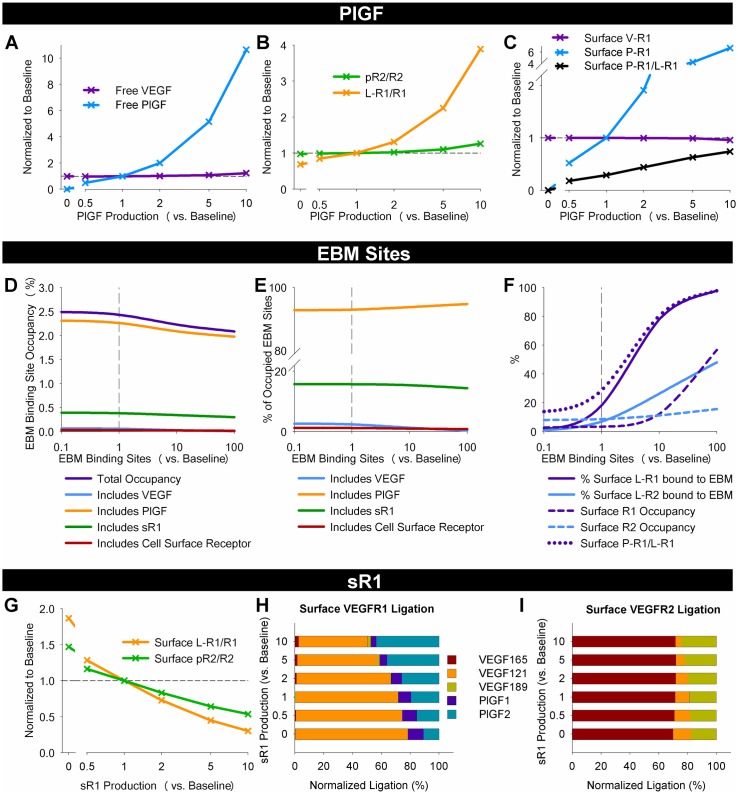
Complex regulation of VEGF family signaling by PlGF, EBM binding sites, and sR1. **(A-C)** Changes in free ligand levels in tissue interstitial fluid (**A**), EC surface VEGFR1 ligation and VEGFR2 phosphorylation (**B**), and the breakdown of VEGF and PlGF bound to EC surface VEGFR1 (**C**), in response to varying PlGF production. Quantities shown are normalized to baseline cases. **(D-F)** Effect of endothelial basement membrane (EBM) binding site density on EBM site occupancy (**D**), fraction of occupied EBM sites bound to different ligands and receptors (**E**), and VEGFR1 and VEGFR2 ligation by immobilized VEGF or PlGF (**F**). **(G-I)** Total activation of VEGFR1 and VEGFR2 (**G**), and break-down of relative ligation by each VEGF and PlGF isoform (**H-I**) with varying sR1 production.

To test the ‘ligand-shifting hypothesis,’ i.e. that PlGF induces pro-angiogenic effects *in vivo* by shifting VEGF binding from VEGFR1 to VEGFR2, we altered the amount of PlGF production in tissue, and quantified the resulting changes in cell surface VEGFR1 ligation and total VEGFR2 phosphorylation. To control for changes in cell surface VEGFR1 and total VEGFR2, we normalized these quantities by the relevant receptor population. We found, across a wide range of PlGF production (from zero to 10x baseline levels), that despite large changes in free PlGF levels in tissue (Fig 6A), only modest changes in VEGFR2 ligation and phosphorylation (pR2/R2) were observed (Fig 6B). Conversely, VEGFR1 ligation changes much more (varying from 69% to 389% of baseline VEGFR1 ligation) with PlGF levels. The shift in VEGFR1 ligation is almost entirely due to PlGF; VEGFR1 ligation by VEGF remains approximately constant (Fig 6C). These results suggest that, while at supraphysiologic concentrations (>10x baseline), PlGF may increase VEGFR2 phosphorylation, PlGF and VEGF do not compete for VEGFR1 binding in physiological conditions. This is consistent with the low predicted receptor occupancies, and our previous *in vitro* simulations [45, 46], but is demonstrated here for the first time for *in vivo* scenarios.

#### VEGFR1 ligation is more sensitive than VEGFR2 ligation to matrix site density

While the model predicts that less than 20% of ligated endothelial cell surface receptors are bound to immobilized ligand, the total number of accessible binding site in the endothelial BM is not well-characterized, nor is the fraction of the basement membrane accessible to EC surface receptors. Thus, we examined whether, if growth factor binding sites in the endothelial BM are present at higher or lower density than estimated, a difference in cell surface receptor ligation would be predicted. As we increased the density of accessible sites from baseline levels by factors of 10 and 100, the fraction of cell surface ligated VEGFR2 bound to immobilized VEGF increased, reaching 48% (compared to 6.9% at baseline) with a 100-fold increase in binding site density (Fig 6F). Interestingly, the fraction of ligated cell surface VEGFR1 bound to immobilized ligand (largely PlGF2) increases more quickly with endothelial BM site density, reaching 76% with 10x, and 97% with 100x, compared to 17% at baseline. These results suggest that immobilized ligand-receptor complexes may be important *in vivo* (Fig 6F).

#### sR1 alters the magnitude of receptor activation more than the profile of receptor-bound ligands

Since plasma sR1 levels are known to change in disease, we examined the extent to which sR1 can act in an anti-angiogenic manner to modulate endothelial VEGFR1 and VEGFR2 activation. To do this, we simulated knockdown or overexpression of sR1. As expected, free tissue VEGF and PlGF and ligation of both VEGFR1 and VEGFR2 increases (1.9- and 1.5-fold increases in ligation, respectively) with complete sR1 knockout (Fig 6G). Similarly, overexpression of sR1 reduces EC receptor ligation substantially, but does not completely block binding. Interestingly, the effect is more pronounced on VEGFR1 than VEGFR2, shifting the overall balance of signaling by VEGFR1 vs. VEGFR2 (Fig 6G). We examined whether sR1 perturbation would affect the profiles of ligands bound to VEGFR1 and VEGFR2 (Fig 6H and 6I). We observed little change in the ligand bound to VEGFR2. Changes to VEGFR1 ligation are larger, with relative PlGF binding increasing and relative VEGF_121_ binding decreasing with increasing sR1 production.

#### Immobilized ligand binding to sR1 regulates ligand distribution, binding to EC receptors regulates EC signaling

**Fig 7:**
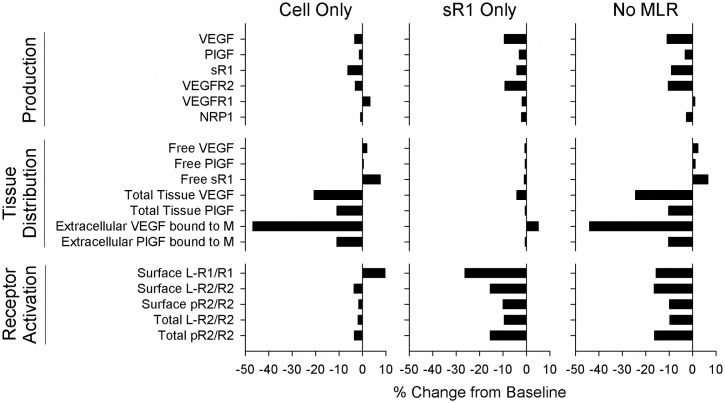
Immobilized ligand binding to sR1 alters tissue distribution, while immobilized ligand binding to EC receptors alters activation state. Panels show percent change from baseline. Thus, the smallest bars indicate little impact of the removed reactions on a given output, while large bars indicate large change when the reactions are removed. **Cell Only:** Immobilized ligand allowed to bind to EC receptors, but not sR1. Binding of ligand to immobilized sR1 is also not allowed. **sR1 Only:** Immobilized ligand allowed to bind to sR1, and ligand to immobilized sR1, but binding of immobilized ligand to EC receptors is not included. **No MLR:** No matrix-ligand-receptor or matrix-ligand-sR1 complexes are allowed to form. **Top:** Changes in fit ligand secretion and receptor production rates to match plasma ligand and sR1 targets and tissue EC surface receptor targets. **Middle:** Distribution of free, total, and matrix-bound VEGF and PlGF. **Bottom:** EC receptor activation.

Next, we examined the relative contribution of immobilized complexes containing sR1 versus EC receptors to our observed results. We compared four cases: (1) the baseline case where 3-element complexes of matrix, VEGF or PlGF, and either sR1 or EC VEGFR1 and VEGFR2 were allowed to form, (2) a case excluding all such reactions (No MLR), (3) a case allowing these reactions on sR1 but not EC receptors (sR1 Only), and (4) a case allowing these reactions on EC receptors but not sR1 (Cell Only). For each case, we re-fit the secretion and production rates to hit our plasma and cell surface receptor targets (S12 Table). We found that sR1 binding to immobilized ligands has a large impact on the amounts of free and total growth factor in tissue (Fig 7). Conversely, EC receptor binding to immobilized ligand increases receptor ligation and phosphorylation. Combined, these effects produce the observed differences between the baseline and No MLR cases.

### Model predictions of signaling in human body with expression of only single VEGF isoforms are consistent with observed murine vascular phenotypes

**Fig 8:**
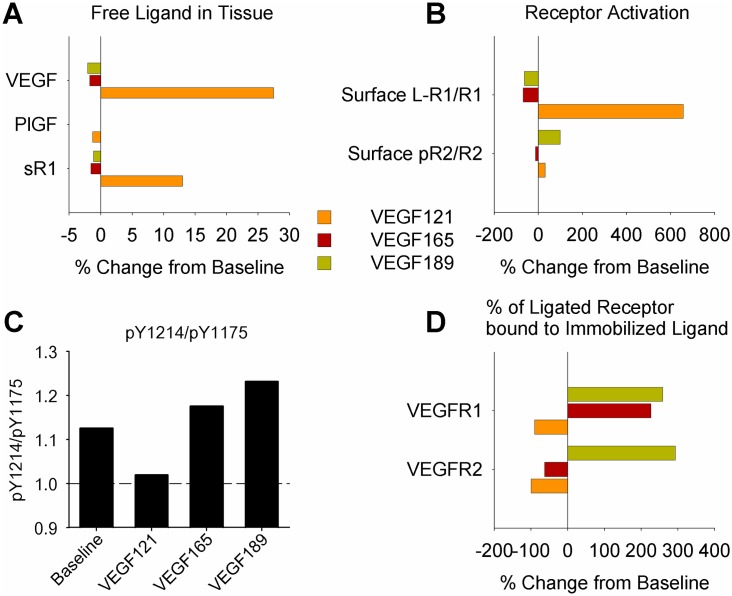
Predicted signaling changes in the human body with expression of single VEGF isoforms mirror experimentally observed murine phenotypes. **(A)** Levels of free VEGF, PlGF, and sR1 in tissue interstitial fluid, normalized to baseline, when all VEGF production is VEGF_121_, VEGF_165_, or VEGF_189_. **(B)** Endothelial cell surface ligation of VEGFR1 and phosphorylation of VEGFR2. Changes in pR2 and ligated VEGFR2 were very similar. **(C)** Ratio of total VEGFR2 phosphorylation on tyrosine Y1214 to phosphorylation of tyrosine Y1175. **(D)** Percent of ligated EC surface VEGFR1 and VEGFR2 bound to EBM-immobilized ligand.

The most convincing evidence to date of differential signaling by VEGF isoforms is the distinct vascular phenotypes of mice or human tumors (implanted in mice) expressing only single isoforms of VEGF, with VEGF_121_-only tissues producing high diameter, sparsely branched networks, VEGF_165_-only tissue a relatively normal phenotype, and VEGF_189_-only tissues networks of thin, highly branched vessels. Endothelial cells isolated from these single isoform-expressing mice also display distinct signaling and behavior in cell culture [81]. It is assumed that similar regulation occurs in humans. To better understand VEGF isoform-specific signaling in the context of the human, as well as to qualitatively validate our model, we simulated expression of a single VEGF isoform in the human body. While no significant changes in VEGFR1 or VEGFR2 mRNA were observed in the muscle of mice expressing only VEGF_120_ [82] (equivalent to human VEGF_121_), we re-fit our model for each case, in order to maintain target ligand and receptor levels (S13 Table). The need for these changes in receptor production and ligand secretion rates may be a result of differences between humans and mice, or underlying compensation mechanisms and physiological changes in the engineered mice [82] not included in this model. Consistent with observations in mice, ligand distribution and VEGFR2 activation are more similar to wild type (baseline) in the VEGF_165_-only than the VEGF_121_-only or VEGF_189_-only cases (Fig 8A and 8B). Similar to the baseline case (Fig 5), where all three isoforms are expressed, with single VEGF isoform expression the ratio of migratory to proliferative signaling downstream of VEGFR2 (pY1214/pY1175) is predicted to increase with isoform length, paralleling the observed phenotypes (Fig 8C). The model’s ability to capture this trend provides qualitative validation of our isoform-specific signaling predictions *in vivo*. Interestingly, the model also predicts other changes, in free VEGF levels in tissue interstitium (Fig 8A) and in relative activation of VEGFR1 and VEGFR2 (Fig 8B and 8D, S1 File).

## Discussion

**Fig 9:**
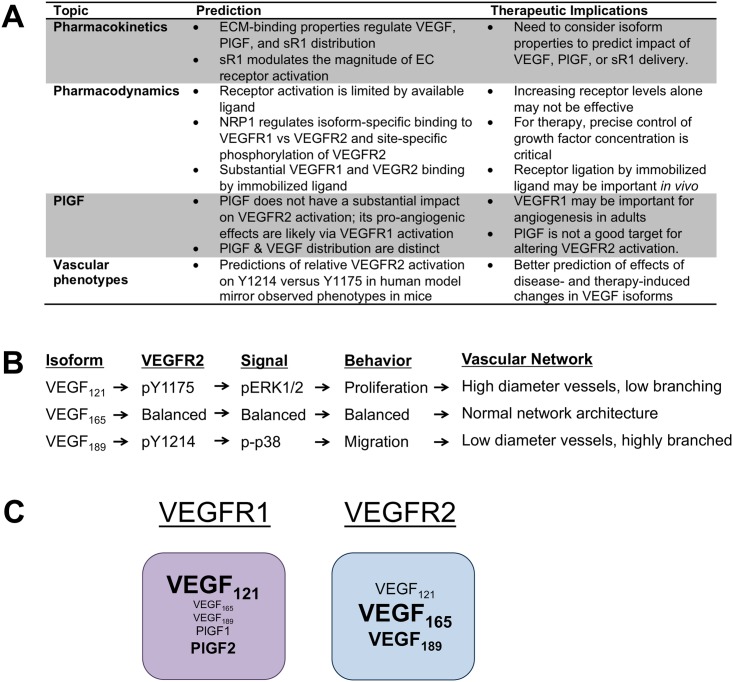
Summary of key model predictions. **(A)** Overview of key predictions. **(B)** Due to differences in NRP1- and ECM-binding, VEGF isoform-VEGFR2 complexes are trafficked differently, leading to distinct downstream signaling, cellular behavior, and vascular network architecture. **(C)** Summary of predicted ligand binding to VEGFR1 and VEGFR2. All ligands in the respective boxes can bind to VEGFR1 or VEGFR2. The size of the ligands represents the predicted contribution to receptor binding *in vivo*. The model suggests that, for each receptor, a subset of the ligands dominate.

We constructed this computational systems pharmacology model to probe the complexity of VEGF family distribution and signaling in the body, for the first time accounting for the impact of PlGF and of receptor binding by basement membrane-immobilized ligands. In demonstrating the contribution of multiple specific mechanisms to regulation of VEGF family signaling, this model explores the sometimes non-intuitive effects these complex interactions have on VEGFR1 and VEGFR2 activation. This model is based on previously-developed compartment models, leveraging the same structure and geometric parameterization. Despite this commonality, adding to and improving the molecular-level detail resulted in changes to some model predictions, as well as the ability to predict VEGFR2 signaling in more detail than was previously possible (see Fig 9A).

### Model provides novel insight into PlGF transport and potential for VEGFR1-dependent PlGF signaling

Our model predicts that, based on their binding properties and *in vivo* concentrations, PlGF and VEGF have distinct distributions within the body. PlGF2, binding to the ECM more strongly than VEGF, is bound to interstitial matrix sites at very high levels (~1 nM in tissue: soluble + ECM-bound + EC-bound predicted, Fig 3C), forming a large reservoir available for proteolytic release. Despite high tissue PlGF levels, our simulations predict that only about 30% of ligated EC surface VEGFR1 is bound to PlGF. As a result, while most VEGF removal from tissue is predicted to occur via binding to endothelial receptors, only 25% of PlGF was predicted to bind to and be subsequently degraded by endothelial cells. PlGF also binds VEGFR1 on other cells, e.g. monocytes and macrophages, that are implicated in arteriogenesis [26, 83]. We found that removing PlGF or increasing PlGF secretion has only a modest effect on predicted VEGFR2 phosphorylation, while substantially altering VEGFR1 activation (Fig 6A). This result suggests that observed physiological PlGF-dependent pro-angiogenic effects are likely mediated directly by VEGFR1, either on ECs or other cells, and not via changes in VEGFR2 signaling, contrary to the ‘ligand-shifting hypothesis’. This result implicates VEGFR1 in the impaired angiogenic responses to ischemia, wound healing, and cancer [21] observed in mice lacking PlGF. It also implicates VEGFR1 in diseases where PlGF levels are known to change or to be predictive of prognosis, e.g. pre-eclampsia [42] and breast cancer [84]. The pro-angiogenic effects of PlGF likely also rely on its ability to up-regulate other growth factors, including VEGF, FGF2, and PDGF [85, 86].

This result is not inconsistent with recent work by the Alitalo group showing that therapeutic over-expression of VEGFB (which like PlGF binds only VEGFR1) in mice improves metabolic health even following endothelial Flt1 gene deletion, and inhibits doxorubicin-induced cardiotoxicity [54, 87]. Competition between ligands is concentration-dependent, and in these studies, VEGFB protein levels were elevated 20-fold or more in serum, heart, liver, and white adipose tissue. Our model predicts that competition is not a driver of PlGF signaling in physiological conditions, but does not preclude the existence of competition following supraphysiologic therapy. Indeed, at 10-fold PlGF over-expression, outside of the concentration range likely to be observed in untreated healthy or diseased tissue [42], the model does begin to predict an effect on VEGFR2 signaling.

### Growth factor immobilization and binding to soluble VEGFR1 predicted to be important for VEGF family signaling *in vivo*

Both the ECM and sR1 regulate tissue levels of free interstitial VEGF and PlGF, the amount of growth factor available to bind ECs, and the steady-state distribution of ligand throughout the body (Fig 3). The model predicts that sR1 modulates the magnitude of EC receptor ligation, potentially also altering the balance of signaling via VEGFR1 vs. VEGFR2 (Fig 6G). This is of therapeutic interest because ratios of VEGF or PlGF to sR1 levels in plasma are increasingly of interest as a biomarker (e.g. in pre-eclampsia) [70], and sR1 levels increase in diabetic mice following hindlimb ischemia [88]. Including binding of immobilized ligands to sR1, and binding of immobilized sR1 to VEGF_121_ and PlGF1, increases total extracellular VEGF and PlGF stored in tissue (Fig 7). While there is not yet evidence to prove the existence of such complexes, the heparin- and ligand-binding sites on sR1 are distinct, as are the heparin- and receptor-binding domains on VEGF and PlGF, and therefore these complexes are likely.

Unlike matrix-ligand-sR1 complexes, VEGF immobilized to both surfaces and ECM proteins has been shown to bind and activate VEGFR2 *in vitro*, preferentially increasing VEGFR2 activation of tyrosine Y1214, upstream of p38 phosphorylation and migratory cell behavior, demonstrating an important role for physical immobilization of VEGF in signal regulation *in vitro* [18, 19, 89]. However, whether VEGFR2 ligation by immobilized VEGF would occur to any notable extent *in vivo*, and what the physiological impact on EC receptor signaling would be, have been unknown. Here, we saw that including these reactions increased EC receptor ligation and altered VEGFR2 signaling (Fig 7). While the number of available sites in the EBM is not well-established, our model suggests that these M-L-R complexes may make up a small but significant portion of ligated EC receptors (Fig 4D). To improve our estimates of the extent of EC receptor ligand by EBM-bound growth factor, it is necessary to obtain better estimates of heparin-binding sites in basement membranes. Interestingly, the fraction of ligated VEGFR1 bound to immobilized ligand was predicted to be higher than that for VEGFR2, owing largely to the strong M-PlGF2 affinity (Fig 6F). To date, the impact of VEGFR1 ligation by immobilized ligand has not been studied. However, as these are largely PlGF2-VEGFR1 complexes (Fig 6F), EBM binding site density may shift relative ligation of VEGFR1 by VEGF versus PlGF, which is known to alter VEGFR1-mediated signaling [44]. Spatial patterning of receptor ligation by soluble and immobilized ligand is also likely to be important, but cannot be examined with this model. Additionally, the potential roles for HSPGs and NRP1 expressed on other cells engaging with VEGFR2 *in trans* [90, 91] are of interest for future study.

### Model predicts VEGF isoform-specific activation of VEGFR1 and VEGFR2

We were interested in differences in signaling between VEGF isoforms upon binding to VEGFR1 and VEGFR2. Explicitly simulating VEGFR2 trafficking and site-specific phosphorylation, placed in the context of physiological geometry and transport processes, allowed us to predict isoform-specific VEGFR2 signaling *in vivo* (Fig 5). Immobilization in the matrix alters VEGF distribution and the resulting signaling, while NRP1 alters VEGF-receptor binding and trafficking. By including these isoform-specific properties, the model predicts that VEGF_121_ induces a shift in VEGFR2 distribution towards early signaling endosomes, decreasing the signaling ratio pY1214/pY1175, and shifting the net cellular signaling towards proliferation. Conversely, a larger portion of VEGFR2 bound to VEGF_189_ was localized on the EC surface at steady-state, increasing pY1214/pY1175, and shifting the balance towards pro-migratory signaling (Fig 5C). This isoform-specific patterning in VEGFR2 signaling was seen in both the baseline case (Fig 5C), with all three VEGF isoforms present, and in cases where only single isoforms of VEGF were expressed (Fig 8C). This is key validation, as our simulated signaling predictions in humans match the observed vascular phenotypes in mice or tumors expressing single VEGF isoforms (Fig 9B). Interestingly, in the single isoform cases, change in relative activation of VEGFR1 and VEGFR2 were also predicted (Fig 8B), which may contribute to these phenotypes [92, 93].

This is in line with another interesting model prediction; while all VEGF isoforms can bind to both VEGFR1 and VEGFR2, physiologically it appears that VEGF_165_ and VEGF_189_ bind almost exclusively to VEGFR2, while VEGF_121_ comprises a large portion of the ligand on VEGFR1, and also binds VEGFR2 to an extent (Fig 4D). This segregation of ligands suggests that, while ligand levels are limiting for receptor binding, VEGFR1 and VEGFR2 don’t directly compete for VEGF *in vivo*, instead binding to largely distinct subsets of ligands dictated primarily by isoform-specific NRP1-binding properties (Fig 9C). The relative levels of VEGF isoforms are not yet extensively-characterized, but they are known to vary by tissue and to change in disease [69, 82, 94, 95]. As such, this model can be used to understand splicing-induced tissue- and disease-specific changes in VEGF receptor signaling.

### Considerations for interpretation of model predictions

Our model is built upon experimental data and a validated model of VEGFR2 signaling *in vitro*, and provides new insight into distribution of and signaling by VEGF and PlGF isoforms *in vivo*. However, when interpreting the results, it is important to acknowledge mismatch between model predictions and experimental measurements, which may result from limitations of our modeling approach, uncertainly in interpretation of experimental measures, and/or missing understanding of underlying biological mechanism. Similar to previous models, our predicted interstitial VEGF concentrations when fitting the model to measured plasma VEGF levels are higher than those measured in tissues using microdialysis. This discrepancy could be due to: difficulty in obtaining accurate measurements for high molecular weight proteins using microdialysis; production of VEGF by blood sources (e.g. PBMCs, platelets) or specific organs (e.g. highly fenestrated tissue), reducing the requisite VEGF production by skeletal muscle; or degradation of VEGF by tissue-resident proteases and/or other cell types expressing VEGF receptors (modeled in [96, 97]). Inclusion of proteases in the model would reduce immobilized growth factor stores at steady state. Additionally, as in previous models, the predicted fraction of plasma sR1 bound to ligand was higher than the experimentally-measured fraction. There are other soluble receptors that may be important to consider and are not included here. There may also be limitations with the experimental method that make these *in vivo* measurements inaccurate. To quantify the importance of some difficult-to-measure parameters, as well as reactions included in this model for the first time (some of which have not been explicitly demonstrated experimentally), we analyzed the sensitivity of many new or poorly characterized parameters (see S3 Fig and S1 File).

In order to achieve simulation at the whole body scale, compartment models neglect spatial effects, instead predicting only average values for tissue. The interstitial space of the tissue, the cell surface of endothelial cells and the cell surface of myocytes are still independent entities in this case and each is treated as well-mixed. Detailed study of gradients in interstitial space and along cell surfaces, which are difficult to measure *in vivo* but are likely key to angiogenic signaling, requires development of detailed 2- and 3-dimensional models of tissue and experimental set-ups, calibrated to match predicted average concentrations from compartment models [98–101] such as the one presented here. Much work remains to fully understand the role of spatial gradients of VEGF distribution and receptor activation in health, disease, and response to therapy.

### Conclusions

This model integrates detailed regulation of VEGF and PlGF distribution and binding to EC VEGFR1 and VEGFR2 by sR1, the ECM, and NRP1 into a multi-scale pharmacokinetic/pharmacodynamic (PK/PD) framework. The resulting model predicts that all of these features interact, and contribute to regulation of tissue-level VEGF family signaling. While many model predictions are difficult to validate *in vivo*, the mechanisms included were first modeled using detailed *in vitro* measurements, and validated in many cases on the cellular level, before being put in a physiological context using an existing PK/PD framework. By progressively adding complexity, we can study the impact of each contribution, and compare simulation results to quantities that are measurable and to observable phenotypes, such as the vascular morphologies in mice expressing single isoforms of VEGF. By the same turn, this model provides a window into details of growth factor distribution and signaling that are essentially impossible to measure (especially on the protein level), though in many cases implicated in disease-related impairment in angiogenic response, or targeted by potential therapies. The lack of approved pro-angiogenic therapies to date makes it clear that a better understanding of the molecular mechanisms driving disease is critical to identify more effective drug targets, optimize drug properties (e.g. affinity), and avoid off-target effects leading to toxicity and drug failure [55]. This work can be extended to disease applications with changes in VEGF splicing, and to compare results in humans versus mice, to aid in translation of therapeutics targeting the VEGF system and to further validate the model against data obtained in mice.

## Supporting information

S1 File Supplemental results. (DOCX)

S1 Equations Supplemental equations. (DOCX)

S1 Fig Super-sensitivity of steady-state VEGF and VEGFR2 levels, compared to previous model set-up. These panels expands upon the results shown in Fig 2 of the main manuscript. **(A)** In previous models, surface VEGFR2 levels were fixed (same internalization rate for free and VEGF-bound VEGFR2, no recycling), so increasing VEGF levels would lead to more VEGF-VEGFR2 binding and subsequent degradation of VEGF, keeping the net change in VEGF levels relatively small. **(B)** In this model, trafficking rates are different for free and ligand-bound VEGFR2, so endothelial cell surface VEGFR2 levels are not constant when VEGF levels change. If VEGF levels increase, more VEGFR2 becomes occupied, internalized, and degraded, reducing steady-state VEGFR2 levels and decreasing VEGF consumption via VEGFR2 (purple). Similarly, if VEGFR2 production increases, more VEGF is bound to VEGFR2, internalized, and degraded, reducing steady-state VEGF levels and as a result further increasing surface VEGFR2 (green).
(TIF)

S2 Fig Additional pharmacokinetic/pharmacodynamic predictions of the model. **(A)** This panel, which shows “local” concentrations of growth factor accessible to endothelial cell receptors, is related to Fig 4A of the main manuscript. EBM-bound growth factor concentrations are calculated using the EBM volume, while free levels are calculated using the total available interstitial space. **(B)** This panel expands upon the results shown in Fig 5 of the main manuscript. For each isoform, total phosphorylated VEGFR2 (pR2) bound to the given ligand is divided by total VEGFR2 bound to the respective ligand.
(TIF)

S3 Fig Sensitivity of transport parameters and new or unconfirmed reactions. **(A)** Sensitivity of ligand distribution and receptor activation to changes in, from left to right: NRP1 production rate (sN1), vascular permeability (k_p_), lymphatic drainage rate (k_L_), and rate of clearance from the blood (k_CL_). All tissue quantities are taken from the “Main Body Mass” compartment. Values shown are the average magnitude of change in a given quantity when the specified parameter is increased or decreased by a factor of 10 (baseline = 0). Note the different scale on the NRP1 production rate than on the other panels. **(B)** Changes to ligand distribution and receptor activation when k_on_ for different reactions is set to zero, prohibiting the selected reactions from occurring. Values shown are fold change from baseline (baseline = 1). Examined reactions are, from left to right: binding of sR1 to EC NRP1 (with or without ligand), binding of ligand to sR1-N1 complexes, binding of PlGF1 to NRP1-VEGFR1 and NRP1-sR1 complexes, formation of immobilized ligand-VEGFR1 and immobilized ligand-sR1 complexes (in any form), binding of VEGF_121_ or PlGF1 to immobilized sR1, binding of free sR1 to matrix proteins (no ligand), binding of immobilized ligands to sR1 (only), and binding of matrix proteins to VEGF_165_, VEGF_189_, or PlGF2 bound to sR1. All tissue quantities taken from “Main Body Mass” compartment. Note the different scale on the sR1-N1 and P1-(N1-R1) panels than on the other panels.
(TIF)

S1 Table Binding/Unbinding reactions: K_D_ in the main body mass. (DOCX)

S2 Table Binding/Unbinding reactions: K_D_ in healthy calf muscle. (DOCX)

S3 Table Binding/Unbinding reactions: K_D_ in plasma. (DOCX)

S4 Table Binding/Unbinding reactions: k_on_ in the main body mass. (DOCX)

S5 Table Binding/Unbinding reactions: k_on_ in healthy calf muscle. (DOCX)

S6 Table Binding/Unbinding reactions: k_on_ in plasma. (DOCX)

S7 Table Geometric parameterization. (DOCX)

S8 Table Trafficking parameters. (DOCX)

S9 Table Phosphorylation parameters. (DOCX)

S10 Table Transport parameters. (DOCX)

S11 Table Available matrix site densities. (DOCX)

S12 Table Production and secretion rates for “MLR” cases (Fig 7). (DOCX)

S13 Table Production and secretion rates for single VEGF isoform cases (Fig 8). (DOCX)
