# Supplementary material for: A computational analysis of in vivo VEGFR activation by multiple co-expressed ligands
Source: PLoS Comput Biol. 2017 Mar 20;13(3):e1005445. doi: 10.1371/journal.pcbi.1005445 (PMC5378411; doi:10.1371/journal.pcbi.1005445)
Supplement: S1 File — (DOCX) [file pcbi.1005445.s001.docx]

# Supplemental Results

## Pharmacokinetics & Pharmacodynamics

### High immobilized growth factor concentrations in endothelial basement membrane. When the concentration of endothelial basement membrane immobilized growth factor is calculated using the volume of the basement membrane (instead of the total available interstitial space, as the concentration of free ligand is calculated), the concentration of BME-bound ligand is higher than the concentration of free ligand “seen” by endothelial cells for all growth factors (S2A Figure). While VEGF_121_ and PlGF1 are only bound to the EBM in complex with sR1, the local “available” (non-sR1-bound) VEGF_165_, VEGF_189_, and PlGF2 concentrations bound to the EBM are in the nanomolar range, far above the picomolar-range concentrations of free VEGF and PlGF in interstitial space. While spatial effects are not taken into account in this compartment model, this may have important implications for relative receptor ligation by free and immobilized growth factor, as well as spatial patterning due to ECM and basement membrane heterogeneity in vivo.

### Fractional activation of ligated VEGFR2 is predicted to be VEGF isoform-specific. Our model predicts that the dephosphorylation rates are higher on the cell surface than in early endosomes, and very high in the NRP1-dependent Rab11 recycling pathway, altering the total amount of phosphorylated VEGFR2 in an isoform-specific manner (89.6% of total VEGF_121_-R2, 58.0% of total VEGF_165_-R2, 61.4% of total VEGF_189_-R2) (S2B Figure). This novel prediction, suggesting varying “potency” of different VEGF isoforms, is a direct consequence of isoform-specific NRP1- and matrix-binding leading to isoform-specific trafficking. The relative contributions of NRP1-binding and matrix-binding to isoform-specific signaling could be validated experimentally using VEGF isoforms engineered to bind ECM but not NRP1 (e.g.[1]) Note that, while a large quantity of ligated VEGFR2 is present in Rab11 recycling endosomes at steady-state, very little of this VEGFR2 pool is phosphorylated (Figures 4 & 5).

## Single VEGF Isoform Expression

Interestingly, the model predicts changes in ligand distribution and receptor activation in our single isoform simulations, compared to the baseline case. Specifically, after fitting to maintain the same plasma VEGF levels as baseline, expression of only VEGF_121_ is predicted to lead to higher tissue levels of free VEGF (**Figure 8A**) than expression of only heparin-binding VEGF isoforms (**Figure 8B**). This leads to increased receptor ligation in the VEGF_121_ only case, while VEGF_189_ binds strongly to the ECM, increasing the proportion of surface M-V-R2 complexes substantially (27.1%, compared to 6.9% in baseline case) (**Figure 8D**), and retaining VEGFR2 at the cell surface longer. As a result, expression of only VEGF_189_ leads to higher predicted cell surface pR2/R2 than VEGF_121_ alone, while VEGF_165_ alone leads to slightly lower-than-baseline pR2/R2 (**Figure 8B**). This is consistent with our baseline observations; when normalized by the fraction of VEGF production for each isoform, VEGF_121_ is over-represented in VEGFR1 ligation, and both VEGF_121_ and VEGF_189_ are over-represented in VEGFR2 ligation (see **Figure 3E**). In summary, our model predicts that, compared to baseline, expression of only VEGF­_189_ increases both pY1214/pY1175 and the relative activation of VEGFR2 compared to VEGFR1, while expression of VEGF_121_ alone decreases these quantities. VEGFR1 loss or blockade results in a hyperproliferative vascular phenotype [2, 3], suggesting that the balance of VEGFR1 and VEGFR2 activation may contribute to the observed VEGF isoform-specific phenotypes.

## Sensitivity of transport parameters and new reactions included in the model

We analyzed the sensitivity of our model to a few additional parameters whose values are not well-established, as well as a few reactions that have not been clearly demonstrated to occur. We started with NRP1 production, as NRP1 is clearly an important regulator of VEGF-family signaling, as well as the rates for bidirectional vascular permeability, lymphatic drainage, and clearance of growth factors and sR1 from the blood (**Figure S3A**). We increased, then decreased, each rate by a factor of two, and averaged the change from baseline. As anticipated, both ligand distribution and receptor activation were quite sensitive to NRP1 production. The system was less sensitive to changes in transport parameters; perturbations of vascular permeability (k_p_) and lymphatic drainage (k_L_) resulted in similar changes in ligand distribution and VEGFR1 ligation. Clearance from the blood (k_CL_) led to large changes only in plasma protein levels.

Next, we examined the effect of removing reactions that have not been proven to occur from the model (**Figure S3B**). We calculated the fold changes in outputs from baseline when a given reaction was removed. Removing binding of sR1 to EC surface NRP1 (to form non-signaling complexes) had a large effect on ligand distribution, as this is a key route of sR1 clearance, and (as a result) also affected receptor activation. Disallowing ligand-binding to sR1-NRP1 complexes had a smaller effect. As the binding properties of PlGF are less well-studied than those of VEGF, we assumed, similar to VEGF, that the shorter PlGF1 can bind to NRP1-VEGFR1 complexes, while PlGF2 cannot. If we remove binding of PlGF1 to NRP1-VEGFR1 complexes, we saw little change aside from the amount of PlGF1 binding to VEGFR1. While immobilized ligand has been shown to bind and activate VEGFR2, the same has not been proven for VEGFR1, or sR1. As such, we examined the effect of allowing these complexes to form with VEGFR2, but not with VEGFR1 or sR1. As expected, we saw large changes in VEGFR1 ligation. While all matrix-ligand-sR1 complexes were lost, there was relatively little change in the amount of ligand bound to matrix alone. As we found earlier that formation of immobilized ligand-sR1 complexes regulates ligand distribution, we looked to see if any of the particular paths to form these complexes were especially sensitive. The sensitivity for removal of any single path was relatively low; the maximum change to the concentration of matrix-ligand-sR1 complexes was 17%. This suggests that the combination of these routes, not a single path, contributes to formation of immobilized ligand-sR1 complexes and regulation of ligand distribution. Note the log scale in **Figure S3B** when comparing these results to **Figure 7**.

**Supplemental References**

1. Martino MM, Briquez PS, Güç E, Tortelli F, Kilarski WW, Metzger S, et al. Growth Factors Engineered for Super-Affinity to the Extracellular Matrix Enhance Tissue Healing. Science. 2014;343(6173):885-8. doi: 10.1126/science.1247663.

2. Fong GH, Rossant J, Gertsenstein M, Breitman ML. Role of the Flt-1 receptor tyrosine kinase in regulating the assembly of vascular endothelium. Nature. 1995;376(6535):66-70. doi: 10.1038/376066a0. PubMed PMID: WOS:A1995RH11100063.

3. Bussolati B, Dunk C, Grohman M, Kontos CD, Mason J, Ahmed A. Vascular endothelial growth factor receptor-1 modulates vascular endothelial growth factor-mediated angiogenesis via nitric oxide. American Journal of Pathology. 2001;159(3):993-1008. doi: 10.1016/s0002-9440(10)61775-0. PubMed PMID: WOS:000170872400026.
