# Supplementary material for: A computational analysis of in vivo VEGFR activation by multiple co-expressed ligands
Source: PLoS Comput Biol. 2017 Mar 20;13(3):e1005445. doi: 10.1371/journal.pcbi.1005445 (PMC5378411; doi:10.1371/journal.pcbi.1005445)
Supplement: S1 Equations — (DOCX) [file pcbi.1005445.s002.docx]

# Supplemental Equations

## Unit Conversions

Due to the multi-compartment structure of the model, unit conversions are required to account for differences in compartment volumes (U), as well as reactions that occur in solution or on the two-dimensional cell surface. In the equations, concentrations are represented by [X]_j­_, where j represents the tissue compartment (calf muscle or main body mass) or [X]_B_, where B is the blood. The units of concentration are moles/cm^3^ of tissue or blood, as appropriate. In many cases (e.g. for transport), the relevant concentration is instead the concentration of ligand in available interstitial fluid (excluding spaces that are inaccessible to proteins), annotated [X]_IS,j_ or the concentration of ligand in plasma, annotated [X]_pl­_. In the text and figures, the concentration being discussed is identified explicitly. Many outputs are shown in picomolar (pM), nanomolar (nM), or total picomoles of ligand in tissue, using simple unit conversions.

To convert between concentrations in total tissue and in interstitial fluid, the following equations are used:

$[X]_{IS,j}=\frac{[X]_{j}}{K_{AV,j}}$ , where **$K_{AV,j}=\frac{U_{IS,j}}{U_{j}}=\frac{\text{available interstitial volume}}{\text{total volume of tissue j}}$**

$$K_{AV,j}=\text{Available Volume Fraction }=\Phi\cdot f\cdot\varepsilon_{IS}$$

$$\Phi=\text{Partition Coefficient}=\frac{\text{available fluid volume}}{\text{interstitial fluid volume}}$$

$$f=\text{Fluid Fraction}=\frac{\text{interstitial fluid volume}}{\text{interstitial space}}$$

$$\varepsilon_{IS}=\text{Interstitial Fraction}=\frac{\text{interstitial space}}{\text{total tissue volume}}$$

Similarly, the conversion between total blood and plasma concentrations is:

$[X]_{pl}=\frac{[X]_{B}}{K_{AV,B}}$ **,** where **$K_{AV,B}=\frac{U_{pl}}{U_{B}}=\frac{\text{plasma volume}}{\text{total blood volume}}$**

To convert between measured receptor levels of number per cell to moles/cm^3^ tissue:

$[R]\text{ in }\frac{\text{moles}}{\text{cm}^{3}\text{ tissue}}=\left([R]\text{ in }\frac{\#}{\text{EC}}\right)\cdot\frac{\text{ESAV}}{\text{ECSA}}\cdot\frac{1}{\text{N}_{\text{Av}}}$ , where N_Av_ is Avogadro’s number, 6.023x10^23^ molecules/mole.

## Tissue Equations

This section lists the 112 equations that describe all reactions (excluding different VEGFR2 phospho-states) and transport within the tissue compartments.

Interstitial Matrix. These 30 equations describe the HSPGs site (M) in the ECM, endothelial basement membrane (EBM), and parenchymal basement membrane (PBM), either free, or bound to VEGF, PlGF, sR1, or sR1 and VEGF or PlGF. EBM-bound VEGF in the innermost 25nm of the EBM can bind to endothelial cell-surface VEGFR1, VEGFR2, and NRP1. We assume that, similar to cell-surface receptors, matrix-binding VEGF and PlGF isoforms (VEGF_165­_, VEGF_189_, and PlGF), can bind to HSPGs sites and sR1 simultaneously, forming M-L-sR1 complexes, where L represents the ligand (either VEGF or PlGF). Since VEGFR1 can bind to NRP1 and VEGF_121_ or PlGF1 simultaneously, and the NRP1 and matrix-binding sites on VEGFR1 overlap, we assume that these ligands can also bind to immobilized sR1, forming L-sR1-M complexes. Thus, complexes including VEGF or PlGF, matrix, and sR1 can form for all ligands, but in different ways (see **Figure 1B**). To reflect this, the ordering of species in the complexes described within the equations are ordered to show the actual binding partners next to each other. [M_ECM_]_j­_ represents the concentration of free HSPG sites in the ECM, [V_165_] the interstitial concentration of free VEGF_165_, [P1] the interstitial concentration of free PlGF1, [sR1] the interstitial concentration of free soluble VEGFR, and [R1] the concentration of unoccupied EC surface VEGFR1. The binding rates (k_on_) and unbinding rates (k_off­_) are given in **Tables 2 & 3**, with units of M^-1^s^-1^ and s^-1^, respectively. To convert the binding rates (k_on_) into an *in vivo* context (moles/cm^3^ tissue/s for each compartment), the following conversions were applied, using geometric parameters from **Table S7**, as previously described[1, 2].

$$k_{on}\text{ in }\frac{\text{moles}}{\text{cm}^{3}\text{ tissue}}^{-1}\cdot s^{-1}=k_{on}\text{ in }M^{-1}\cdot s^{-1}\cdot\frac{1000}{\text{available IF} \text{ volume fraction}}$$

$$k_{on}\text{ in }\frac{\text{moles}}{\text{cm}^{3}\text{ tissue}}^{-1}\cdot s^{-1}=k_{on}\text{ in }\left(\frac{moles}{cm^{2}\text{ }SA}\right)^{-1}\cdot s^{-1}\cdot\frac{1}{ESAV}$$

The resulting k_on_ values are given in **Tables S4-S6**.

$$\begin{matrix} d[M_{ECM}]_{j}/dt=-k_{on,V165\cdot M,j}[V_{165}]_{j}[M_{ECM}]_{j}+k_{off,V165\cdot M,j}[V_{165}\cdot M_{ECM}]_{j} \\ -k_{on,V189\cdot M,j}[V_{189}]_{j}[M_{ECM}]_{j}+k_{off,V189\cdot M,j}[V_{189}\cdot M_{ECM}]_{j} \\ -k_{on,P2\cdot M,j}[P2]_{j}[M_{ECM}]_{j}+k_{off,P2\cdot M,j}[P2\cdot M_{ECM}]_{j} \\ -k_{on,sR1\cdot M,j}[sR1]_{j}[M_{ECM}]_{j}+k_{off,sR1\cdot M,j}[sR1\cdot M_{ECM}]_{j} \\ -k_{on,(V121\cdot sR1)\cdot M,j}[V_{121}\cdot sR1]_{j}[M_{ECM}]_{j}+k_{off,(V121\cdot sR1)\cdot M,j}[V_{121}\cdot sR1\cdot M_{ECM}]_{j} \\ -k_{on,(P1\cdot sR1)\cdot M,j}[P1\cdot sR1]_{j}[M_{ECM}]_{j}+k_{off,(P1\cdot sR1)\cdot M,j}[P1\cdot sR1\cdot M_{ECM}]_{j} \\ -k_{on,M\cdot(V165\cdot sR1),j}[M_{ECM}]_{j}[V_{165}\cdot sR1]_{j}+k_{off,M\cdot(V165\cdot sR1),j}[M_{ECM}\cdot V_{165}\cdot sR1]_{j} \\ -k_{on,M\cdot(V189\cdot sR1),j}[M_{ECM}]_{j}[V_{189}\cdot sR1]_{j}+k_{off,M\cdot(V189\cdot sR1),j}[M_{ECM}\cdot V_{189}\cdot sR1]_{j} \end{matrix}$$

$$\begin{matrix} d[M_{PBM}]_{j}/dt=-k_{on,V165\cdot M,j}[V_{165}]_{j}[M_{PBM}]_{j}+k_{off,V165\cdot M,j}[V_{165}\cdot M_{PBM}]_{j} \\ -k_{on,V189\cdot M,j}[V_{189}]_{j}[M_{PBM}]_{j}+k_{off,V189\cdot M,j}[V_{189}\cdot M_{PBM}]_{j} \\ -k_{on,P2\cdot M,j}[P2]_{j}[M_{PBM}]_{j}+k_{off,P2\cdot M,j}[P2\cdot M_{PBM}]_{j} \\ -k_{on,sR1\cdot M,j}[sR1]_{j}[M_{PBM}]_{j}+k_{off,sR1\cdot M,j}[sR1\cdot M_{PBM}]_{j} \\ -k_{on,(V121\cdot sR1)\cdot M,j}[V_{121}\cdot sR1]_{j}[M_{PBM}]_{j}+k_{off,(V121\cdot sR1)\cdot M,j}[V_{121}\cdot sR1\cdot M_{PBM}]_{j} \\ -k_{on,(P1\cdot sR1)\cdot M,j}[P1\cdot sR1]_{j}[M_{PBM}]_{j}+k_{off,(P1\cdot sR1)\cdot M,j}[P1\cdot sR1\cdot M_{PBM}]_{j} \\ -k_{on,M\cdot(V165\cdot sR1),j}[M_{PBM}]_{j}[V_{165}\cdot sR1]_{j}+k_{off,M\cdot(V165\cdot sR1),j}[M_{PBM}\cdot V_{165}\cdot sR1]_{j} \\ -k_{on,M\cdot(V189\cdot sR1),j}[M_{PBM}]_{j}[V_{189}\cdot sR1]_{j}+k_{off,M\cdot(V189\cdot sR1),j}[M_{PBM}\cdot V_{189}\cdot sR1]_{j} \end{matrix}$$

$$\begin{matrix} d[V_{165}\cdot M_{EBM}]_{j}/dt=k_{on,V165\cdot M,j}[V_{165}]_{j}[M_{EBM}]_{j}-k_{off,V165\cdot M,j}[V_{165}\cdot M_{EBM}]_{j} \\ -k_{on,(M\cdot V165)\cdot R2,j}[M_{EBM}\cdot V_{165}]_{j}[R2]_{j}+k_{off,(M\cdot V165)\cdot R2,j}[M_{EBM}\cdot V_{165}\cdot R2]_{j} \\ -k_{on,(M\cdot V165)\cdot R1,j}[M_{EBM}\cdot V_{165}]_{j}[R1]_{j}+k_{off,(M\cdot V165)\cdot R1,j}[M_{EBM}\cdot V_{165}\cdot R1]_{j} \\ -k_{on,(M\cdot V165)\cdot sR1,j}[M_{EBM}\cdot V_{165}]_{j}[sR1]_{j}+k_{off,(M\cdot V165)\cdot sR1,j}[M_{EBM}\cdot V_{165}\cdot sR1]_{j} \end{matrix}$$

$$\begin{matrix} d[V_{189}\cdot M_{EBM}]_{j}/dt=k_{on,V189\cdot M,j}[V_{189}]_{j}[M_{EBM}]_{j}-k_{off,V189\cdot M,j}[V_{189}\cdot M_{EBM}]_{j} \\ -k_{on,(M\cdot V189)\cdot R2,j}[M_{EBM}\cdot V_{189}]_{j}[R2]_{j}+k_{off,(M\cdot V189)\cdot R2,j}[M_{EBM}\cdot V_{189}\cdot R2]_{j} \\ -k_{on,(M\cdot V189)\cdot R1,j}[M_{EBM}\cdot V_{189}]_{j}[R1]_{j}+k_{off,(M\cdot V189)\cdot R1,j}[M_{EBM}\cdot V_{189}\cdot R1]_{j} \\ -k_{on,(M\cdot V189)\cdot sR1,j}[M_{EBM}\cdot V_{189}]_{j}[sR1]_{j}+k_{off,(M\cdot V189)\cdot sR1,j}[M_{EBM}\cdot V_{189}\cdot sR1]_{j} \end{matrix}$$

$$\begin{matrix} d[P2\cdot M_{EBM}]_{j}/dt=k_{on,P2\cdot M,j}[P2]_{j}[M_{EBM}]_{j}-k_{off,P2\cdot M,j}[P2\cdot M_{EBM}]_{j} \\ -k_{on,(M\cdot P2)\cdot R1,j}[M_{EBM}\cdot P2]_{j}[R1]_{j}+k_{off,(M\cdot P2)\cdot R1,j}[M_{EBM}\cdot P2\cdot R1]_{j} \\ -k_{on,(M\cdot P2)\cdot sR1,j}[M_{EBM}\cdot P2]_{j}[sR1]_{j}+k_{off,(M\cdot P2)\cdot sR1,j}[M_{EBM}\cdot P2\cdot sR1]_{j} \end{matrix}$$

$$\begin{matrix} d[V_{165}\cdot M_{ECM}]_{j}/dt=k_{on,V165\cdot M,j}[V_{165}]_{j}[M_{ECM}]_{j}-k_{off,V165\cdot M,j}[V_{165}\cdot M_{ECM}]_{j} \\ -k_{on,(M\cdot V165)\cdot sR1,j}[M_{ECM}\cdot V_{165}]_{j}[sR1]_{j}+k_{off,(M\cdot V165)\cdot sR1,j}[M_{ECM}\cdot V_{165}\cdot sR1]_{j} \end{matrix}$$

$$\begin{matrix} d[V_{189}\cdot M_{ECM}]_{j}/dt=k_{on,V189\cdot M,j}[V_{189}]_{j}[M_{ECM}]_{j}-k_{off,V189\cdot M,j}[V_{189}\cdot M_{ECM}]_{j} \\ -k_{on,(M\cdot V189)\cdot sR1,j}[M_{ECM}\cdot V_{189}]_{j}[sR1]_{j}+k_{off,(M\cdot V189)\cdot sR1,j}[M_{ECM}\cdot V_{189}\cdot sR1]_{j} \end{matrix}$$

$$\begin{matrix} d[P2\cdot M_{ECM}]_{j}/dt=k_{on,P2\cdot M,j}[P2]_{j}[M_{ECM}]_{j}-k_{off,P2\cdot M,j}[P2\cdot M_{ECM}]_{j} \\ -k_{on,(M\cdot P2)\cdot sR1,j}[M_{ECM}\cdot P2]_{j}[sR1]_{j}+k_{off,(M\cdot P2)\cdot sR1,j}[M_{ECM}\cdot P2\cdot sR1]_{j} \end{matrix}$$

$$\begin{matrix} d[V_{165}\cdot M_{PBM}]_{j}/dt=k_{on,V165\cdot M,j}[V_{165}]_{j}[M_{PBM}]_{j}-k_{off,V165\cdot M,j}[V_{165}\cdot M_{PBM}]_{j} \\ -k_{on,(M\cdot V165)\cdot sR1,j}[M_{PBM}\cdot V_{165}]_{j}[sR1]_{j}+k_{off,(M\cdot V165)\cdot sR1,j}[M_{PBM}\cdot V_{165}\cdot sR1]_{j} \end{matrix}$$

$$\begin{matrix} d[V_{189}\cdot M_{PBM}]_{j}/dt=k_{on,V189\cdot M,j}[V_{189}]_{j}[M_{PBM}]_{j}-k_{off,V189\cdot M,j}[V_{189}\cdot M_{PBM}]_{j} \\ -k_{on,(M\cdot V189)\cdot sR1,j}[M_{PBM}\cdot V_{189}]_{j}[sR1]_{j}+k_{off,(M\cdot V189)\cdot sR1,j}[M_{PBM}\cdot V_{189}\cdot sR1]_{j} \end{matrix}$$

$$\begin{matrix} d[P2\cdot M_{PBM}]_{j}/dt=k_{on,P2\cdot M,j}[P2]_{j}[M_{PBM}]_{j}-k_{off,P2\cdot M,j}[P2\cdot M_{PBM}]_{j} \\ -k_{on,(M\cdot P2)\cdot sR1,j}[M_{PBM}\cdot P2]_{j}[sR1]_{j}+k_{off,(M\cdot P2)\cdot sR1,j}[M_{PBM}\cdot P2\cdot sR1]_{j} \end{matrix}$$

$$\begin{matrix} d[sR1\cdot M_{EBM}]_{j}/dt=k_{on,sR1\cdot M,j}[sR1]_{j}[M_{EBM}]_{j}-k_{off,sR1\cdot M,j}[sR1\cdot M_{EBM}]_{j} \\ -k_{on,(M\cdot sR1)\cdot V121,j}[M_{EBM}\cdot sR1]_{j}[V_{121}]_{j}+k_{off,(M\cdot sR1)\cdot V121,j}[M_{EBM}\cdot sR1\cdot V_{121}]_{j} \\ -k_{on,(M\cdot sR1)\cdot P1,j}[M_{EBM}\cdot sR1]_{j}[P1]_{j}+k_{off,(M\cdot sR1)\cdot P1,j}[M_{EBM}\cdot sR1\cdot P1]_{j} \end{matrix}$$

$$\begin{matrix} d[sR1\cdot M_{ECM}]_{j}/dt=k_{on,sR1\cdot M,j}[sR1]_{j}[M_{ECM}]_{j}-k_{off,sR1\cdot M,j}[sR1\cdot M_{ECM}]_{j} \\ -k_{on,(M\cdot sR1)\cdot V121,j}[M_{ECM}\cdot sR1]_{j}[V_{121}]_{j}+k_{off,(M\cdot sR1)\cdot V121,j}[M_{ECM}\cdot sR1\cdot V_{121}]_{j} \\ -k_{on,(M\cdot sR1)\cdot P1,j}[M_{ECM}\cdot sR1]_{j}[P1]_{j}+k_{off,(M\cdot sR1)\cdot P1,j}[M_{ECM}\cdot sR1\cdot P1]_{j} \end{matrix}$$

$$\begin{matrix} d[sR1\cdot M_{PBM}]_{j}/dt=k_{on,sR1\cdot M,j}[sR1]_{j}[M_{PBM}]_{j}-k_{off,sR1\cdot M,j}[sR1\cdot M_{PBM}]_{j} \\ -k_{on,(M\cdot sR1)\cdot V121,j}[M_{PBM}\cdot sR1]_{j}[V_{121}]_{j}+k_{off,(M\cdot sR1)\cdot V121,j}[M_{PBM}\cdot sR1\cdot V_{121}]_{j} \\ -k_{on,(M\cdot sR1)\cdot P1,j}[M_{PBM}\cdot sR1]_{j}[P1]_{j}+k_{off,(M\cdot sR1)\cdot P1,j}[M_{PBM}\cdot sR1\cdot P1]_{j} \end{matrix}$$

$$\begin{matrix} d[M_{EBM}\cdot V_{165}\cdot sR1]_{j}/dt=k_{on,M\cdot(V165\cdot sR1),j}[M_{EBM}]_{j}[V_{165}\cdot sR1]_{j}-k_{off,M\cdot(V165\cdot sR1),j}[M_{EBM}\cdot V_{165}\cdot sR1]_{j} \\ +k_{on,(M\cdot V165)\cdot sR1,j}[M_{EBM}\cdot V_{165}]_{j}[sR1]_{j}-k_{off,(M\cdot V165)\cdot sR1,j}[M_{EBM}\cdot V_{165}\cdot sR1]_{j} \end{matrix}$$

$$\begin{matrix} d[M_{EBM}\cdot V_{189}\cdot sR1]_{j}/dt=k_{on,M\cdot(V189\cdot sR1),j}[M_{EBM}]_{j}[V_{189}\cdot sR1]_{j}-k_{off,M\cdot(V189\cdot sR1),j}[M_{EBM}\cdot V_{189}\cdot sR1]_{j} \\ +k_{on,(M\cdot V189)\cdot sR1,j}[M_{EBM}\cdot V_{189}]_{j}[sR1]_{j}-k_{off,(M\cdot V189)\cdot sR1,j}[M_{EBM}\cdot V_{189}\cdot sR1]_{j} \end{matrix}$$

$$\begin{matrix} d[M_{EBM}\cdot P2\cdot sR1]_{j}/dt=k_{on,M\cdot(P2\cdot sR1),j}[M_{EBM}]_{j}[P2\cdot sR1]_{j}-k_{off,M\cdot(P2\cdot sR1),j}[M_{EBM}\cdot P2\cdot sR1]_{j} \\ +k_{on,(M\cdot P2)\cdot sR1,j}[M_{EBM}\cdot P2]_{j}[sR1]_{j}-k_{off,(M\cdot P2)\cdot sR1,j}[M_{EBM}\cdot P2\cdot sR1]_{j} \end{matrix}$$

$$\begin{matrix} d[V_{121}\cdot sR1\cdot M_{EBM}]_{j}/dt=k_{on,(V121\cdot sR1)\cdot M,j}[V_{121}\cdot sR1]_{j}[M_{EBM}]_{j}-k_{off,(V121\cdot sR1)\cdot M,j}[V_{121}\cdot sR1\cdot M_{EBM}]_{j} \\ +k_{on,V121\cdot(sR1\cdot M),j}[V_{121}]_{j}[sR1\cdot M_{EBM}]_{j}-k_{off,V121\cdot(sR1\cdot M),j}[V_{121}\cdot sR1\cdot M_{EBM}]_{j} \end{matrix}$$

$$\begin{matrix} d[P1\cdot sR1\cdot M_{EBM}]_{j}/dt=k_{on,(P1\cdot sR1)\cdot M,j}[P1\cdot sR1]_{j}[M_{EBM}]_{j}-k_{off,(P1\cdot sR1)\cdot M,j}[P1\cdot sR1\cdot M_{EBM}]_{j} \\ +k_{on,P1\cdot(sR1\cdot M),j}[P1]_{j}[sR1\cdot M_{EBM}]_{j}-k_{off,P1\cdot(sR1\cdot M),j}[P1\cdot sR1\cdot M_{EBM}]_{j} \end{matrix}$$

$$\begin{matrix} d[M_{ECM}\cdot V_{165}\cdot sR1]_{j}/dt=k_{on,M\cdot(V165\cdot sR1),j}[M_{ECM}]_{j}[V_{165}\cdot sR1]_{j}-k_{off,M\cdot(V165\cdot sR1),j}[M_{ECM}\cdot V_{165}\cdot sR1]_{j} \\ +k_{on,(M\cdot V165)\cdot sR1,j}[M_{ECM}\cdot V_{165}]_{j}[sR1]_{j}-k_{off,(M\cdot V165)\cdot sR1,j}[M_{ECM}\cdot V_{165}\cdot sR1]_{j} \end{matrix}$$

$$\begin{matrix} d[M_{ECM}\cdot V_{165}\cdot sR1]_{j}/dt=k_{on,M\cdot(V165\cdot sR1),j}[M_{ECM}]_{j}[V_{165}\cdot sR1]_{j}-k_{off,M\cdot(V165\cdot sR1),j}[M_{ECM}\cdot V_{165}\cdot sR1]_{j} \\ +k_{on,(M\cdot V165)\cdot sR1,j}[M_{ECM}\cdot V_{165}]_{j}[sR1]_{j}-k_{off,(M\cdot V165)\cdot sR1,j}[M_{ECM}\cdot V_{165}\cdot sR1]_{j} \end{matrix}$$

$$\begin{matrix} d[M_{ECM}\cdot V_{189}\cdot sR1]_{j}/dt=k_{on,M\cdot(V189\cdot sR1),j}[M_{ECM}]_{j}[V_{189}\cdot sR1]_{j}-k_{off,M\cdot(V189\cdot sR1),j}[M_{ECM}\cdot V_{189}\cdot sR1]_{j} \\ +k_{on,(M\cdot V189)\cdot sR1,j}[M_{ECM}\cdot V_{189}]_{j}[sR1]_{j}-k_{off,(M\cdot V189)\cdot sR1,j}[M_{ECM}\cdot V_{189}\cdot sR1]_{j} \end{matrix}$$

$$\begin{matrix} d[M_{ECM}\cdot P2\cdot sR1]_{j}/dt=k_{on,M\cdot(P2\cdot sR1),j}[M_{ECM}]_{j}[P2\cdot sR1]_{j}-k_{off,M\cdot(P2\cdot sR1),j}[M_{ECM}\cdot P2\cdot sR1]_{j} \\ +k_{on,(M\cdot P2)\cdot sR1,j}[M_{ECM}\cdot P2]_{j}[sR1]_{j}-k_{off,(M\cdot P2)\cdot sR1,j}[M_{ECM}\cdot P2\cdot sR1]_{j} \end{matrix}$$

$$\begin{matrix} d[V_{121}\cdot sR1\cdot M_{ECM}]_{j}/dt=k_{on,(V121\cdot sR1)\cdot M,j}[V_{121}\cdot sR1]_{j}[M_{ECM}]_{j}-k_{off,(V121\cdot sR1)\cdot M,j}[V_{121}\cdot sR1\cdot M_{ECM}]_{j} \\ +k_{on,V121\cdot(sR1\cdot M),j}[V_{121}]_{j}[sR1\cdot M_{ECM}]_{j}-k_{off,V121\cdot(sR1\cdot M),j}[V_{121}\cdot sR1\cdot M_{ECM}]_{j} \end{matrix}$$

$$\begin{matrix} d[P1\cdot sR1\cdot M_{ECM}]_{j}/dt=k_{on,(P1\cdot sR1)\cdot M,j}[P1\cdot sR1]_{j}[M_{ECM}]_{j}-k_{off,(P1\cdot sR1)\cdot M,j}[P1\cdot sR1\cdot M_{ECM}]_{j} \\ +k_{on,P1\cdot(sR1\cdot M),j}[P1]_{j}[sR1\cdot M_{ECM}]_{j}-k_{off,P1\cdot(sR1\cdot M),j}[P1\cdot sR1\cdot M_{ECM}]_{j} \end{matrix}$$

$$\begin{matrix} d[M_{PBM}\cdot V_{165}\cdot sR1]_{j}/dt=k_{on,M\cdot(V165\cdot sR1),j}[M_{PBM}]_{j}[V_{165}\cdot sR1]_{j}-k_{off,M\cdot(V165\cdot sR1),j}[M_{PBM}\cdot V_{165}\cdot sR1]_{j} \\ +k_{on,(M\cdot V165)\cdot sR1,j}[M_{PBM}\cdot V_{165}]_{j}[sR1]_{j}-k_{off,(M\cdot V165)\cdot sR1,j}[M_{PBM}\cdot V_{165}\cdot sR1]_{j} \end{matrix}$$

$$\begin{matrix} d[M_{PBM}\cdot V_{189}\cdot sR1]_{j}/dt=k_{on,M\cdot(V189\cdot sR1),j}[M_{PBM}]_{j}[V_{189}\cdot sR1]_{j}-k_{off,M\cdot(V189\cdot sR1),j}[M_{PBM}\cdot V_{189}\cdot sR1]_{j} \\ +k_{on,(M\cdot V189)\cdot sR1,j}[M_{PBM}\cdot V_{189}]_{j}[sR1]_{j}-k_{off,(M\cdot V189)\cdot sR1,j}[M_{PBM}\cdot V_{189}\cdot sR1]_{j} \end{matrix}$$

$$\begin{matrix} d[M_{PBM}\cdot P2\cdot sR1]_{j}/dt=k_{on,M\cdot(P2\cdot sR1),j}[M_{PBM}]_{j}[P2\cdot sR1]_{j}-k_{off,M\cdot(P2\cdot sR1),j}[M_{PBM}\cdot P2\cdot sR1]_{j} \\ +k_{on,(M\cdot P2)\cdot sR1,j}[M_{PBM}\cdot P2]_{j}[sR1]_{j}-k_{off,(M\cdot P2)\cdot sR1,j}[M_{PBM}\cdot P2\cdot sR1]_{j} \end{matrix}$$

$$\begin{matrix} d[V_{121}\cdot sR1\cdot M_{PBM}]_{j}/dt=k_{on,(V121\cdot sR1)\cdot M,j}[V_{121}\cdot sR1]_{j}[M_{PBM}]_{j}-k_{off,(V121\cdot sR1)\cdot M,j}[V_{121}\cdot sR1\cdot M_{PBM}]_{j} \\ +k_{on,V121\cdot(sR1\cdot M),j}[V_{121}]_{j}[sR1\cdot M_{PBM}]_{j}-k_{off,V121\cdot(sR1\cdot M),j}[V_{121}\cdot sR1\cdot M_{PBM}]_{j} \end{matrix}$$

$$\begin{matrix} d[P1\cdot sR1\cdot M_{PBM}]_{j}/dt=k_{on,(P1\cdot sR1)\cdot M,j}[P1\cdot sR1]_{j}[M_{PBM}]_{j}-k_{off,(P1\cdot sR1)\cdot M,j}[P1\cdot sR1\cdot M_{PBM}]_{j} \\ +k_{on,P1\cdot(sR1\cdot M),j}[P1]_{j}[sR1\cdot M_{PBM}]_{j}-k_{off,P1\cdot(sR1\cdot M),j}[P1\cdot sR1\cdot M_{PBM}]_{j} \end{matrix}$$

Abluminal Endothelial Cell Surface. These 27 equations represent molecular species present on the surface of endothelial cells, as summarized in **Figure 1E**. The binding rates (k_on_) and unbinding rates (k_off_) are given in **Tables 2 & 3**, with units of M^-1^s^-1^ for binding of ligands to receptors, and (moles/cm^2^)^-1^s^-1^ for coupling of cell surface receptors. The fraction of EBM accessible to endothelial cell receptors is *f*. The trafficking rates, which depend on receptor ligation and binding to NRP1 (**Table S8**), are in units of s^-1^, where k_int­_ is the internalization rate, k_rec4_ is the recycling rate from early endosomes (Rab4/5) to the cell surface, and k_rec11­_ is the recycling rate from recycling endosomes (Rab11) to the cell surface. [R1_rab45_] is the concentration of unoccupied VEGFR1 in early (Rab4/5) endosomes. s is the production rate for free (unoccupied) receptors delivered to the cell surface (#/cm^2^/s, converted), tuned to match experimental measurements of surface receptor densities at steady-state (**Table 4**).

$$\begin{matrix} d[R2]_{j}/dt=s_{R2,j}-k_{\mathrm{int},R2,j}[R2]_{j}+k_{rec4,R2,j}[R2_{rab45}]+k_{rec11,R2,j}[R2_{rab11}] \\ -k_{on,V165\cdot R2,j}[V_{165}]_{j}[R2]_{j}+k_{off,V165\cdot R2,j}[V_{165}\cdot R2]_{j} \\ -k_{on,V189\cdot R2,j}[V_{189}]_{j}[R2]_{j}+k_{off,V189\cdot R2,j}[V_{189}\cdot R2]_{j} \\ -k_{on,V121\cdot R2,j}[V_{121}]_{j}[R2]_{j}+k_{off,V121\cdot R2,j}[V_{121}\cdot R2]_{j} \\ -k_{on,(M\cdot V165)\cdot R2,j}[M_{EBM}\cdot V_{165}]_{j}[R2]_{j}+k_{off,(M\cdot V165)\cdot R2,j}[M_{EBM}\cdot V_{165}\cdot R2]_{j} \\ -k_{on,(M\cdot V189)\cdot R2,j}[M_{EBM}\cdot V_{189}]_{j}[R2]_{j}+k_{off,(M\cdot V189)\cdot R2,j}[M_{EBM}\cdot V_{189}\cdot R2]_{j} \\ -k_{on,(N1\cdot V165)\cdot R2,j}[N1\cdot V_{165}]_{j}[R2]_{j}+k_{off,(N1\cdot V165)\cdot R2,j}[N1\cdot V_{165}\cdot R2]_{j} \\ -k_{on,(N1\cdot V189)\cdot R2,j}[N1\cdot V_{189}]_{j}[R2]_{j}+k_{off,(N1\cdot V189)\cdot R2,j}[N1\cdot V_{189}\cdot R2]_{j} \end{matrix}$$

$$\begin{matrix} d[V_{165}\cdot R1]_{j}/dt=-k_{\mathrm{int},V165\cdot R1,j}[V_{165}\cdot R1]_{j}+k_{rec4,V165\cdot R1,j}[V_{165}\cdot R1_{rab45}]+k_{rec11,V165\cdot R1,j}[V_{165}\cdot R1_{rab11}] \\ +k_{on,V165\cdot R1,j}[V_{165}]_{j}[R1]_{j}-k_{off,V165\cdot R1,j}[V_{165}\cdot R1]_{j} \\ -k_{on,M\cdot(V165\cdot R1),j}[M_{EBM}]_{j}[V_{165}\cdot R1]_{j}+k_{off,M\cdot(V165\cdot R1),j}[M_{EBM}\cdot V_{165}\cdot R1]_{j} \end{matrix}$$

$$\begin{matrix} d[V_{189}\cdot R1]_{j}/dt=-k_{\mathrm{int},V189\cdot R1,j}[V_{189}\cdot R1]_{j}+k_{rec4,V189\cdot R1,j}[V_{189}\cdot R1_{rab45}]+k_{rec11,V189\cdot R1,j}[V_{189}\cdot R1_{rab11}] \\ +k_{on,V189\cdot R1,j}[V_{189}]_{j}[R1]_{j}-k_{off,V189\cdot R1,j}[V_{189}\cdot R1]_{j} \\ -k_{on,M\cdot(V189\cdot R1),j}[M_{EBM}]_{j}[V_{189}\cdot R1]_{j}+k_{off,M\cdot(V189\cdot R1),j}[M_{EBM}\cdot V_{189}\cdot R1]_{j} \end{matrix}$$

$$\begin{matrix} d[V_{121}\cdot R1]_{j}/dt=-k_{\mathrm{int},V121\cdot R1,j}[V_{121}\cdot R1]_{j}+k_{rec4,V121\cdot R1,j}[V_{121}\cdot R1_{rab45}]+k_{rec11,V121\cdot R1,j}[V_{121}\cdot R1_{rab11}] \\ +k_{on,V121\cdot R1,j}[V_{121}]_{j}[R1]_{j}-k_{off,V121\cdot R1,j}[V_{121}\cdot R1]_{j} \\ -k_{on,(V121\cdot R1)\cdot N1,j}[V_{121}\cdot R1]_{j}[N1]_{j}+k_{off,(V121\cdot R1)\cdot N1,j}[V_{121}\cdot R1\cdot N1]_{j} \end{matrix}$$

$$\begin{matrix} d[P1\cdot R1]_{j}/dt=-k_{\mathrm{int},P1\cdot R1,j}[P1\cdot R1]_{j}+k_{rec4,P1\cdot R1,j}[P1\cdot R1_{rab45}]+k_{rec11,P1\cdot R1,j}[P1\cdot R1_{rab11}] \\ +k_{on,P1\cdot R1,j}[P1]_{j}[R1]_{j}-k_{off,P1\cdot R1,j}[P1\cdot R1]_{j} \\ -k_{on,(P1\cdot R1)\cdot N1,j}[P1\cdot R1]_{j}[N1]_{j}+k_{off,(P1\cdot R1)\cdot N1,j}[P1\cdot R1\cdot N1]_{j} \end{matrix}$$

$$\begin{matrix} d[P2\cdot R1]_{j}/dt=-k_{\mathrm{int},P2\cdot R1,j}[P2\cdot R1]_{j}+k_{rec4,P2\cdot R1,j}[P2\cdot R1_{rab45}]+k_{rec11,P2\cdot R1,j}[P2\cdot R1_{rab11}] \\ +k_{on,P2\cdot R1,j}[P2]_{j}[R1]_{j}-k_{off,P2\cdot R1,j}[P2\cdot R1]_{j} \\ -k_{on,M\cdot(P2\cdot R1),j}[M_{EBM}]_{j}[P2\cdot R1]_{j}+k_{off,M\cdot(P2\cdot R1),j}[M_{EBM}\cdot P2\cdot R1]_{j} \end{matrix}$$

$$\begin{matrix} d[V_{165}\cdot R2]_{j}/dt=-k_{\mathrm{int},V165\cdot R2,j}[V_{165}\cdot R2]_{j}+k_{rec4,V165\cdot R2,j}[V_{165}\cdot R2_{rab45}]+k_{rec11,V165\cdot R2,j}[V_{165}\cdot R2_{rab11}] \\ +k_{on,V165\cdot R2,j}[V_{165}]_{j}[R2]_{j}-k_{off,V165\cdot R2,j}[V_{165}\cdot R2]_{j} \\ -k_{on,M\cdot(V165\cdot R2),j}[M_{EBM}]_{j}[V_{165}\cdot R2]_{j}+k_{off,M\cdot(V165\cdot R2),j}[M_{EBM}\cdot V_{165}\cdot R2]_{j} \\ -k_{on,N1\cdot(V165\cdot R2),j}[N1]_{j}[V_{165}\cdot R2]_{j}+k_{off,N1\cdot(V165\cdot R2),j}[N1\cdot V_{165}\cdot R2]_{j} \end{matrix}$$

$$\begin{matrix} d[V_{189}\cdot R2]_{j}/dt=-k_{\mathrm{int},V189\cdot R2,j}[V_{189}\cdot R2]_{j}+k_{rec4,V189\cdot R2,j}[V_{189}\cdot R2_{rab45}]+k_{rec11,V189\cdot R2,j}[V_{189}\cdot R2_{rab11}] \\ +k_{on,V189\cdot R2,j}[V_{189}]_{j}[R2]_{j}-k_{off,V189\cdot R2,j}[V_{189}\cdot R2]_{j} \\ -k_{on,M\cdot(V189\cdot R2),j}[M_{EBM}]_{j}[V_{189}\cdot R2]_{j}+k_{off,M\cdot(V189\cdot R2),j}[M_{EBM}\cdot V_{189}\cdot R2]_{j} \\ -k_{on,N1\cdot(V189\cdot R2),j}[N1]_{j}[V_{189}\cdot R2]_{j}+k_{off,N1\cdot(V189\cdot R2),j}[N1\cdot V_{189}\cdot R2]_{j} \end{matrix}$$

$$\begin{matrix} d[V_{121}\cdot R2]_{j}/dt=-k_{\mathrm{int},V121\cdot R2,j}[V_{121}\cdot R2]_{j}+k_{rec4,V121\cdot R2,j}[V_{121}\cdot R2_{rab45}]+k_{rec11,V121\cdot R2,j}[V_{121}\cdot R2_{rab11}] \\ +k_{on,V121\cdot R2,j}[V_{121}]_{j}[R2]_{j}-k_{off,V121\cdot R2,j}[V_{121}\cdot R2]_{j} \end{matrix}$$

$$\begin{matrix} d[V_{165}\cdot N1]_{j}/dt=-k_{\mathrm{int},V165\cdot N1,j}[V_{165}\cdot N1]_{j}+k_{rec4,V165\cdot N1,j}[V_{165}\cdot N1_{rab45}]+k_{rec11,V165\cdot N1,j}[V_{165}\cdot N1_{rab11}] \\ +k_{on,V165\cdot N1,j}[V_{165}]_{j}[N1]_{j}-k_{off,V165\cdot N1,j}[V_{165}\cdot N1]_{j} \\ -k_{on,(N1\cdot V165)\cdot R2,j}[N1\cdot V_{165}]_{j}[R2]_{j}+k_{off,(N1\cdot V165)\cdot R2,j}[N1\cdot V_{165}\cdot R2]_{j} \end{matrix}$$

$$\begin{matrix} d[V_{189}\cdot N1]_{j}/dt=-k_{\mathrm{int},V189\cdot N1,j}[V_{189}\cdot N1]_{j}+k_{rec4,V189\cdot N1,j}[V_{189}\cdot N1_{rab45}]+k_{rec11,V189\cdot N1,j}[V_{189}\cdot N1_{rab11}] \\ +k_{on,V189\cdot N1,j}[V_{189}]_{j}[N1]_{j}-k_{off,V189\cdot N1,j}[V_{189}\cdot N1]_{j} \\ -k_{on,(N1\cdot V189)\cdot R2,j}[N1\cdot V_{189}]_{j}[R2]_{j}+k_{off,(N1\cdot V189)\cdot R2,j}[N1\cdot V_{189}\cdot R2]_{j} \end{matrix}$$

$$\begin{matrix} d[P2\cdot N1]_{j}/dt=-k_{\mathrm{int},P2\cdot N1,j}[P2\cdot N1]_{j}+k_{rec4,P2\cdot N1,j}[P2\cdot N1_{rab45}]+k_{rec11,P2\cdot N1,j}[P2\cdot N1_{rab11}] \\ +k_{on,P2\cdot N1,j}[P2]_{j}[N1]_{j}-k_{off,P2\cdot N1,j}[P2\cdot N1]_{j} \\ -k_{on,(N1\cdot P2)\cdot R2,j}[N1\cdot P2]_{j}[R2]_{j}+k_{off,(N1\cdot P2)\cdot R2,j}[N1\cdot P2\cdot R2]_{j} \end{matrix}$$

$$\begin{matrix} d[N1\cdot R1]_{j}/dt=-k_{\mathrm{int},N1\cdot R1,j}[N1\cdot R1]_{j}+k_{rec4,N1\cdot R1,j}[N1\cdot R1_{rab45}]+k_{rec11,N1\cdot R1,j}[N1\cdot R1_{rab11}] \\ +k_{on,N1\cdot R1,j}[N1]_{j}[R1]_{j}-k_{off,N1\cdot R1,j}[N1\cdot R1]_{j} \\ -k_{on,V121\cdot(N1\cdot R1),j}[V_{121}]_{j}[N1\cdot R1]_{j}+k_{off,V121\cdot(N1\cdot R1),j}[V_{121}\cdot R1\cdot N1]_{j} \\ -k_{on,P1\cdot(N1\cdot R1),j}[P1]_{j}[N1\cdot R1]_{j}+k_{off,P1\cdot(N1\cdot R1),j}[P1\cdot R1\cdot N1]_{j} \end{matrix}$$

$$\begin{matrix} d[N1\cdot sR1]_{j}/dt=-k_{\mathrm{int},N1\cdot sR1,j}[N1\cdot sR1]_{j}+k_{rec4,N1\cdot sR1,j}[N1\cdot sR1_{rab45}]+k_{rec11,N1\cdot sR1,j}[N1\cdot sR1_{rab11}] \\ +k_{on,N1\cdot sR1,j}[N1]_{j}[sR1]_{j}-k_{off,N1\cdot sR1,j}[N1\cdot sR1]_{j} \\ -k_{on,V121\cdot(N1\cdot sR1),j}[V_{121}]_{j}[N1\cdot sR1]_{j}+k_{off,V121\cdot(N1\cdot sR1),j}[V_{121}\cdot sR1\cdot N1]_{j} \\ -k_{on,P1\cdot(N1\cdot sR1),j}[P1]_{j}[N1\cdot sR1]_{j}+k_{off,P1\cdot(N1\cdot sR1),j}[P1\cdot sR1\cdot N1]_{j} \end{matrix}$$

$$\begin{matrix} d[N1\cdot V_{165}\cdot R2]_{j}/dt=-k_{\mathrm{int},N1\cdot V165\cdot R2,j}[N1\cdot V_{165}\cdot R2]_{j}+k_{rec4,N1\cdot V165\cdot R2,j}[N1\cdot V_{165}\cdot R2_{rab45}] \\ +k_{rec11,N1\cdot V165\cdot R2,j}[N1\cdot V_{165}\cdot R2_{rab11}] \\ +k_{on,R2\cdot(N1\cdot V165),j}[R2]_{j}[V_{165}\cdot N1]_{j}-k_{off,R2\cdot(N1\cdot V165),j}[N1\cdot V_{165}\cdot R2]_{j} \\ +k_{on,N1\cdot(V165\cdot R2),j}[N1]_{j}[V_{165}\cdot R2]_{j}-k_{off,N1\cdot(V165\cdot R2),j}[N1\cdot V_{165}\cdot R2]_{j} \end{matrix}$$

$$\begin{matrix} d[N1\cdot V_{189}\cdot R2]_{j}/dt=-k_{\mathrm{int},N1\cdot V189\cdot R2,j}[N1\cdot V_{189}\cdot R2]_{j}+k_{rec4,N1\cdot V189\cdot R2,j}[N1\cdot V_{189}\cdot R2_{rab45}] \\ +k_{rec11,N1\cdot V189\cdot R2,j}[N1\cdot V_{189}\cdot R2_{rab11}] \\ +k_{on,R2\cdot(V189\cdot N1),j}[R2]_{j}[V_{189}\cdot N1]_{j}-k_{off,R2\cdot(N1\cdot V189),j}[N1\cdot V_{189}\cdot R2]_{j} \\ +k_{on,N1\cdot(V189\cdot R2),j}[N1]_{j}[V_{189}\cdot R2]_{j}-k_{off,N1\cdot(V189\cdot R2),j}[N1\cdot V_{189}\cdot R2]_{j} \end{matrix}$$

$$\begin{matrix} d[V_{121}\cdot R1\cdot N1]_{j}/dt=-k_{\mathrm{int},V121\cdot R1\cdot N1,j}[V_{121}\cdot R1\cdot N1]_{j}+k_{rec4,V121\cdot R1\cdot N1,j}[V_{121}\cdot R1\cdot N1_{rab45}] \\ +k_{rec11,V121\cdot R1\cdot N1,j}[V_{121}\cdot R1\cdot N1_{rab11}] \\ +k_{on,V121\cdot(R1\cdot N1),j}[V_{121}]_{j}[R1\cdot N1]_{j}-k_{off,V121\cdot(R1\cdot N1),j}[V_{121}\cdot R1\cdot N1]_{j} \\ +k_{on,(V121\cdot R1)\cdot N1,j}[V_{121}\cdot R1]_{j}[N1]_{j}-k_{off,(V121\cdot R1)\cdot N1,j}[V_{121}\cdot R1\cdot N1]_{j} \end{matrix}$$

$$\begin{matrix} d[P1\cdot R1\cdot N1]_{j}/dt=-k_{\mathrm{int},P1\cdot R1\cdot N1,j}[P1\cdot R1\cdot N1]_{j}+k_{rec4,P1\cdot R1\cdot N1,j}[P1\cdot R1\cdot N1_{rab45}] \\ +k_{rec11,P1\cdot R1\cdot N1,j}[P1\cdot R1\cdot N1_{rab11}] \\ +k_{on,P1\cdot(R1\cdot N1),j}[P1]_{j}[R1\cdot N1]_{j}-k_{off,P1\cdot(R1\cdot N1),j}[P1\cdot R1\cdot N1]_{j} \\ +k_{on,(P1\cdot R1)\cdot N1,j}[P1\cdot R1]_{j}[N1]_{j}-k_{off,(P1\cdot R1)\cdot N1,j}[P1\cdot R1\cdot N1]_{j} \end{matrix}$$

$$\begin{matrix} d[V_{121}\cdot sR1\cdot N1]_{j}/dt=-k_{\mathrm{int},V121\cdot sR1\cdot N1,j}[V_{121}\cdot sR1\cdot N1]_{j}+k_{rec4,V121\cdot sR1\cdot N1,j}[V_{121}\cdot sR1\cdot N1_{rab45}] \\ +k_{rec11,V121\cdot sR1\cdot N1,j}[V_{121}\cdot sR1\cdot N1_{rab11}] \\ +k_{on,V121\cdot(sR1\cdot N1),j}[V_{121}]_{j}[sR1\cdot N1]_{j}-k_{off,V121\cdot(sR1\cdot N1),j}[V_{121}\cdot sR1\cdot N1]_{j} \\ +k_{on,(V121\cdot sR1)\cdot N1,j}[V_{121}\cdot sR1]_{j}[N1]_{j}-k_{off,(V121\cdot sR1)\cdot N1,j}[V_{121}\cdot sR1\cdot N1]_{j} \end{matrix}$$

$$\begin{matrix} d[P1\cdot sR1\cdot N1]_{j}/dt=-k_{\mathrm{int},P1\cdot sR1\cdot N1,j}[P1\cdot sR1\cdot N1]_{j}+k_{rec4,P1\cdot sR1\cdot N1,j}[P1\cdot sR1\cdot N1_{rab45}] \\ +k_{rec11,P1\cdot sR1\cdot N1,j}[P1\cdot sR1\cdot N1_{rab11}] \\ +k_{on,P1\cdot(sR1\cdot N1),j}[P1]_{j}[sR1\cdot N1]_{j}-k_{off,P1\cdot(sR1\cdot N1),j}[P1\cdot sR1\cdot N1]_{j} \\ +k_{on,(P1\cdot sR1)\cdot N1,j}[P1\cdot sR1]_{j}[N1]_{j}-k_{off,(P1\cdot sR1)\cdot N1,j}[P1\cdot sR1\cdot N1]_{j} \end{matrix}$$

$$\begin{matrix} d[M_{EBM}\cdot V_{165}\cdot R2]_{j}/dt=k_{on,(M\cdot V165)\cdot R2,j}\cdot f\cdot[M_{EBM}\cdot V_{165}]_{j}[R2]_{j}-k_{off,(M\cdot V165)\cdot R2,j}[M_{EBM}\cdot V_{165}\cdot R2]_{j} \\ +k_{on,M\cdot(V165\cdot R2),j}\cdot f\cdot[M_{EBM}]_{j}[V_{165}\cdot R2]_{j}-k_{off,M\cdot(V165\cdot R2),j}[M_{EBM}\cdot V_{165}\cdot R2]_{j} \end{matrix}$$

$$\begin{matrix} d[M_{EBM}\cdot V_{189}\cdot R2]_{j}/dt=k_{on,(M\cdot V189)\cdot R2,j}\cdot f\cdot[M_{EBM}\cdot V_{189}]_{j}[R2]_{j}-k_{off,(M\cdot V189)\cdot R2,j}[M_{EBM}\cdot V_{189}\cdot R2]_{j} \\ +k_{on,M\cdot(V189\cdot R2),j}\cdot f\cdot[M_{EBM}]_{j}[V_{189}\cdot R2]_{j}-k_{off,M\cdot(V189\cdot R2),j}[M_{EBM}\cdot V_{189}\cdot R2]_{j} \end{matrix}$$

$$\begin{matrix} d[M_{EBM}\cdot V_{165}\cdot R1]_{j}/dt=k_{on,(M\cdot V165)\cdot R1,j}\cdot f\cdot[M_{EBM}\cdot V_{165}]_{j}[R1]_{j}-k_{off,(M\cdot V165)\cdot R1,j}[M_{EBM}\cdot V_{165}\cdot R1]_{j} \\ +k_{on,M\cdot(V165\cdot R1),j}\cdot f\cdot[M_{EBM}]_{j}[V_{165}\cdot R1]_{j}-k_{off,M\cdot(V165\cdot R1),j}[M_{EBM}\cdot V_{165}\cdot R1]_{j} \end{matrix}$$

$$\begin{matrix} d[M_{EBM}\cdot V_{189}\cdot R1]_{j}/dt=k_{on,(M\cdot V189)\cdot R1,j}\cdot f\cdot[M_{EBM}\cdot V_{189}]_{j}[R1]_{j}-k_{off,(M\cdot V189)\cdot R1,j}[M_{EBM}\cdot V_{189}\cdot R1]_{j} \\ +k_{on,M\cdot(V189\cdot R1),j}\cdot f\cdot[M_{EBM}]_{j}[V_{189}\cdot R1]_{j}-k_{off,M\cdot(V189\cdot R1),j}[M_{EBM}\cdot V_{189}\cdot R1]_{j} \end{matrix}$$

$$\begin{matrix} d[M_{EBM}\cdot P2\cdot R1]_{j}/dt=k_{on,(M\cdot P2)\cdot R1,j}\cdot f\cdot[M_{EBM}\cdot P2]_{j}[R1]_{j}-k_{off,(M\cdot P2)\cdot R1,j}[M_{EBM}\cdot P2\cdot R1]_{j} \\ +k_{on,M\cdot(P2\cdot R1),j}\cdot f\cdot[M_{EBM}]_{j}[P2\cdot R1]_{j}-k_{off,M\cdot(P2\cdot R1),j}[M_{EBM}\cdot P2\cdot R1]_{j} \end{matrix}$$

Detailed VEGFR2 Phosphorylation Reactions. Here we show one example equation (unoccupied cell surface VEGFR2 phosphorylated only on tyrosine Y1175) demonstrating the site-specific phosphorylation and dephosphorylation of VEGFR2 on tyrosines 951, 1175, and 1214 (**Figure 1D**). Phosphorylation is assumed to be independent on each site, giving 8 possible combinations: no phosphorylation, pY951 only, pY1175 only, pY1214 only, pY951 and pY1175, pY951 and pY1214, pY1175 and pY1214, and all three sites phosphorylated. VEGFR2 can be phosphorylated in any of these patterns on the cell surface, in early (Rab4/5) endosomes, or in recycling (Rab11) endosomes. The phosphorylation and dephosphorylation rates (**Table S9**) vary by subcellular location and with ligation, but are assumed to be independent of NRP1 and HSPG binding. The phosphorylation state of VEGFR2 is assumed not to alter is binding or trafficking properties. We focus here on pY1175 and pY1214 because the parameters for these sites are better constrained than those for pY951. Total phosphorylated VEGFR2 (pR2) is approximated as the sum of all VEGFR2 phosphorylated on at least one site. The full set of equations for phosphorylation of VEGFR2 in all complexes and all locations is omitted for the sake of brevity.

Endothelial Endosomes. These 54 equations represent molecular species within early signaling (Rab4/5) or recycling (Rab11) endosomes in endothelial cells. Here, k_4to11­_ is the trafficking rate from early (Rab45) to recycling (Rab11) endosomes, and k_degr_ is the rate of degradation of species from early Rab4/5 endosomes. [R1_rab45_] is the concentration of unoccupied VEGFR1 in early (Rab4/5) endosomes.

### Rab4/5 Early Signaling Endosomes

$$\begin{matrix} d[R1_{rab45}]_{j}/dt=k_{\mathrm{int},R1,j}[R1]_{j}-k_{rec4,R1,j}[R1_{rab45}]-k_{4to11,R1,j}[R1_{rab45}]-k_{\deg r,R1,j}[R1_{rab45}] \\ -k_{on,V165\cdot R1,j}[V_{165,rab45}]_{j}[R1_{rab45}]_{j}+k_{off,V165\cdot R1,j}[V_{165}\cdot R1_{rab45}]_{j} \\ -k_{on,V189\cdot R1,j}[V_{189,rab45}]_{j}[R1_{rab45}]_{j}+k_{off,V189\cdot R1,j}[V_{189}\cdot R1_{rab45}]_{j} \\ -k_{on,V121\cdot R1,j}[V_{121,rab45}]_{j}[R1_{rab45}]_{j}+k_{off,V121\cdot R1,j}[V_{121}\cdot R1_{rab45}]_{j} \\ -k_{on,P1\cdot R1,j}[P1_{rab45}]_{j}[R1_{rab45}]_{j}+k_{off,P1\cdot R1,j}[P1\cdot R1_{rab45}]_{j} \\ -k_{on,P2\cdot R1,j}[P2_{rab45}]_{j}[R1_{rab45}]_{j}+k_{off,P2\cdot R1,j}[P2\cdot R1_{rab45}]_{j} \\ -k_{on,N1\cdot R1,j}[N1_{rab45}]_{j}[R1_{rab45}]_{j}+k_{off,N1\cdot R1,j}[N1\cdot R1_{rab45}]_{j} \end{matrix}$$

$$\begin{matrix} d[R2_{rab45}]_{j}/dt=k_{\mathrm{int},R2,j}[R2]_{j}-k_{rec4,R2,j}[R2_{rab45}]-k_{4to11,R2,j}[R2_{rab45}]-k_{\deg r,R2,j}[R2_{rab45}] \\ -k_{on,V165\cdot R2,j}[V_{165,rab45}]_{j}[R2_{rab45}]_{j}+k_{off,V165\cdot R2,j}[V_{165}\cdot R2_{rab45}]_{j} \\ -k_{on,V189\cdot R2,j}[V_{189,rab45}]_{j}[R2_{rab45}]_{j}+k_{off,V189\cdot R2,j}[V_{189}\cdot R2_{rab45}]_{j} \\ -k_{on,V121\cdot R2,j}[V_{121,rab45}]_{j}[R2_{rab45}]_{j}+k_{off,V121\cdot R2,j}[V_{121}\cdot R2_{rab45}]_{j} \\ -k_{on,(N1\cdot V165)\cdot R2,j}[N1\cdot V_{165,rab45}]_{j}[R2_{rab45}]_{j}+k_{off,(N1\cdot V165)\cdot R2,j}[N1\cdot V_{165}\cdot R2_{rab45}]_{j} \\ -k_{on,(N1\cdot V189)\cdot R2,j}[N1\cdot V_{189,rab45}]_{j}[R2_{rab45}]_{j}+k_{off,(N1\cdot V189)\cdot R2,j}[N1\cdot V_{189}\cdot R2_{rab45}]_{j} \end{matrix}$$

$$\begin{matrix} d[V_{165}\cdot R1_{rab45}]_{j}/dt=k_{\mathrm{int},V165\cdot R1,j}[V_{165}\cdot R1]_{j}-k_{rec4,V165\cdot R1,j}[V_{165}\cdot R1_{rab45}] \\ -k_{4to11,V165\cdot R1,j}[V_{165}\cdot R1_{rab45}]-k_{\deg r,V165\cdot R1,j}[V_{165}\cdot R1_{rab45}] \\ +k_{on,V165\cdot R1,j}[V_{165,rab45}]_{j}[R1_{rab45}]_{j}-k_{off,V165\cdot R1,j}[V_{165}\cdot R1_{rab45}]_{j} \end{matrix}$$

$$\begin{matrix} d[V_{189}\cdot R1_{rab45}]_{j}/dt=k_{\mathrm{int},V189\cdot R1,j}[V_{189}\cdot R1]_{j}-k_{rec4,V189\cdot R1,j}[V_{189}\cdot R1_{rab45}] \\ -k_{4to11,V189\cdot R1,j}[V_{189}\cdot R1_{rab45}]-k_{\deg r,V189\cdot R1,j}[V_{189}\cdot R1_{rab45}] \\ +k_{on,V189\cdot R1,j}[V_{189,rab45}]_{j}[R1_{rab45}]_{j}-k_{off,V189\cdot R1,j}[V_{189}\cdot R1_{rab45}]_{j} \end{matrix}$$

$$\begin{matrix} d[V_{121}\cdot R1_{rab45}]_{j}/dt=k_{\mathrm{int},V121\cdot R1,j}[V_{121}\cdot R1]_{j}-k_{rec4,V121\cdot R1,j}[V_{121}\cdot R1_{rab45}] \\ -k_{4to11,V121\cdot R1,j}[V_{121}\cdot R1_{rab45}]-k_{\deg r,V121\cdot R1,j}[V_{121}\cdot R1_{rab45}] \\ +k_{on,V121\cdot R1,j}[V_{121,rab45}]_{j}[R1_{rab45}]_{j}-k_{off,V121\cdot R1,j}[V_{121}\cdot R1_{rab45}]_{j} \\ -k_{on,(V121\cdot R1)\cdot N1,j}[V_{121}\cdot R1_{rab45}]_{j}[N1_{rab45}]_{j}+k_{off,(V121\cdot R1)\cdot N1,j}[V_{121}\cdot R1\cdot N1_{rab45}]_{j} \end{matrix}$$

$$\begin{matrix} d[P1\cdot R1_{rab45}]_{j}/dt=k_{\mathrm{int},P1\cdot R1,j}[P1\cdot R1]_{j}-k_{rec4,P1\cdot R1,j}[P1\cdot R1_{rab45}] \\ -k_{4to11,P1\cdot R1,j}[P1\cdot R1_{rab45}]-k_{\deg r,P1\cdot R1,j}[P1\cdot R1_{rab45}] \\ +k_{on,P1\cdot R1,j}[P1_{rab45}]_{j}[R1_{rab45}]_{j}-k_{off,P1\cdot R1,j}[P1\cdot R1_{rab45}]_{j} \\ -k_{on,(P1\cdot R1)\cdot N1,j}[P1\cdot R1_{rab45}]_{j}[N1_{rab45}]_{j}+k_{off,(P1\cdot R1)\cdot N1,j}[P1\cdot R1\cdot N1_{rab45}]_{j} \end{matrix}$$

$$\begin{matrix} d[P2\cdot R1_{rab45}]_{j}/dt=k_{\mathrm{int},P2\cdot R1,j}[P2\cdot R1]_{j}-k_{rec4,P2\cdot R1,j}[P2\cdot R1_{rab45}] \\ -k_{4to11,P2\cdot R1,j}[P2\cdot R1_{rab45}]-k_{\deg r,P2\cdot R1,j}[P2\cdot R1_{rab45}] \\ +k_{on,P2\cdot R1,j}[P2_{rab45}]_{j}[R1_{rab45}]_{j}-k_{off,P2\cdot R1,j}[P2\cdot R1_{rab45}]_{j} \end{matrix}$$

$$\begin{matrix} d[V_{165}\cdot sR1_{rab45}]_{j}/dt=-k_{\deg r,V165\cdot sR1,j}[V_{165}\cdot sR1_{rab45}] \\ +k_{on,V165\cdot sR1,j}[V_{165,rab45}]_{j}[sR1_{rab45}]_{j}-k_{off,V165\cdot sR1,j}[V_{165}\cdot sR1_{rab45}]_{j} \end{matrix}$$

$$\begin{matrix} d[V_{189}\cdot sR1_{rab45}]_{j}/dt=-k_{\deg r,V189\cdot sR1,j}[V_{189}\cdot sR1_{rab45}] \\ +k_{on,V189\cdot sR1,j}[V_{189,rab45}]_{j}[sR1_{rab45}]_{j}-k_{off,V189\cdot sR1,j}[V_{189}\cdot sR1_{rab45}]_{j} \end{matrix}$$

$$\begin{matrix} d[V_{121}\cdot sR1_{rab45}]_{j}/dt=-k_{\deg r,V121\cdot sR1,j}[V_{121}\cdot sR1_{rab45}] \\ +k_{on,V121\cdot sR1,j}[V_{121,rab45}]_{j}[sR1_{rab45}]_{j}-k_{off,V121\cdot sR1,j}[V_{121}\cdot sR1_{rab45}]_{j} \\ -k_{on,(V121\cdot sR1)\cdot N1,j}[V_{121}\cdot sR1_{rab45}]_{j}[N1_{rab45}]_{j}+k_{off,(V121\cdot sR1)\cdot N1,j}[V_{121}\cdot sR1\cdot N1_{rab45}]_{j} \end{matrix}$$

$$\begin{matrix} d[P1\cdot sR1_{rab45}]_{j}/dt=-k_{\deg r,P1\cdot sR1,j}[P1\cdot sR1_{rab45}] \\ +k_{on,P1\cdot sR1,j}[P1_{rab45}]_{j}[sR1_{rab45}]_{j}-k_{off,P1\cdot sR1,j}[P1\cdot sR1_{rab45}]_{j} \\ -k_{on,(P1\cdot sR1)\cdot N1,j}[P1\cdot sR1_{rab45}]_{j}[N1_{rab45}]_{j}+k_{off,(P1\cdot sR1)\cdot N1,j}[P1\cdot sR1\cdot N1_{rab45}]_{j} \end{matrix}$$

$$\begin{matrix} d[P2\cdot sR1_{rab45}]_{j}/dt=-k_{\deg r,P2\cdot sR1,j}[P2\cdot sR1_{rab45}] \\ +k_{on,P2\cdot sR1,j}[P2_{rab45}]_{j}[sR1_{rab45}]_{j}-k_{off,P2\cdot sR1,j}[P2\cdot sR1_{rab45}]_{j} \end{matrix}$$

$$\begin{matrix} d[V_{165}\cdot R2_{rab45}]_{j}/dt=k_{\mathrm{int},V165\cdot R2,j}[V_{165}\cdot R2]_{j}-k_{rec4,V165\cdot R2,j}[V_{165}\cdot R2_{rab45}] \\ -k_{4to11,V165\cdot R2,j}[V_{165}\cdot R2_{rab45}]-k_{\deg r,V165\cdot R2,j}[V_{165}\cdot R2_{rab45}] \\ +k_{on,V165\cdot R2,j}[V_{165,rab45}]_{j}[R2_{rab45}]_{j}-k_{off,V165\cdot R2,j}[V_{165}\cdot R2_{rab45}]_{j} \\ -k_{on,N1\cdot(V165\cdot R2),j}[N1_{rab45}]_{j}[V_{165}\cdot R2_{rab45}]_{j}+k_{off,N1\cdot(V165\cdot R2),j}[N1\cdot V_{165}\cdot R2_{rab45}]_{j} \end{matrix}$$

$$\begin{matrix} d[V_{189}\cdot R2_{rab45}]_{j}/dt=k_{\mathrm{int},V189\cdot R2,j}[V_{189}\cdot R2]_{j}-k_{rec4,V189\cdot R2,j}[V_{189}\cdot R2_{rab45}] \\ -k_{4to11,V189\cdot R2,j}[V_{189}\cdot R2_{rab45}]-k_{\deg r,V189\cdot R2,j}[V_{189}\cdot R2_{rab45}] \\ +k_{on,V189\cdot R2,j}[V_{189,rab45}]_{j}[R2_{rab45}]_{j}-k_{off,V189\cdot R2,j}[V_{189}\cdot R2_{rab45}]_{j} \\ -k_{on,N1\cdot(V189\cdot R2),j}[N1_{rab45}]_{j}[V_{189}\cdot R2_{rab45}]_{j}+k_{off,N1\cdot(V189\cdot R2),j}[N1\cdot V_{189}\cdot R2_{rab45}]_{j} \end{matrix}$$

$$\begin{matrix} d[V_{121}\cdot R2_{rab45}]_{j}/dt=k_{\mathrm{int},V121\cdot R2,j}[V_{121}\cdot R2]_{j}-k_{rec4,V121\cdot R2,j}[V_{121}\cdot R2_{rab45}] \\ -k_{4to11,V121\cdot R2,j}[V_{121}\cdot R2_{rab45}]-k_{\deg r,V121\cdot R2,j}[V_{121}\cdot R2_{rab45}] \\ +k_{on,V121\cdot R2,j}[V_{121,rab45}]_{j}[R2_{rab45}]_{j}-k_{off,V121\cdot R2,j}[V_{121}\cdot R2_{rab45}]_{j} \end{matrix}$$

$$\begin{matrix} d[V_{165}\cdot N1_{rab45}]_{j}/dt=k_{\mathrm{int},V165\cdot N1,j}[V_{165}\cdot N1]_{j}-k_{rec4,V165\cdot N1,j}[V_{165}\cdot N1_{rab45}] \\ -k_{4to11,V165\cdot N1,j}[V_{165}\cdot N1_{rab45}]-k_{\deg r,V165\cdot N1,j}[V_{165}\cdot N1_{rab45}] \\ +k_{on,V165\cdot N1,j}[V_{165,rab45}]_{j}[N1_{rab45}]_{j}-k_{off,V165\cdot N1,j}[V_{165}\cdot N1_{rab45}]_{j} \\ -k_{on,(N1\cdot V165)\cdot R2,j}[N1\cdot V_{165,rab45}]_{j}[R2_{rab45}]_{j}+k_{off,(N1\cdot V165)\cdot R2,j}[N1\cdot V_{165}\cdot R2_{rab45}]_{j} \end{matrix}$$

$$\begin{matrix} d[V_{189}\cdot N1_{rab45}]_{j}/dt=k_{\mathrm{int},V189\cdot N1,j}[V_{189}\cdot N1]_{j}-k_{rec4,V189\cdot N1,j}[V_{189}\cdot N1_{rab45}] \\ -k_{4to11,V189\cdot N1,j}[V_{189}\cdot N1_{rab45}]-k_{\deg r,V189\cdot N1,j}[V_{189}\cdot N1_{rab45}] \\ +k_{on,V189\cdot N1,j}[V_{189,rab45}]_{j}[N1_{rab45}]_{j}-k_{off,V189\cdot N1,j}[V_{189}\cdot N1_{rab45}]_{j} \\ -k_{on,(N1\cdot V189)\cdot R2,j}[N1\cdot V_{189,rab45}]_{j}[R2_{rab45}]_{j}+k_{off,(N1\cdot V189)\cdot R2,j}[N1\cdot V_{189}\cdot R2_{rab45}]_{j} \end{matrix}$$

$$\begin{matrix} d[P2\cdot N1_{rab45}]_{j}/dt=k_{\mathrm{int},P2\cdot N1,j}[P2\cdot N1]_{j}-k_{rec4,P2\cdot N1,j}[P2\cdot N1_{rab45}] \\ -k_{4to11,P2\cdot N1,j}[P2\cdot N1_{rab45}]-k_{\deg r,P2\cdot N1,j}[P2\cdot N1_{rab45}] \\ +k_{on,P2\cdot N1,j}[P2_{rab45}]_{j}[N1_{rab45}]_{j}-k_{off,P2\cdot N1,j}[P2\cdot N1_{rab45}]_{j} \end{matrix}$$

$$\begin{matrix} d[N1\cdot R1_{rab45}]_{j}/dt=k_{\mathrm{int},N1\cdot R1,j}[N1\cdot R1]_{j}-k_{rec4,N1\cdot R1,j}[N1\cdot R1_{rab45}] \\ -k_{4to11,N1\cdot R1,j}[N1\cdot R1_{rab45}]-k_{\deg r,N1\cdot R1,j}[N1\cdot R1_{rab45}] \\ +k_{on,N1\cdot R1,j}[N1_{rab45}]_{j}[R1_{rab45}]_{j}-k_{off,N1\cdot R1,j}[N1\cdot R1_{rab45}]_{j} \\ -k_{on,V121\cdot(N1\cdot R1),j}[V_{121,rab45}]_{j}[N1\cdot R1_{rab45}]_{j}+k_{off,V121\cdot(N1\cdot R1),j}[V_{121}\cdot R1\cdot N1_{rab45}]_{j} \\ -k_{on,P1\cdot(N1\cdot R1),j}[P1_{rab45}]_{j}[N1\cdot R1_{rab45}]_{j}+k_{off,P1\cdot(N1\cdot R1),j}[P1\cdot R1\cdot N1_{rab45}]_{j} \end{matrix}$$

$$\begin{matrix} d[N1\cdot sR1_{rab45}]_{j}/dt=k_{\mathrm{int},N1\cdot sR1,j}[N1\cdot sR1]_{j}-k_{rec4,N1\cdot sR1,j}[N1\cdot sR1_{rab45}] \\ -k_{4to11,N1\cdot sR1,j}[N1\cdot sR1_{rab45}]-k_{\deg r,N1\cdot sR1,j}[N1\cdot sR1_{rab45}] \\ +k_{on,N1\cdot sR1,j}[N1_{rab45}]_{j}[sR1_{rab45}]_{j}-k_{off,N1\cdot sR1,j}[N1\cdot sR1_{rab45}]_{j} \\ -k_{on,V121\cdot(N1\cdot sR1),j}[V_{121,rab45}]_{j}[N1\cdot sR1_{rab45}]_{j}+k_{off,V121\cdot(N1\cdot sR1),j}[V_{121}\cdot sR1\cdot N1_{rab45}]_{j} \\ -k_{on,P1\cdot(N1\cdot sR1),j}[P1_{rab45}]_{j}[N1\cdot sR1_{rab45}]_{j}+k_{off,P1\cdot(N1\cdot sR1),j}[P1\cdot sR1\cdot N1_{rab45}]_{j} \end{matrix}$$

$$\begin{matrix} d[N1\cdot V_{189}\cdot R2_{rab45}]_{j}/dt=k_{\mathrm{int},N1\cdot V189\cdot R2,j}[N1\cdot V_{189}\cdot R2]_{j}-k_{rec4,N1\cdot V189\cdot R2,j}[N1\cdot V_{189}\cdot R2_{rab45}] \\ -k_{4to11,N1\cdot V189\cdot R2,j}[N1\cdot V_{189}\cdot R2_{rab45}]-k_{\deg r,N1\cdot V189\cdot R2,j}[N1\cdot V_{189}\cdot R2_{rab45}] \\ +k_{on,R2\cdot(V189\cdot N1),j}[R2_{rab45}]_{j}[V_{189}\cdot N1_{rab45}]_{j}-k_{off,R2\cdot(N1\cdot V189),j}[N1\cdot V_{189}\cdot R2_{rab45}]_{j} \\ +k_{on,N1\cdot(V189\cdot R2),j}[N1_{rab45}]_{j}[V_{189}\cdot R2_{rab45}]_{j}-k_{off,N1\cdot(V189\cdot R2),j}[N1\cdot V_{189}\cdot R2_{rab45}]_{j} \end{matrix}$$

$$\begin{matrix} d[V_{121}\cdot R1\cdot N1_{rab45}]_{j}/dt=k_{\mathrm{int},V121\cdot R1\cdot N1,j}[V_{121}\cdot R1\cdot N1]_{j}-k_{rec4,V121\cdot R1\cdot N1,j}[V_{121}\cdot R1\cdot N1_{rab45}] \\ -k_{4to11,V121\cdot R1\cdot N1,j}[V_{121}\cdot R1\cdot N1_{rab45}]-k_{\deg r,V121\cdot R1\cdot N1,j}[V_{121}\cdot R1\cdot N1_{rab45}] \\ +k_{on,V121\cdot(R1\cdot N1),j}[V_{121,rab45}]_{j}[R1\cdot N1_{rab45}]_{j}-k_{off,V121\cdot(R1\cdot N1),j}[V_{121}\cdot R1\cdot N1_{rab45}]_{j} \\ +k_{on,(V121\cdot R1)\cdot N1,j}[V_{121}\cdot R1_{rab45}]_{j}[N1_{rab45}]_{j}-k_{off,(V121\cdot R1)\cdot N1,j}[V_{121}\cdot R1\cdot N1_{rab45}]_{j} \end{matrix}$$

$$\begin{matrix} d[P1\cdot R1\cdot N1_{rab45}]_{j}/dt=k_{\mathrm{int},P1\cdot R1\cdot N1,j}[P1\cdot R1\cdot N1]_{j}-k_{rec4,P1\cdot R1\cdot N1,j}[P1\cdot R1\cdot N1_{rab45}] \\ -k_{4to11,P1\cdot R1\cdot N1,j}[P1\cdot R1\cdot N1_{rab45}]-k_{\deg r,P1\cdot R1\cdot N1,j}[P1\cdot R1\cdot N1_{rab45}] \\ +k_{on,P1\cdot(R1\cdot N1),j}[P1_{rab45}]_{j}[R1\cdot N1_{rab45}]_{j}-k_{off,P1\cdot(R1\cdot N1),j}[P1\cdot R1\cdot N1_{rab45}]_{j} \\ +k_{on,(P1\cdot R1)\cdot N1,j}[P1\cdot R1_{rab45}]_{j}[N1_{rab45}]_{j}-k_{off,(P1\cdot R1)\cdot N1,j}[P1\cdot R1\cdot N1_{rab45}]_{j} \end{matrix}$$

$$\begin{matrix} d[V_{121}\cdot sR1\cdot N1_{rab45}]_{j}/dt=k_{\mathrm{int},V121\cdot sR1\cdot N1,j}[V_{121}\cdot sR1\cdot N1]_{j}-k_{rec4,V121\cdot sR1\cdot N1,j}[V_{121}\cdot sR1\cdot N1_{rab45}] \\ -k_{4to11,V121\cdot sR1\cdot N1,j}[V_{121}\cdot sR1\cdot N1_{rab45}]-k_{\deg r,V121\cdot sR1\cdot N1,j}[V_{121}\cdot sR1\cdot N1_{rab45}] \\ +k_{on,V121\cdot(sR1\cdot N1),j}[V_{121,rab45}]_{j}[sR1\cdot N1_{rab45}]_{j}-k_{off,V121\cdot(sR1\cdot N1),j}[V_{121}\cdot sR1\cdot N1_{rab45}]_{j} \\ +k_{on,(V121\cdot sR1)\cdot N1,j}[V_{121}\cdot sR1_{rab45}]_{j}[N1_{rab45}]_{j}-k_{off,(V121\cdot sR1)\cdot N1,j}[V_{121}\cdot sR1\cdot N1_{rab45}]_{j} \end{matrix}$$

$$\begin{matrix} d[P1\cdot sR1\cdot N1_{rab45}]_{j}/dt=k_{\mathrm{int},P1\cdot sR1\cdot N1,j}[P1\cdot sR1\cdot N1]_{j}-k_{rec4,P1\cdot sR1\cdot N1,j}[P1\cdot sR1\cdot N1_{rab45}] \\ -k_{4to11,P1\cdot sR1\cdot N1,j}[P1\cdot sR1\cdot N1_{rab45}]-k_{\deg r,P1\cdot sR1\cdot N1,j}[P1\cdot sR1\cdot N1_{rab45}] \\ +k_{on,P1\cdot(sR1\cdot N1),j}[P1_{rab45}]_{j}[sR1\cdot N1_{rab45}]_{j}-k_{off,P1\cdot(sR1\cdot N1),j}[P1\cdot sR1\cdot N1_{rab45}]_{j} \\ +k_{on,(P1\cdot sR1)\cdot N1,j}[P1\cdot sR1_{rab45}]_{j}[N1_{rab45}]_{j}-k_{off,(P1\cdot sR1)\cdot N1,j}[P1\cdot sR1\cdot N1_{rab45}]_{j} \end{matrix}$$

### Rab11 Recycling Endosomes

$$\begin{matrix} d[R1_{rab11}]_{j}/dt=k_{4to11,R1,j}[R1_{rab45}]-k_{rec11,R1,j}[R1_{rab11}] \\ -k_{on,V165\cdot R1,j}[V_{165,rab11}]_{j}[R1_{rab11}]_{j}+k_{off,V165\cdot R1,j}[V_{165}\cdot R1_{rab11}]_{j} \\ -k_{on,V189\cdot R1,j}[V_{189,rab11}]_{j}[R1_{rab11}]_{j}+k_{off,V189\cdot R1,j}[V_{189}\cdot R1_{rab11}]_{j} \\ -k_{on,V121\cdot R1,j}[V_{121,rab45}]_{j}[R1_{rab11}]_{j}+k_{off,V121\cdot R1,j}[V_{121}\cdot R1_{rab11}]_{j} \\ -k_{on,P1\cdot R1,j}[P1_{rab11}]_{j}[R1_{rab11}]_{j}+k_{off,P1\cdot R1,j}[P1\cdot R1_{rab11}]_{j} \\ -k_{on,P2\cdot R1,j}[P2_{rab11}]_{j}[R1_{rab11}]_{j}+k_{off,P2\cdot R1,j}[P2\cdot R1_{rab11}]_{j} \\ -k_{on,N1\cdot R1,j}[N1_{rab11}]_{j}[R1_{rab11}]_{j}+k_{off,N1\cdot R1,j}[N1\cdot R1_{rab11}]_{j} \end{matrix}$$

$$\begin{matrix} d[R2_{rab11}]_{j}/dt=k_{4to11,R2,j}[R2_{rab45}]-k_{rec11,R2,j}[R2_{rab11}] \\ -k_{on,V165\cdot R2,j}[V_{165,rab11}]_{j}[R2_{rab11}]_{j}+k_{off,V165\cdot R2,j}[V_{165}\cdot R2_{rab11}]_{j} \\ -k_{on,V189\cdot R2,j}[V_{189,rab11}]_{j}[R2_{rab11}]_{j}+k_{off,V189\cdot R2,j}[V_{189}\cdot R2_{rab11}]_{j} \\ -k_{on,V121\cdot R2,j}[V_{121,rab11}]_{j}[R2_{rab11}]_{j}+k_{off,V121\cdot R2,j}[V_{121}\cdot R2_{rab11}]_{j} \\ -k_{on,(N1\cdot V165)\cdot R2,j}[N1\cdot V_{165,rab11}]_{j}[R2_{rab11}]_{j}+k_{off,(N1\cdot V165)\cdot R2,j}[N1\cdot V_{165}\cdot R2_{rab11}]_{j} \\ -k_{on,(N1\cdot V189)\cdot R2,j}[N1\cdot V_{189,rab11}]_{j}[R2_{rab11}]_{j}+k_{off,(N1\cdot V189)\cdot R2,j}[N1\cdot V_{189}\cdot R2_{rab11}]_{j} \end{matrix}$$

$$\begin{matrix} d[V_{165}\cdot R1_{rab11}]_{j}/dt=k_{4to11,V165\cdot R1,j}[V_{165}\cdot R1_{rab45}]-k_{rec11,V165\cdot R1,j}[V_{165}\cdot R1_{rab11}] \\ +k_{on,V165\cdot R1,j}[V_{165,rab11}]_{j}[R1_{rab11}]_{j}-k_{off,V165\cdot R1,j}[V_{165}\cdot R1_{rab11}]_{j} \end{matrix}$$

$$\begin{matrix} d[V_{189}\cdot R1_{rab11}]_{j}/dt=k_{4to11,V189\cdot R1,j}[V_{189}\cdot R1_{rab45}]-k_{rec11,V189\cdot R1,j}[V_{189}\cdot R1_{rab11}] \\ +k_{on,V189\cdot R1,j}[V_{189,rab11}]_{j}[R1_{rab11}]_{j}-k_{off,V189\cdot R1,j}[V_{189}\cdot R1_{rab11}]_{j} \end{matrix}$$

$$\begin{matrix} d[V_{121}\cdot R1_{rab11}]_{j}/dt=k_{4to11,V121\cdot R1,j}[V_{121}\cdot R1_{rab45}]-k_{rec11,V121\cdot R1,j}[V_{121}\cdot R1_{rab11}] \\ +k_{on,V121\cdot R1,j}[V_{121,rab11}]_{j}[R1_{rab11}]_{j}-k_{off,V121\cdot R1,j}[V_{121}\cdot R1_{rab11}]_{j} \\ -k_{on,(V121\cdot R1)\cdot N1,j}[V_{121}\cdot R1_{rab11}]_{j}[N1_{rab11}]_{j}+k_{off,(V121\cdot R1)\cdot N1,j}[V_{121}\cdot R1\cdot N1_{rab11}]_{j} \end{matrix}$$

$$\begin{matrix} d[P1\cdot R1_{rab11}]_{j}/dt=k_{4to11,P1\cdot R1,j}[P1\cdot R1_{rab45}]-k_{rec11,P1\cdot R1,j}[P1\cdot R1_{rab11}] \\ +k_{on,P1\cdot R1,j}[P1_{rab11}]_{j}[R1_{rab11}]_{j}-k_{off,P1\cdot R1,j}[P1\cdot R1_{rab11}]_{j} \\ -k_{on,(P1\cdot R1)\cdot N1,j}[P1\cdot R1_{rab11}]_{j}[N1_{rab11}]_{j}+k_{off,(P1\cdot R1)\cdot N1,j}[P1\cdot R1\cdot N1_{rab11}]_{j} \end{matrix}$$

$$\begin{matrix} d[P2\cdot R1_{rab11}]_{j}/dt=k_{4to11,P2\cdot R1,j}[P2\cdot R1_{rab45}]-k_{rec11,P2\cdot R1,j}[P2\cdot R1_{rab11}] \\ +k_{on,P2\cdot R1,j}[P2_{rab11}]_{j}[R1_{rab11}]_{j}-k_{off,P2\cdot R1,j}[P2\cdot R1_{rab11}]_{j} \end{matrix}$$

$$d[V_{165}\cdot sR1_{rab11}]_{j}/dt=k_{on,V165\cdot sR1,j}[V_{165,rab11}]_{j}[sR1_{rab11}]_{j}-k_{off,V165\cdot sR1,j}[V_{165}\cdot sR1_{rab11}]_{j}$$

$$\begin{matrix} d[V_{121}\cdot sR1_{rab11}]_{j}/dt=k_{on,V121\cdot sR1,j}[V_{121,rab11}]_{j}[sR1_{rab11}]_{j}-k_{off,V121\cdot sR1,j}[V_{121}\cdot sR1_{rab11}]_{j} \\ -k_{on,(V121\cdot sR1)\cdot N1,j}[V_{121}\cdot sR1_{rab11}]_{j}[N1_{rab11}]_{j}+k_{off,(V121\cdot sR1)\cdot N1,j}[V_{121}\cdot sR1\cdot N1_{rab11}]_{j} \end{matrix}$$

$$d[V_{189}\cdot sR1_{rab11}]_{j}/dt=k_{on,V189\cdot sR1,j}[V_{189,rab11}]_{j}[sR1_{rab11}]_{j}-k_{off,V189\cdot sR1,j}[V_{189}\cdot sR1_{rab11}]_{j}$$

$$\begin{matrix} d[P1\cdot sR1_{rab11}]_{j}/dt=k_{on,P1\cdot sR1,j}[P1_{rab11}]_{j}[sR1_{rab11}]_{j}-k_{off,P1\cdot sR1,j}[P1\cdot sR1_{rab11}]_{j} \\ -k_{on,(P1\cdot sR1)\cdot N1,j}[P1\cdot sR1_{rab11}]_{j}[N1_{rab11}]_{j}+k_{off,(P1\cdot sR1)\cdot N1,j}[P1\cdot sR1\cdot N1_{rab11}]_{j} \end{matrix}$$

$$d[P2\cdot sR1_{rab11}]_{j}/dt=k_{on,P2\cdot sR1,j}[P2_{rab11}]_{j}[sR1_{rab11}]_{j}-k_{off,P2\cdot sR1,j}[P2\cdot sR1_{rab11}]_{j}$$

$$\begin{matrix} d[V_{165}\cdot R2_{rab11}]_{j}/dt=k_{4to11,V165\cdot R2,j}[V_{165}\cdot R2_{rab45}]-k_{rec11,V165\cdot R2,j}[V_{165}\cdot R2_{rab11}] \\ +k_{on,V165\cdot R2,j}[V_{165,rab45}]_{j}[R2_{rab11}]_{j}-k_{off,V165\cdot R2,j}[V_{165}\cdot R2_{rab11}]_{j} \\ -k_{on,N1\cdot(V165\cdot R2),j}[N1_{rab11}]_{j}[V_{165}\cdot R2_{rab11}]_{j}+k_{off,N1\cdot(V165\cdot R2),j}[N1\cdot V_{165}\cdot R2_{rab11}]_{j} \end{matrix}$$

$$\begin{matrix} d[V_{189}\cdot R2_{rab11}]_{j}/dt=k_{4to11,V189\cdot R2,j}[V_{189}\cdot R2_{rab45}]-k_{rec11,V189\cdot R2,j}[V_{189}\cdot R2_{rab11}] \\ +k_{on,V189\cdot R2,j}[V_{189,rab11}]_{j}[R2_{rab11}]_{j}-k_{off,V189\cdot R2,j}[V_{189}\cdot R2_{rab11}]_{j} \\ -k_{on,N1\cdot(V189\cdot R2),j}[N1_{rab11}]_{j}[V_{189}\cdot R2_{rab11}]_{j}+k_{off,N1\cdot(V189\cdot R2),j}[N1\cdot V_{189}\cdot R2_{rab11}]_{j} \end{matrix}$$

$$\begin{matrix} d[V_{121}\cdot R2_{rab11}]_{j}/dt=k_{4to11,V121\cdot R2,j}[V_{121}\cdot R2_{rab45}]-k_{rec11,V121\cdot R2,j}[V_{121}\cdot R2_{rab11}] \\ +k_{on,V121\cdot R2,j}[V_{121,rab11}]_{j}[R2_{rab11}]_{j}-k_{off,V121\cdot R2,j}[V_{121}\cdot R2_{rab11}]_{j} \end{matrix}$$

$$\begin{matrix} d[V_{165}\cdot N1_{rab11}]_{j}/dt=k_{4to11,V165\cdot N1,j}[V_{165}\cdot N1_{rab45}]-k_{rec11,V165\cdot N1,j}[V_{165}\cdot N1_{rab11}] \\ +k_{on,V165\cdot N1,j}[V_{165,rab11}]_{j}[N1_{rab11}]_{j}-k_{off,V165\cdot N1,j}[V_{165}\cdot N1_{rab11}]_{j} \\ -k_{on,(N1\cdot V165)\cdot R2,j}[N1\cdot V_{165,rab11}]_{j}[R2_{rab11}]_{j}+k_{off,(N1\cdot V165)\cdot R2,j}[N1\cdot V_{165}\cdot R2_{rab11}]_{j} \end{matrix}$$

$$\begin{matrix} d[V_{189}\cdot N1_{rab11}]_{j}/dt=k_{4to11,V189\cdot N1,j}[V_{189}\cdot N1_{rab11}]-k_{rec11,V189\cdot N1,j}[V_{189}\cdot N1_{rab11}] \\ +k_{on,V189\cdot N1,j}[V_{189,rab11}]_{j}[N1_{rab11}]_{j}-k_{off,V189\cdot N1,j}[V_{189}\cdot N1_{rab11}]_{j} \\ -k_{on,(N1\cdot V189)\cdot R2,j}[N1\cdot V_{189,rab11}]_{j}[R2_{rab11}]_{j}+k_{off,(N1\cdot V189)\cdot R2,j}[N1\cdot V_{189}\cdot R2_{rab11}]_{j} \end{matrix}$$

$$\begin{matrix} d[P2\cdot N1_{rab11}]_{j}/dt=k_{4to11,P2\cdot N1,j}[P2\cdot N1_{rab45}]-k_{rec11,P2\cdot N1,j}[P2\cdot N1_{rab11}] \\ +k_{on,P2\cdot N1,j}[P2_{rab11}]_{j}[N1_{rab11}]_{j}-k_{off,P2\cdot N1,j}[P2\cdot N1_{rab11}]_{j} \end{matrix}$$

$$\begin{matrix} d[N1\cdot R1_{rab11}]_{j}/dt=k_{4to11,N1\cdot R1,j}[N1\cdot R1_{rab45}]-k_{rec11,N1\cdot R1,j}[N1\cdot R1_{rab11}] \\ +k_{on,N1\cdot R1,j}[N1_{rab11}]_{j}[R1_{rab11}]_{j}-k_{off,N1\cdot R1,j}[N1\cdot R1_{rab11}]_{j} \\ -k_{on,V121\cdot(N1\cdot R1),j}[V_{121,rab11}]_{j}[N1\cdot R1_{rab11}]_{j}+k_{off,V121\cdot(N1\cdot R1),j}[V_{121}\cdot R1\cdot N1_{rab11}]_{j} \\ -k_{on,P1\cdot(N1\cdot R1),j}[P1_{rab11}]_{j}[N1\cdot R1_{rab11}]_{j}+k_{off,P1\cdot(N1\cdot R1),j}[P1\cdot R1\cdot N1_{rab11}]_{j} \end{matrix}$$

$$\begin{matrix} d[N1\cdot sR1_{rab11}]_{j}/dt=k_{4to11,N1\cdot sR1,j}[N1\cdot sR1_{rab45}]-k_{rec11,N1\cdot sR1,j}[N1\cdot sR1_{rab11}] \\ +k_{on,N1\cdot sR1,j}[N1_{rab11}]_{j}[sR1_{rab11}]_{j}-k_{off,N1\cdot sR1,j}[N1\cdot sR1_{rab11}]_{j} \\ -k_{on,V121\cdot(N1\cdot sR1),j}[V_{121,rab11}]_{j}[N1\cdot sR1_{rab11}]_{j}+k_{off,V121\cdot(N1\cdot sR1),j}[V_{121}\cdot sR1\cdot N1_{rab11}]_{j} \\ -k_{on,P1\cdot(N1\cdot sR1),j}[P1_{rab11}]_{j}[N1\cdot sR1_{rab11}]_{j}+k_{off,P1\cdot(N1\cdot sR1),j}[P1\cdot sR1\cdot N1_{rab11}]_{j} \end{matrix}$$

$$\begin{matrix} d[N1\cdot V_{165}\cdot R2_{rab11}]_{j}/dt=k_{4to11,N1\cdot V165\cdot R2,j}[N1\cdot V_{165}\cdot R2_{rab45}]-k_{rec11,N1\cdot V165\cdot R2,j}[N1\cdot V_{165}\cdot R2_{rab11}] \\ +k_{on,R2\cdot(N1\cdot V165),j}[R2_{rab11}]_{j}[V_{165}\cdot N1_{rab11}]_{j}-k_{off,R2\cdot(N1\cdot V165),j}[N1\cdot V_{165}\cdot R2_{rab11}]_{j} \\ +k_{on,N1\cdot(V165\cdot R2),j}[N1_{rab11}]_{j}[V_{165}\cdot R2_{rab11}]_{j}-k_{off,N1\cdot(V165\cdot R2),j}[N1\cdot V_{165}\cdot R2_{rab11}]_{j} \end{matrix}$$

$$\begin{matrix} d[N1\cdot V_{189}\cdot R2_{rab11}]_{j}/dt=k_{4to11,N1\cdot V189\cdot R2,j}[N1\cdot V_{189}\cdot R2_{rab45}]-k_{rec11,N1\cdot V189\cdot R2,j}[N1\cdot V_{189}\cdot R2_{rab11}] \\ +k_{on,R2\cdot(V189\cdot N1),j}[R2_{rab11}]_{j}[V_{189}\cdot N1_{rab11}]_{j}-k_{off,R2\cdot(N1\cdot V189),j}[N1\cdot V_{189}\cdot R2_{rab11}]_{j} \\ +k_{on,N1\cdot(V189\cdot R2),j}[N1_{rab11}]_{j}[V_{189}\cdot R2_{rab11}]_{j}-k_{off,N1\cdot(V189\cdot R2),j}[N1\cdot V_{189}\cdot R2_{rab11}]_{j} \end{matrix}$$

$$\begin{matrix} d[V_{121}\cdot R1\cdot N1_{rab11}]_{j}/dt=k_{4to11,V121\cdot R1\cdot N1,j}[V_{121}\cdot R1\cdot N1_{rab45}]-k_{rec11,V121\cdot R1\cdot N1,j}[V_{121}\cdot R1\cdot N1_{rab11}] \\ +k_{on,V121\cdot(R1\cdot N1),j}[V_{121,rab11}]_{j}[R1\cdot N1_{rab11}]_{j}-k_{off,V121\cdot(R1\cdot N1),j}[V_{121}\cdot R1\cdot N1_{rab11}]_{j} \\ +k_{on,(V121\cdot R1)\cdot N1,j}[V_{121}\cdot R1_{rab11}]_{j}[N1_{rab11}]_{j}-k_{off,(V121\cdot R1)\cdot N1,j}[V_{121}\cdot R1\cdot N1_{rab11}]_{j} \end{matrix}$$

$$\begin{matrix} d[P1\cdot R1\cdot N1_{rab11}]_{j}/dt=k_{4to11,P1\cdot R1\cdot N1,j}[P1\cdot R1\cdot N1_{rab45}]-k_{rec11,P1\cdot R1\cdot N1,j}[P1\cdot R1\cdot N1_{rab11}] \\ +k_{on,P1\cdot(R1\cdot N1),j}[P1_{rab11}]_{j}[R1\cdot N1_{rab11}]_{j}-k_{off,P1\cdot(R1\cdot N1),j}[P1\cdot R1\cdot N1_{rab11}]_{j} \\ +k_{on,(P1\cdot R1)\cdot N1,j}[P1\cdot R1_{rab11}]_{j}[N1_{rab11}]_{j}-k_{off,(P1\cdot R1)\cdot N1,j}[P1\cdot R1\cdot N1_{rab11}]_{j} \end{matrix}$$

$$\begin{matrix} d[V_{121}\cdot sR1\cdot N1_{rab11}]_{j}/dt=k_{4to11,V121\cdot sR1\cdot N1,j}[V_{121}\cdot sR1\cdot N1_{rab45}]-k_{\deg r,V121\cdot sR1\cdot N1,j}[V_{121}\cdot sR1\cdot N1_{rab11}] \\ +k_{on,V121\cdot(sR1\cdot N1),j}[V_{121,rab11}]_{j}[sR1\cdot N1_{rab11}]_{j}-k_{off,V121\cdot(sR1\cdot N1),j}[V_{121}\cdot sR1\cdot N1_{rab11}]_{j} \\ +k_{on,(V121\cdot sR1)\cdot N1,j}[V_{121}\cdot sR1_{rab11}]_{j}[N1_{rab11}]_{j}-k_{off,(V121\cdot sR1)\cdot N1,j}[V_{121}\cdot sR1\cdot N1_{rab11}]_{j} \end{matrix}$$

$$\begin{matrix} d[P1\cdot sR1\cdot N1_{rab11}]_{j}/dt=k_{4to11,P1\cdot sR1\cdot N1,j}[P1\cdot sR1\cdot N1_{rab45}]-k_{rec11,P1\cdot sR1\cdot N1,j}[P1\cdot sR1\cdot N1_{rab11}] \\ +k_{on,P1\cdot(sR1\cdot N1),j}[P1_{rab11}]_{j}[sR1\cdot N1_{rab11}]_{j}-k_{off,P1\cdot(sR1\cdot N1),j}[P1\cdot sR1\cdot N1_{rab11}]_{j} \\ +k_{on,(P1\cdot sR1)\cdot N1,j}[P1\cdot sR1_{rab11}]_{j}[N1_{rab11}]_{j}-k_{off,(P1\cdot sR1)\cdot N1,j}[P1\cdot sR1\cdot N1_{rab11}]_{j} \end{matrix}$$

Interstitial Fluid. These 11 equations describe the free species found in the interstitial fluid in tissues, including free VEGF, PlGF, and sR1, and complexes of sR1 with ligands. q_X_ is the constant secretion of VEGF or PlGF isoforms from myocytes (molecules/myonuclear domain/s), or of sR1 from endothelial cells (molecules/EC/s), as given in **Table 4**, and converted into moles/cm^3^ tissue**.** All molecular species in the interstitial fluid can be transported into the blood via lymphatic drainage (k_L_ in cm^3^/s), or moved between the blood and tissue via bi-directional vascular permeability (k_p_ in cm/s). (See **Table S10** for transport parameter values.) Vascular permeability depends on the total abluminal EC surface area, S_jB_ (cm^2^). The endothelial cell surface recruitment factor γ is always one in this study, but can be used to account for changes in transport as a result of altered perfusion or vasodilation. As detailed above, U represents a volume, while K_Av_ is the fraction of the volume that is available. These geometric factors are included to account for the relevant volumes in the tissue and blood for exchange of molecular species; when one molecule is transported between tissue j and the blood, the concentration changes in j and the blood depend on the respective volumes.

$$\begin{matrix} d[V_{165}]_{j}/dt=q_{V165,j}-\frac{k_{L,j}}{U_{j}}\frac{[V_{165}]_{j}}{K_{Av,j}}+\frac{\gamma_{j}\cdot S_{jB}}{U_{j}}\cdot\left(k_{p,V}^{B\to j}\frac{[V_{165}]_{B}}{K_{AV,B}}-k_{p,V}^{j\to B}\frac{[V_{165}]_{j}}{K_{AV,j}}\right) \\ -k_{on,V165\cdot M,j}[V_{165}]_{j}[M_{EBM}]_{j}+k_{off,V165\cdot M,j}[V_{165}\cdot M_{EBM}]_{j} \\ -k_{on,V165\cdot M,j}[V_{165}]_{j}[M_{ECM}]_{j}+k_{off,V165\cdot M,j}[V_{165}\cdot M_{ECM}]_{j} \\ -k_{on,V165\cdot M,j}[V_{165}]_{j}[M_{PBM}]_{j}+k_{off,V165\cdot M,j}[V_{165}\cdot M_{PBM}]_{j} \\ -k_{on,V165\cdot N1,j}[V_{165}]_{j}[N1]_{j}+k_{off,V165\cdot N1,j}[V_{165}\cdot N1]_{j} \\ -k_{on,V165\cdot R2,j}[V_{165}]_{j}[R2]_{j}+k_{off,V165\cdot R2,j}[V_{165}\cdot R2]_{j} \\ -k_{on,V165\cdot R1,j}[V_{165}]_{j}[R1]_{j}+k_{off,V165\cdot R1,j}[V_{165}\cdot R1]_{j} \\ -k_{on,V165\cdot sR1,j}[V_{165}]_{j}[sR1]_{j}+k_{off,V165\cdot sR1,j}[V_{165}\cdot sR1]_{j} \end{matrix}$$

$$\begin{matrix} d[V_{189}]_{j}/dt=q_{V189,j}-\frac{k_{L,j}}{U_{j}}\frac{[V_{189}]_{j}}{K_{Av,j}}+\frac{\gamma_{j}\cdot S_{jB}}{U_{j}}\cdot\left(k_{p,V}^{B\to j}\frac{[V_{189}]_{B}}{K_{AV,B}}-k_{p,V}^{j\to B}\frac{[V_{189}]_{j}}{K_{AV,j}}\right) \\ -k_{on,V189\cdot M,j}[V_{189}]_{j}[M_{EBM}]_{j}+k_{off,V189\cdot M,j}[V_{189}\cdot M_{EBM}]_{j} \\ -k_{on,V189\cdot M,j}[V_{189}]_{j}[M_{ECM}]_{j}+k_{off,V189\cdot M,j}[V_{189}\cdot M_{ECM}]_{j} \\ -k_{on,V189\cdot M,j}[V_{189}]_{j}[M_{PBM}]_{j}+k_{off,V189\cdot M,j}[V_{189}\cdot M_{PBM}]_{j} \\ -k_{on,V189\cdot N1,j}[V_{189}]_{j}[N1]_{j}+k_{off,V189\cdot N1,j}[V_{189}\cdot N1]_{j} \\ -k_{on,V189\cdot R2,j}[V_{189}]_{j}[R2]_{j}+k_{off,V189\cdot R2,j}[V_{189}\cdot R2]_{j} \\ -k_{on,V189\cdot R1,j}[V_{189}]_{j}[R1]_{j}+k_{off,V189\cdot R1,j}[V_{189}\cdot R1]_{j} \\ -k_{on,V189\cdot sR1,j}[V_{189}]_{j}[sR1]_{j}+k_{off,V189\cdot sR1,j}[V_{189}\cdot sR1]_{j} \end{matrix}$$

$$\begin{matrix} d[P1]_{j}/dt=q_{P1,j}-\frac{k_{L,j}}{U_{j}}\frac{[P1]_{j}}{K_{Av,j}}+\frac{\gamma_{j}\cdot S_{jB}}{U_{j}}\cdot\left(k_{p,P}^{B\to j}\frac{[P1]_{B}}{K_{AV,B}}-k_{p,P}^{j\to B}\frac{[P]_{j}}{K_{AV,j}}\right) \\ -k_{on,P1\cdot R1,j}[P1]_{j}[R1]_{j}+k_{off,P1\cdot R1,j}[P1\cdot R1]_{j} \\ -k_{on,P1\cdot(R1\cdot N1),j}[P1]_{j}[R1\cdot N1]_{j}+k_{off,P1\cdot(R1\cdot N1),j}[P1\cdot R1\cdot N1]_{j} \\ -k_{on,P1\cdot sR1,j}[P1]_{j}[sR1]_{j}+k_{off,P1\cdot sR1,j}[P1\cdot sR1]_{j} \\ -k_{on,P1\cdot(sR1\cdot N1),j}[P1]_{j}[sR1\cdot N1]_{j}+k_{off,P1\cdot(sR1\cdot N1),j}[P1\cdot sR1\cdot N1]_{j} \\ -k_{on,P1\cdot(sR1\cdot M),j}[P1]_{j}[sR1\cdot M_{EBM}]_{j}+k_{off,P1\cdot(sR1\cdot M),j}[P1\cdot sR1\cdot M_{EBM}]_{j} \\ -k_{on,P1\cdot(sR1\cdot M),j}[P1]_{j}[sR1\cdot M_{ECM}]_{j}+k_{off,P1\cdot(sR1\cdot M),j}[P1\cdot sR1\cdot M_{ECM}]_{j} \\ -k_{on,P1\cdot(sR1\cdot M),j}[P1]_{j}[sR1\cdot M_{PBM}]_{j}+k_{off,P1\cdot(sR1\cdot M),j}[P1\cdot sR1\cdot M_{PBM}]_{j} \end{matrix}$$

$$\begin{matrix} d[P2]_{j}/dt=q_{P2,j}-\frac{k_{L,j}}{U_{j}}\frac{[P2]_{j}}{K_{Av,j}}+\frac{\gamma_{j}\cdot S_{jB}}{U_{j}}\cdot\left(k_{p,P}^{B\to j}\frac{[P2]_{B}}{K_{AV,B}}-k_{p,P}^{j\to B}\frac{[P2]_{j}}{K_{AV,j}}\right) \\ -k_{on,P2\cdot M,j}[P2]_{j}[M_{EBM}]_{j}+k_{off,P2\cdot M,j}[P2\cdot M_{EBM}]_{j} \\ -k_{on,P2\cdot M,j}[P2]_{j}[M_{ECM}]_{j}+k_{off,P2\cdot M,j}[P2\cdot M_{ECM}]_{j} \\ -k_{on,P2\cdot M,j}[P2]_{j}[M_{PBM}]_{j}+k_{off,P2\cdot M,j}[P2\cdot M_{PBM}]_{j} \\ -k_{on,P2\cdot N1,j}[P2]_{j}[N1]_{j}+k_{off,P2\cdot N1,j}[P2\cdot N1]_{j} \\ -k_{on,P2\cdot R1,j}[P2]_{j}[R1]_{j}+k_{off,P2\cdot R1,j}[P2\cdot R1]_{j} \\ -k_{on,P2\cdot sR1,j}[P2]_{j}[sR1]_{j}+k_{off,P2\cdot sR1,j}[P2\cdot sR1]_{j} \end{matrix}$$

$$\begin{matrix} d[V_{165}\cdot sR1]_{j}/dt=-\frac{k_{L,j}}{U_{j}}\frac{[V_{165}\cdot sR1]_{j}}{K_{Av,j}}+\frac{\gamma_{j}\cdot S_{jB}}{U_{j}}\cdot\left(k_{p,sR1}^{B\to j}\frac{[V_{165}\cdot sR1]_{B}}{K_{AV,B}}-k_{p,sR1}^{j\to B}\frac{[V_{165}\cdot sR1]_{j}}{K_{AV,j}}\right) \\ +k_{on,V165\cdot sR1,j}[V_{165}]_{j}[sR1]_{j}-k_{off,V165\cdot sR1,j}[V_{165}\cdot sR1]_{j} \\ -k_{on,M\cdot(V165\cdot sR1),j}[M_{EBM}]_{j}[V_{165}\cdot sR1]_{j}+k_{off,M\cdot(V165\cdot sR1),j}[M_{EBM}\cdot V_{165}\cdot sR1]_{j} \\ -k_{on,M\cdot(V165\cdot sR1),j}[M_{ECM}]_{j}[V_{165}\cdot sR1]_{j}+k_{off,M\cdot(V165\cdot sR1),j}[M_{ECM}\cdot V_{165}\cdot sR1]_{j} \\ -k_{on,M\cdot(V165\cdot sR1),j}[M_{PBM}]_{j}[V_{165}\cdot sR1]_{j}+k_{off,M\cdot(V165\cdot sR1),j}[M_{PBM}\cdot V_{165}\cdot sR1]_{j} \end{matrix}$$

$$\begin{matrix} d[V_{189}\cdot sR1]_{j}/dt=-\frac{k_{L,j}}{U_{j}}\frac{[V_{189}\cdot sR1]_{j}}{K_{Av,j}}+\frac{\gamma_{j}\cdot S_{jB}}{U_{j}}\cdot\left(k_{p,sR1}^{B\to j}\frac{[V_{189}\cdot sR1]_{B}}{K_{AV,B}}-k_{p,sR1}^{j\to B}\frac{[V_{189}\cdot sR1]_{j}}{K_{AV,j}}\right) \\ +k_{on,V189\cdot sR1,j}[V_{189}]_{j}[sR1]_{j}-k_{off,V189\cdot sR1,j}[V_{189}\cdot sR1]_{j} \\ -k_{on,M\cdot(V189\cdot sR1),j}[M_{EBM}]_{j}[V_{189}\cdot sR1]_{j}+k_{off,M\cdot(V189\cdot sR1),j}[M_{EBM}\cdot V_{189}\cdot sR1]_{j} \\ -k_{on,M\cdot(V189\cdot sR1),j}[M_{ECM}]_{j}[V_{189}\cdot sR1]_{j}+k_{off,M\cdot(V189\cdot sR1),j}[M_{ECM}\cdot V_{189}\cdot sR1]_{j} \\ -k_{on,M\cdot(V189\cdot sR1),j}[M_{PBM}]_{j}[V_{189}\cdot sR1]_{j}+k_{off,M\cdot(V189\cdot sR1),j}[M_{PBM}\cdot V_{189}\cdot sR1]_{j} \end{matrix}$$

$$\begin{matrix} d[V_{121}\cdot sR1]_{j}/dt=-\frac{k_{L,j}}{U_{j}}\frac{[V_{121}\cdot sR1]_{j}}{K_{Av,j}}+\frac{\gamma_{j}\cdot S_{jB}}{U_{j}}\cdot\left(k_{p,sR1}^{B\to j}\frac{[V_{121}\cdot sR1]_{B}}{K_{AV,B}}-k_{p,sR1}^{j\to B}\frac{[V_{121}\cdot sR1]_{j}}{K_{AV,j}}\right) \\ +k_{on,V121\cdot sR1,j}[V_{121}]_{j}[sR1]_{j}-k_{off,V121\cdot sR1,j}[V_{121}\cdot sR1]_{j} \\ -k_{on,(M\cdot sR1)\cdot V121,j}[M_{EBM}\cdot sR1]_{j}[V_{121}]_{j}+k_{off,(M\cdot sR1)\cdot V121,j}[M_{EBM}\cdot sR1\cdot V_{121}]_{j} \\ -k_{on,(M\cdot sR1)\cdot V121,j}[M_{ECM}\cdot sR1]_{j}[V_{121}]_{j}+k_{off,(M\cdot sR1)\cdot V121,j}[M_{ECM}\cdot sR1\cdot V_{121}]_{j} \\ -k_{on,(M\cdot sR1)\cdot V121,j}[M_{PBM}\cdot sR1]_{j}[V_{121}]_{j}+k_{off,(M\cdot sR1)\cdot V121,j}[M_{PBM}\cdot sR1\cdot V_{121}]_{j} \end{matrix}$$

$$\begin{matrix} d[P1\cdot sR1]_{j}/dt=-\frac{k_{L,j}}{U_{j}}\frac{[P1\cdot sR1]_{j}}{K_{Av,j}}+\frac{\gamma_{j}\cdot S_{jB}}{U_{j}}\cdot\left(k_{p,sR1}^{B\to j}\frac{[P1\cdot sR1]_{B}}{K_{AV,B}}-k_{p,sR1}^{j\to B}\frac{[P1\cdot sR1]_{j}}{K_{AV,j}}\right) \\ +k_{on,P1\cdot sR1,j}[P1]_{j}[sR1]_{j}-k_{off,P1\cdot sR1,j}[P1\cdot sR1]_{j} \\ -k_{on,(M\cdot sR1)\cdot P1,j}[M_{EBM}\cdot sR1]_{j}[P1]_{j}+k_{off,(M\cdot sR1)\cdot P1,j}[M_{EBM}\cdot sR1\cdot P1]_{j} \\ -k_{on,(M\cdot sR1)\cdot P1,j}[M_{ECM}\cdot sR1]_{j}[P1]_{j}+k_{off,(M\cdot sR1)\cdot P1,j}[M_{ECM}\cdot sR1\cdot P1]_{j} \\ -k_{on,(M\cdot sR1)\cdot P1,j}[M_{PBM}\cdot sR1]_{j}[P1]_{j}+k_{off,(M\cdot sR1)\cdot P1,j}[M_{PBM}\cdot sR1\cdot P1]_{j} \end{matrix}$$

$$\begin{matrix} d[P2\cdot sR1]_{j}/dt=-\frac{k_{L,j}}{U_{j}}\frac{[P2\cdot sR1]_{j}}{K_{Av,j}}+\frac{\gamma_{j}\cdot S_{jB}}{U_{j}}\cdot\left(k_{p,sR1}^{B\to j}\frac{[P2\cdot sR1]_{B}}{K_{AV,B}}-k_{p,sR1}^{j\to B}\frac{[P2\cdot sR1]_{j}}{K_{AV,j}}\right) \\ +k_{on,P2\cdot sR1,j}[P2]_{j}[sR1]_{j}-k_{off,P2\cdot sR1,j}[P2\cdot sR1]_{j} \\ -k_{on,M\cdot(P2\cdot sR1),j}[M_{EBM}]_{j}[P2\cdot sR1]_{j}+k_{off,M\cdot(P2\cdot sR1),j}[M_{EBM}\cdot P2\cdot sR1]_{j} \\ -k_{on,M\cdot(P2\cdot sR1),j}[M_{ECM}]_{j}[P2\cdot sR1]_{j}+k_{off,M\cdot(P2\cdot sR1),j}[M_{ECM}\cdot P2\cdot sR1]_{j} \\ -k_{on,M\cdot(P2\cdot sR1),j}[M_{PBM}]_{j}[P2\cdot sR1]_{j}+k_{off,M\cdot(P2\cdot sR1),j}[M_{PBM}\cdot P2\cdot sR1]_{j} \end{matrix}$$

## Blood Equations

The final set of 11 equations describes the binding and unbinding of molecular species in the blood, as well as clearance (k_CL_ in s^-1^, see **Table S10**). In this model, we assume no secretion of any molecular species directly into the blood.

$$\begin{matrix} d[V_{165}]_{B}/dt=-k_{CL,V165}+\frac{k_{L,N}}{U_{B}}\frac{[V_{165}]_{N}}{K_{Av,N}}+\frac{k_{L,D}}{U_{B}}\frac{[V_{165}]_{D}}{K_{Av,D}} \\ +\frac{\gamma_{N}\cdot S_{NB}}{U_{B}}\cdot\left(k_{p,V}^{N\to B}\frac{[V_{165}]_{N}}{K_{AV,N}}-k_{p,V}^{B\to N}\frac{[V_{165}]_{B}}{K_{AV,B}}\right) \\ +\frac{\gamma_{D}\cdot S_{DB}}{U_{B}}\cdot\left(k_{p,V}^{D\to B}\frac{[V_{165}]_{D}}{K_{AV,D}}-k_{p,V}^{B\to D}\frac{[V_{165}]_{B}}{K_{AV,B}}\right) \\ -k_{on,V165\cdot sR1}[V_{165}]_{B}[sR1]_{B}+k_{off,V165\cdot sR1,j}[V_{165}\cdot sR1]_{B} \end{matrix}$$

$$\begin{matrix} d[V_{189}]_{B}/dt=-k_{CL,V189}+\frac{k_{L,N}}{U_{B}}\frac{[V_{189}]_{N}}{K_{Av,N}}+\frac{k_{L,D}}{U_{B}}\frac{[V_{189}]_{D}}{K_{Av,D}} \\ +\frac{\gamma_{N}\cdot S_{NB}}{U_{B}}\cdot\left(k_{p,V}^{N\to B}\frac{[V_{189}]_{N}}{K_{AV,N}}-k_{p,V}^{B\to N}\frac{[V_{189}]_{B}}{K_{AV,B}}\right) \\ +\frac{\gamma_{D}\cdot S_{DB}}{U_{B}}\cdot\left(k_{p,V}^{D\to B}\frac{[V_{189}]_{D}}{K_{AV,D}}-k_{p,V}^{B\to D}\frac{[V_{189}]_{B}}{K_{AV,B}}\right) \\ -k_{on,V189\cdot sR1}[V_{189}]_{B}[sR1]_{B}+k_{off,V189\cdot sR1,j}[V_{189}\cdot sR1]_{B} \end{matrix}$$

$$\begin{matrix} d[V_{121}]_{B}/dt=-k_{CL,V121}+\frac{k_{L,N}}{U_{B}}\frac{[V_{121}]_{N}}{K_{Av,N}}+\frac{k_{L,D}}{U_{B}}\frac{[V_{121}]_{D}}{K_{Av,D}} \\ +\frac{\gamma_{N}\cdot S_{NB}}{U_{B}}\cdot\left(k_{p,V}^{N\to B}\frac{[V_{121}]_{N}}{K_{AV,N}}-k_{p,V}^{B\to N}\frac{[V_{121}]_{B}}{K_{AV,B}}\right) \\ +\frac{\gamma_{D}\cdot S_{DB}}{U_{B}}\cdot\left(k_{p,V}^{D\to B}\frac{[V_{121}]_{D}}{K_{AV,D}}-k_{p,V}^{B\to D}\frac{[V_{121}]_{B}}{K_{AV,B}}\right) \\ -k_{on,V121\cdot sR1}[V_{121}]_{B}[sR1]_{B}+k_{off,V121\cdot sR1,j}[V_{121}\cdot sR1]_{B} \end{matrix}$$

$$\begin{matrix} d[P1]_{B}/dt=-k_{CL,P1}+\frac{k_{L,N}}{U_{B}}\frac{[P1]_{N}}{K_{Av,N}}+\frac{k_{L,D}}{U_{B}}\frac{[P1]_{D}}{K_{Av,D}} \\ +\frac{\gamma_{N}\cdot S_{NB}}{U_{B}}\cdot\left(k_{p,P}^{N\to B}\frac{[P1]_{N}}{K_{AV,N}}-k_{p,P}^{B\to N}\frac{[P1]_{B}}{K_{AV,B}}\right) \\ +\frac{\gamma_{D}\cdot S_{DB}}{U_{B}}\cdot\left(k_{p,P}^{D\to B}\frac{[P1]_{D}}{K_{AV,D}}-k_{p,P}^{B\to D}\frac{[P1]_{B}}{K_{AV,B}}\right) \\ -k_{on,P1\cdot sR1}[P1]_{B}[sR1]_{B}+k_{off,P1\cdot sR1,j}[P1\cdot sR1]_{B} \end{matrix}$$

$$\begin{matrix} d[P2]_{B}/dt=-k_{CL,P2}+\frac{k_{L,N}}{U_{B}}\frac{[P2]_{N}}{K_{Av,N}}+\frac{k_{L,D}}{U_{B}}\frac{[P2]_{D}}{K_{Av,D}} \\ +\frac{\gamma_{N}\cdot S_{NB}}{U_{B}}\cdot\left(k_{p,P}^{N\to B}\frac{[P2]_{N}}{K_{AV,N}}-k_{p,P}^{B\to N}\frac{[P2]_{B}}{K_{AV,B}}\right) \\ +\frac{\gamma_{D}\cdot S_{DB}}{U_{B}}\cdot\left(k_{p,P}^{D\to B}\frac{[P2]_{D}}{K_{AV,D}}-k_{p,P}^{B\to D}\frac{[P2]_{B}}{K_{AV,B}}\right) \\ -k_{on,P2\cdot sR1}[P2]_{B}[sR1]_{B}+k_{off,P2\cdot sR1,j}[P2\cdot sR1]_{B} \end{matrix}$$

$$\begin{matrix} d[sR1]_{B}/dt=-k_{CL,sR1}+\frac{k_{L,N}}{U_{B}}\frac{[sR1]_{N}}{K_{Av,N}}+\frac{k_{L,D}}{U_{B}}\frac{[sR1]_{D}}{K_{Av,D}} \\ +\frac{\gamma_{N}\cdot S_{NB}}{U_{B}}\cdot\left(k_{p,sR1}^{N\to B}\frac{[sR1]_{N}}{K_{AV,N}}-k_{p,sR1}^{B\to N}\frac{[sR1]_{B}}{K_{AV,B}}\right) \\ +\frac{\gamma_{D}\cdot S_{DB}}{U_{B}}\cdot\left(k_{p,sR1}^{D\to B}\frac{[sR1]_{D}}{K_{AV,D}}-k_{p,sR1}^{B\to D}\frac{[sR1]_{B}}{K_{AV,B}}\right) \\ -k_{on,V165\cdot sR1}[V_{165}]_{B}[sR1]_{B}+k_{off,V165\cdot sR1,j}[V_{165}\cdot sR1]_{B} \\ -k_{on,V189\cdot sR1}[V_{189}]_{B}[sR1]_{B}+k_{off,V189\cdot sR1,j}[V_{189}\cdot sR1]_{B} \\ -k_{on,V121\cdot sR1}[V_{121}]_{B}[sR1]_{B}+k_{off,V121\cdot sR1,j}[V_{121}\cdot sR1]_{B} \\ -k_{on,P1\cdot sR1}[P1]_{B}[sR1]_{B}+k_{off,P1\cdot sR1,j}[P1\cdot sR1]_{B} \\ -k_{on,P2\cdot sR1}[P2]_{B}[sR1]_{B}+k_{off,P2\cdot sR1,j}[P2\cdot sR1]_{B} \end{matrix}$$

$$\begin{matrix} d[V_{165}\cdot sR1]_{B}/dt=-k_{CL,V165\cdot sR1}+\frac{k_{L,N}}{U_{B}}\frac{[V_{165}\cdot sR1]_{N}}{K_{Av,N}}+\frac{k_{L,D}}{U_{B}}\frac{[V_{165}\cdot sR1]_{D}}{K_{Av,D}} \\ +\frac{\gamma_{N}\cdot S_{NB}}{U_{B}}\cdot\left(k_{p,sR1}^{N\to B}\frac{[V_{165}\cdot sR1]_{N}}{K_{AV,N}}-k_{p,sR1}^{B\to N}\frac{[V_{165}\cdot sR1]_{B}}{K_{AV,B}}\right) \\ +\frac{\gamma_{D}\cdot S_{DB}}{U_{B}}\cdot\left(k_{p,sR1}^{D\to B}\frac{[V_{165}\cdot sR1]_{D}}{K_{AV,D}}-k_{p,sR1}^{B\to D}\frac{[V_{165}\cdot sR1]_{B}}{K_{AV,B}}\right) \\ +k_{on,V165\cdot sR1}[V_{165}]_{B}[sR1]_{B}-k_{off,V165\cdot sR1,j}[V_{165}\cdot sR1]_{B} \end{matrix}$$

$$\begin{matrix} d[V_{189}\cdot sR1]_{B}/dt=-k_{CL,V189\cdot sR1}+\frac{k_{L,N}}{U_{B}}\frac{[V_{189}\cdot sR1]_{N}}{K_{Av,N}}+\frac{k_{L,D}}{U_{B}}\frac{[V_{189}\cdot sR1]_{D}}{K_{Av,D}} \\ +\frac{\gamma_{N}\cdot S_{NB}}{U_{B}}\cdot\left(k_{p,sR1}^{N\to B}\frac{[V_{189}\cdot sR1]_{N}}{K_{AV,N}}-k_{p,sR1}^{B\to N}\frac{[V_{189}\cdot sR1]_{B}}{K_{AV,B}}\right) \\ +\frac{\gamma_{D}\cdot S_{DB}}{U_{B}}\cdot\left(k_{p,sR1}^{D\to B}\frac{[V_{189}\cdot sR1]_{D}}{K_{AV,D}}-k_{p,sR1}^{B\to D}\frac{[V_{189}\cdot sR1]_{B}}{K_{AV,B}}\right) \\ +k_{on,V189\cdot sR1}[V_{189}]_{B}[sR1]_{B}-k_{off,V189\cdot sR1,j}[V_{189}\cdot sR1]_{B} \end{matrix}$$

$$\begin{matrix} d[V_{121}\cdot sR1]_{B}/dt=-k_{CL,V121\cdot sR1}+\frac{k_{L,N}}{U_{B}}\frac{[V_{121}\cdot sR1]_{N}}{K_{Av,N}}+\frac{k_{L,D}}{U_{B}}\frac{[V_{121}\cdot sR1]_{D}}{K_{Av,D}} \\ +\frac{\gamma_{N}\cdot S_{NB}}{U_{B}}\cdot\left(k_{p,sR1}^{N\to B}\frac{[V_{121}\cdot sR1]_{N}}{K_{AV,N}}-k_{p,sR1}^{B\to N}\frac{[V_{121}\cdot sR1]_{B}}{K_{AV,B}}\right) \\ +\frac{\gamma_{D}\cdot S_{DB}}{U_{B}}\cdot\left(k_{p,sR1}^{D\to B}\frac{[V_{121}\cdot sR1]_{D}}{K_{AV,D}}-k_{p,sR1}^{B\to D}\frac{[V_{121}\cdot sR1]_{B}}{K_{AV,B}}\right) \\ +k_{on,V121\cdot sR1}[V_{121}]_{B}[sR1]_{B}-k_{off,V121\cdot sR1,j}[V_{121}\cdot sR1]_{B} \end{matrix}$$

$$\begin{matrix} d[P1\cdot sR1]_{B}/dt=-k_{CL,P1\cdot sR1}+\frac{k_{L,N}}{U_{B}}\frac{[P1\cdot sR1]_{N}}{K_{Av,N}}+\frac{k_{L,D}}{U_{B}}\frac{[P1\cdot sR1]_{D}}{K_{Av,D}} \\ +\frac{\gamma_{N}\cdot S_{NB}}{U_{B}}\cdot\left(k_{p,sR1}^{N\to B}\frac{[P1\cdot sR1]_{N}}{K_{AV,N}}-k_{p,sR1}^{B\to N}\frac{[P1\cdot sR1]_{B}}{K_{AV,B}}\right) \\ +\frac{\gamma_{D}\cdot S_{DB}}{U_{B}}\cdot\left(k_{p,sR1}^{D\to B}\frac{[P1\cdot sR1]_{D}}{K_{AV,D}}-k_{p,sR1}^{B\to D}\frac{[P1\cdot sR1]_{B}}{K_{AV,B}}\right) \\ +k_{on,P1\cdot sR1}[P1]_{B}[sR1]_{B}-k_{off,P1\cdot sR1,j}[P1\cdot sR1]_{B} \end{matrix}$$

$$\begin{matrix} d[P2\cdot sR1]_{B}/dt=-k_{CL,P2\cdot sR1}+\frac{k_{L,N}}{U_{B}}\frac{[P2\cdot sR1]_{N}}{K_{Av,N}}+\frac{k_{L,D}}{U_{B}}\frac{[P2\cdot sR1]_{D}}{K_{Av,D}} \\ +\frac{\gamma_{N}\cdot S_{NB}}{U_{B}}\cdot\left(k_{p,sR1}^{N\to B}\frac{[P2\cdot sR1]_{N}}{K_{AV,N}}-k_{p,sR1}^{B\to N}\frac{[P2\cdot sR1]_{B}}{K_{AV,B}}\right) \\ +\frac{\gamma_{D}\cdot S_{DB}}{U_{B}}\cdot\left(k_{p,sR1}^{D\to B}\frac{[P2\cdot sR1]_{D}}{K_{AV,D}}-k_{p,sR1}^{B\to D}\frac{[P2\cdot sR1]_{B}}{K_{AV,B}}\right) \\ +k_{on,P2\cdot sR1}[P2]_{B}[sR1]_{B}-k_{off,P2\cdot sR1,j}[P2\cdot sR1]_{B} \end{matrix}$$

**Supplemental References**

1. Stefanini MO, Wu FT, Mac Gabhann F, Popel AS. A compartment model of VEGF distribution in blood, healthy and diseased tissues. BMC Systems Biology. 2008;2. doi: 10.1186/1752-0509-2-77. PubMed PMID: WOS:000259952700001.

2. Wu FTH, Stefanini MO, Gabhann FM, Popel AS. A Compartment Model of VEGF Distribution in Humans in the Presence of Soluble VEGF Receptor-1 Acting as a Ligand Trap. Plos One. 2009;4(4). doi: 10.1371/journal.pone.0005108. PubMed PMID: WOS:000265505700013.
