# Supplementary figures and images for: A computational analysis of in vivo VEGFR activation by multiple co-expressed ligands
Source: PLoS Comput Biol. 2017 Mar 20;13(3):e1005445. doi: 10.1371/journal.pcbi.1005445 (PMC5378411; doi:10.1371/journal.pcbi.1005445)

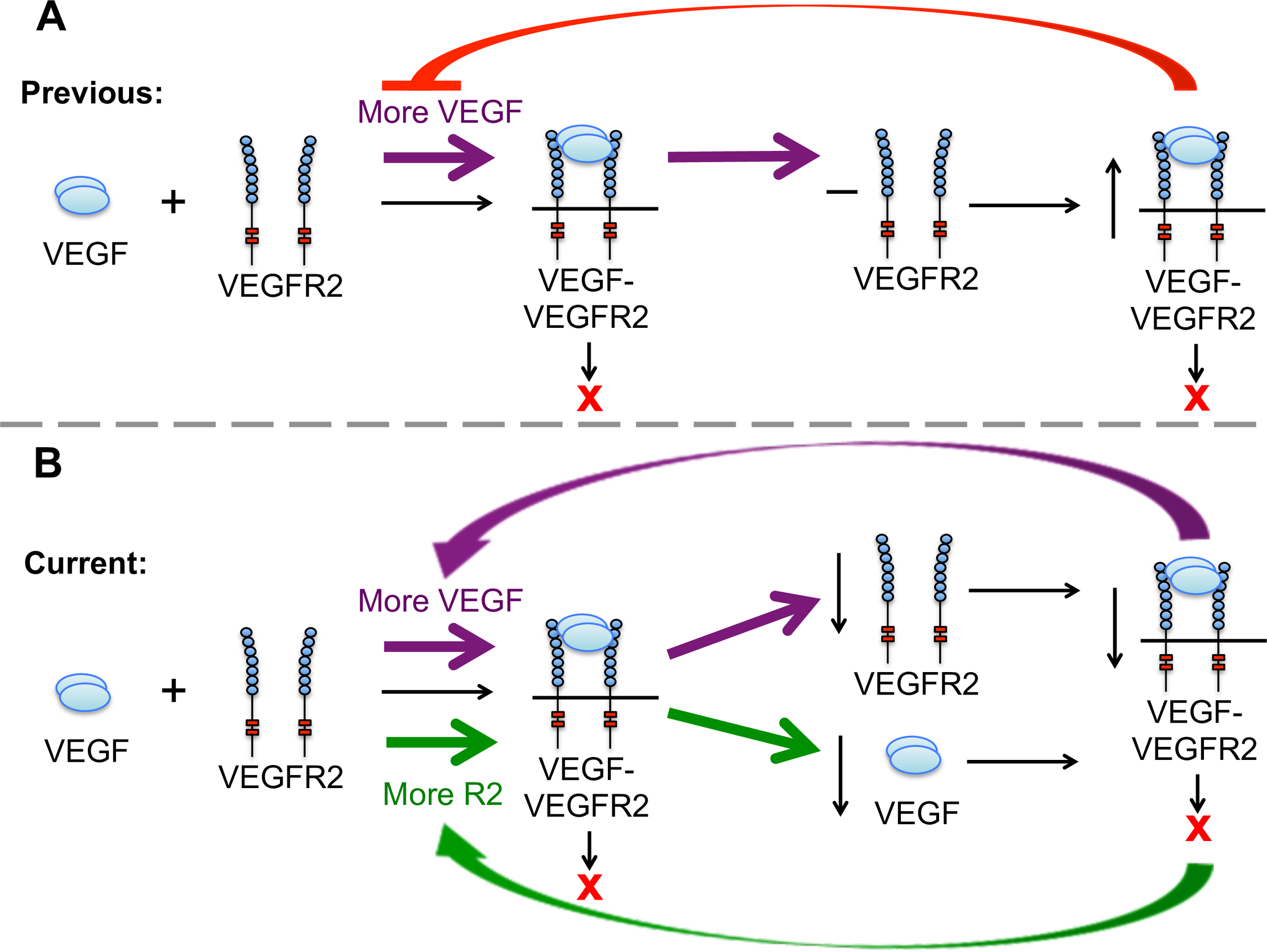

Supplement: S1 Fig — These panels expands upon the results shown in Fig 2 of the main manuscript. (A) In previous models, surface VEGFR2 levels were fixed (same internalization rate for free and VEGF-bound VEGFR2, no recycling), so increasing VEGF levels would lead to more VEGF-VEGFR2 binding and subsequent degradation of VEGF, keeping the net change in VEGF levels relatively small. (B) In this model, trafficking rates are different for free and ligand-bound VEGFR2, so endothelial cell surface VEGFR2 levels are not constant when VEGF levels change. If VEGF levels increase, more VEGFR2 becomes occupied, internalized, and degraded, reducing steady-state VEGFR2 levels and decreasing VEGF consumption via VEGFR2 (purple). Similarly, if VEGFR2 production increases, more VEGF is bound to VEGFR2, internalized, and degraded, reducing steady-state VEGF levels and as a result further increasing surface VEGFR2 (green). (TIF) [file pcbi.1005445.s003.tif]

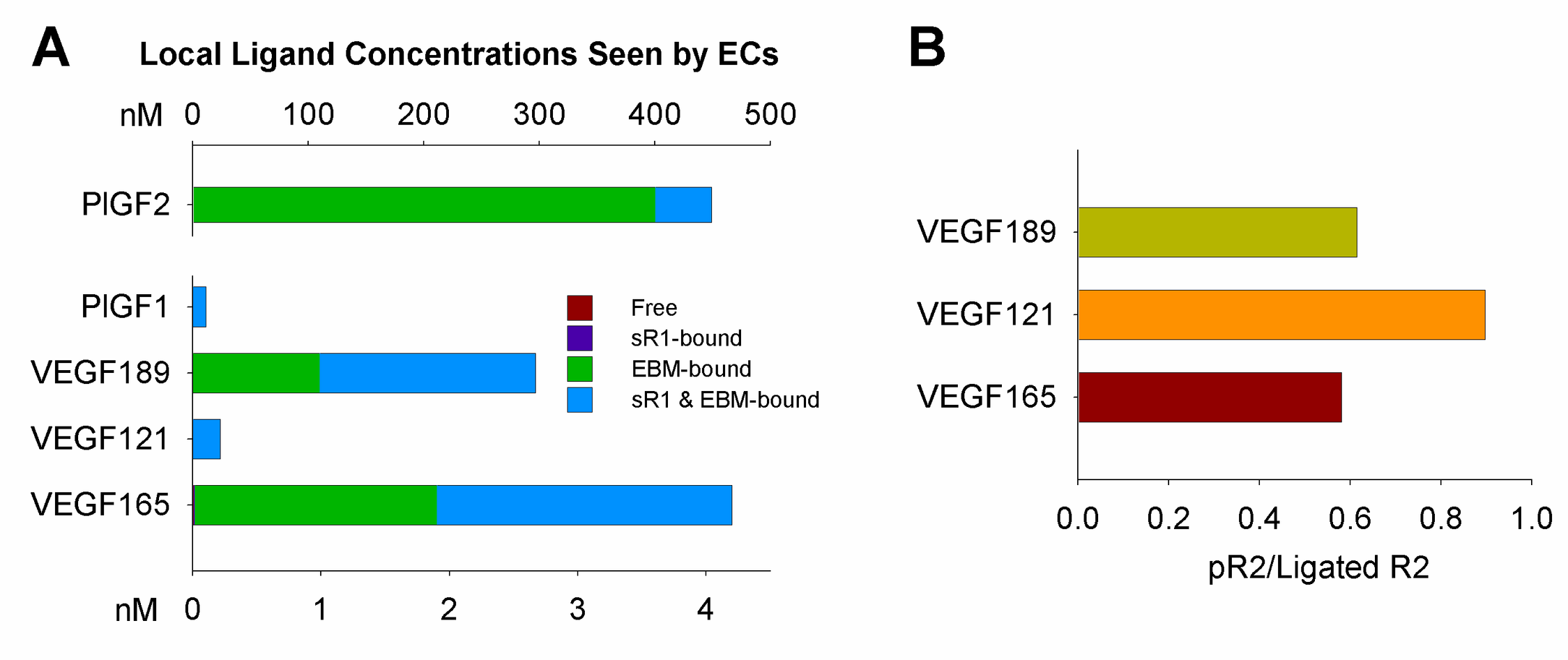

Supplement: S2 Fig — (A) This panel, which shows “local” concentrations of growth factor accessible to endothelial cell receptors, is related to Fig 4A of the main manuscript. EBM-bound growth factor concentrations are calculated using the EBM volume, while free levels are calculated using the total available interstitial space. (B) This panel expands upon the results shown in Fig 5 of the main manuscript. For each isoform, total phosphorylated VEGFR2 (pR2) bound to the given ligand is divided by total VEGFR2 bound to the respective ligand. (TIF) [file pcbi.1005445.s004.tif]

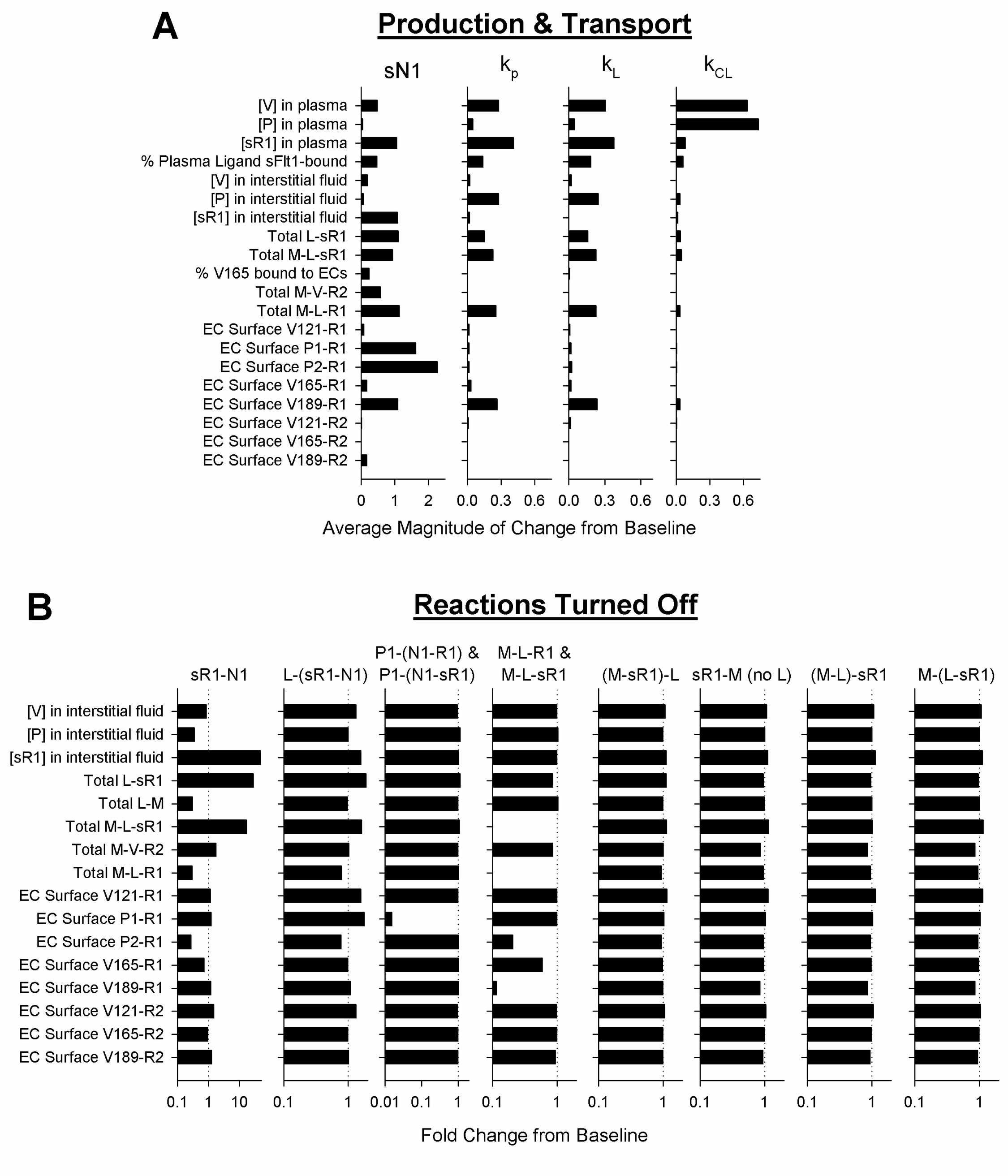

Supplement: S3 Fig — (A) Sensitivity of ligand distribution and receptor activation to changes in, from left to right: NRP1 production rate (sN1), vascular permeability (kp), lymphatic drainage rate (kL), and rate of clearance from the blood (kCL). All tissue quantities are taken from the “Main Body Mass” compartment. Values shown are the average magnitude of change in a given quantity when the specified parameter is increased or decreased by a factor of 10 (baseline = 0). Note the different scale on the NRP1 production rate than on the other panels. (B) Changes to ligand distribution and receptor activation when kon for different reactions is set to zero, prohibiting the selected reactions from occurring. Values shown are fold change from baseline (baseline = 1). Examined reactions are, from left to right: binding of sR1 to EC NRP1 (with or without ligand), binding of ligand to sR1-N1 complexes, binding of PlGF1 to NRP1-VEGFR1 and NRP1-sR1 complexes, formation of immobilized ligand-VEGFR1 and immobilized ligand-sR1 complexes (in any form), binding of VEGF121 or PlGF1 to immobilized sR1, binding of free sR1 to matrix proteins (no ligand), binding of immobilized ligands to sR1 (only), and binding of matrix proteins to VEGF165, VEGF189, or PlGF2 bound to sR1. All tissue quantities taken from “Main Body Mass” compartment. Note the different scale on the sR1-N1 and P1-(N1-R1) panels than on the other panels. (TIF) [file pcbi.1005445.s005.tif]
