# Supplementary material for: A computational analysis of in vivo VEGFR activation by multiple co-expressed ligands
Source: PLoS Comput Biol. 2017 Mar 20;13(3):e1005445. doi: 10.1371/journal.pcbi.1005445 (PMC5378411; doi:10.1371/journal.pcbi.1005445)
Supplement: S1 Table — (DOCX) [file pcbi.1005445.s006.docx]

**S1 Table. Binding/Unbinding Reactions: K_D_ in the main body mass**

| K_D_ | VEGF_165_ | VEGF_121_ | VEGF_189_ | PlGF1 | PlGF2 | Units | Refs fff Ref |
| --- | --- | --- | --- | --- | --- | --- | --- |
| L-R1 | 1.0 x 10^-15^ | 1.0 x 10^-15^ | 1.0 x 10^-15^ | 6.9 x 10^-15^ | 6.9 x 10^-15^ | moles/cm^3^ tissue | [1, 2] |
| L-R2 | 3.0 x 10^-15^ | 3.0 x 10^-15^ | 3.0 x 10^-15^ | - | - | moles/cm^3^ tissue | [1, 2] |
| L-N1 | 3.6 x 10^-14^ | - | 3.6 x 10^-15^ | - | 1.0 x 10^-7^ | moles/cm^3^ tissue | [3, 4] |
| L-sR1 | 1.0 x 10^-15^ | 1.0 x 10^-15^ | 1.0 x 10^-15^ | 6.9 x 10^-15^ | 6.9 x 10^-15^ | moles/cm^3^ tissue | [1] |
| L-M | 1.8 x 10^-12^ | - | 1.8 x 10^-13^ | - | 1.4 x 10^-13^ | moles/cm^3^ tissue | [5] |
| (M-L)-R1 | 1.0 x 10^-15^ | - | 1.0 x 10^-15^ | - | 6.9 x 10^-15^ | moles/cm^3^ tissue |  |
| (M-L)-R2 | 3.0 x 10^-15^ | - | 3.0 x 10^-15^ | - | - | moles/cm^3^ tissue |  |
| (M-L)-sR1 | 1.0 x 10^-15^ | - | 1.0 x 10^-15^ | - | 6.9 x 10^-15^ | moles/cm^3^ tissue |  |
| M-(L-R1) | 1.8 x 10^-12^ | - | 1.8 x 10^-13^ | - | 1.4 x 10^-13^ | moles/cm^3^ tissue |  |
| M-(L-R2) | 1.8 x 10^-12^ | - | 1.8 x 10^-13^ | - | - | moles/cm^3^ tissue |  |
| M-(L-sR1) | 1.8 x 10^-12^ | - | 1.8 x 10^-13^ | - | 1.4 x 10^-13^ | moles/cm^3^ tissue |  |
| (L-sR1)-M | - | 7.2 x 10^-13^ | - | 7.2 x 10^-13^ | - | moles/cm^3^ tissue |  |
| (M-sR1)-L | - | 6.9 x 10^-15^ | - | 6.9 x 10^-15^ | - | moles/cm^3^ tissue |  |
| (N1-L)-R2 | 7.3 x 10^-16^ | - | 7.3 x 10^-16^ | - | - | moles/cm^3^ tissue |  |
| N1-(L-R2) | 2.3 x 10^-15^ | - | 2.3 x 10^-15^ | - | - | moles/cm^3^ tissue |  |
| (L-R1)-N1 | - | 7.3 x 10^-15^ | - | 7.3 x 10^-15^ | - | moles/cm^3^ tissue |  |
| (L-sR1)-N1 | - | 5.4 x 10^-14^ | - | 5.4 x 10^-14^ | - | moles/cm^3^ tissue |  |
| (N1-R1)-L | - | 1.0 x 10^-15^ | - | 1.0 x 10^-15^ | - | moles/cm^3^ tissue |  |
| (N1-sR1)-L | - | 1.0 x 10^-15^ | - | 1.0 x 10^-15^ | - | moles/cm^3^ tissue |  |
|  |  |  |  |  |  |  |  |
| Other | N1-R1 | 7.3 x 10^-15^ | moles/cm^3^ tissue | |  |  | [1] |
|  | sR1-N1 | 5.4 x 10^-14^ | moles/cm^3^ tissue | |  |  | [3, 4] |
|  | sR1-M | 7.2 x 10^-13^ | moles/cm^3^ tissue | |  |  |  |

**Supplemental References**

1. Wu FTH, Stefanini MO, Gabhann FM, Popel AS. A Compartment Model of VEGF Distribution in Humans in the Presence of Soluble VEGF Receptor-1 Acting as a Ligand Trap. Plos One. 2009;4(4). doi: 10.1371/journal.pone.0005108. PubMed PMID: WOS:000265505700013.

2. Mac Gabhann F, Popel AS. Model of competitive binding of vascular endothelial growth factor and placental growth factor to VEGF receptors on endothelial cells. American Journal of Physiology-Heart and Circulatory Physiology. 2004;286(1). doi: 10.1152/ajpheart.00254.2003. PubMed PMID: WOS:000187350500021.

3. Vintonenko N, Pelaez-Garavito I, Buteau-Lozano H, Toullec A, Lidereau R, Perret GY, et al. Overexpression of VEGF189 in breast cancer cells induces apoptosis via NRP1 under stress conditions. Cell Adhesion & Migration. 2011;5(4):332-43. doi: 10.4161/cam.5.4.17287. PubMed PMID: WOS:000300713300008.

4. Hoffmann DC, Willenborg S, Koch M, Zwolanek D, Mueller S, Becker A-KA, et al. Proteolytic Processing Regulates Placental Growth Factor Activities. Journal of Biological Chemistry. 2013;288(25):17976-89. doi: 10.1074/jbc.M113.451831. PubMed PMID: WOS:000320721900005.

5. Martino MM, Briquez PS, Güç E, Tortelli F, Kilarski WW, Metzger S, et al. Growth Factors Engineered for Super-Affinity to the Extracellular Matrix Enhance Tissue Healing. Science. 2014;343(6173):885-8. doi: 10.1126/science.1247663.
