# Supplementary material for: A computational analysis of in vivo VEGFR activation by multiple co-expressed ligands
Source: PLoS Comput Biol. 2017 Mar 20;13(3):e1005445. doi: 10.1371/journal.pcbi.1005445 (PMC5378411; doi:10.1371/journal.pcbi.1005445)
Supplement: S3 Table — (DOCX) [file pcbi.1005445.s008.docx]

**S3 Table. Binding/Unbinding Reactions: K_D_ in plasma**

| K_D_ | VEGF_165_ | VEGF_121_ | VEGF_189_ | PlGF1 | PlGF2 | Units | RRefegs Ref |
| --- | --- | --- | --- | --- | --- | --- | --- |
| L-sR1 | 2.0 x 10^-14^ | 2.0 x 10^-14^ | 2.0 x 10^-14^ | 1.4 x 10^-13^ | 1.4 x 10^-13^ | moles/cm^3^ plasma | [1] |

**Supplemental References**

1. Wu FTH, Stefanini MO, Gabhann FM, Popel AS. A Compartment Model of VEGF Distribution in Humans in the Presence of Soluble VEGF Receptor-1 Acting as a Ligand Trap. Plos One. 2009;4(4). doi: 10.1371/journal.pone.0005108. PubMed PMID: WOS:000265505700013.
