# Supplementary material for: A computational analysis of in vivo VEGFR activation by multiple co-expressed ligands
Source: PLoS Comput Biol. 2017 Mar 20;13(3):e1005445. doi: 10.1371/journal.pcbi.1005445 (PMC5378411; doi:10.1371/journal.pcbi.1005445)
Supplement: S4 Table — (DOCX) [file pcbi.1005445.s009.docx]

**S4 Table. Binding/Unbinding Reactions: k_on_ in the main body mass**

| k_on_ | VEGF_165_ | VEGF_121_ | VEGF_189_ | PlGF1 | PlGF2 | Units |
| --- | --- | --- | --- | --- | --- | --- |
| L-R1 | 1.0 x 10^12^ | 1.0 x 10^12^ | 1.0 x 10^12^ | 5.0 x 10^10^ | 5.0 x 10^10^ | (moles/cm^3^ tissue)^-1^ s^-1^ |
| L-R2 | 3.3 x 10^11^ | 3.3 x 10^11^ | 3.3 x 10^11^ | - | - | (moles/cm^3^ tissue)^-1^ s^-1^ |
| L-N1 | 1.7 x 10^10^ | - | 4.7 x 10^10^ | - | 3.3 x 10^8^ | (moles/cm^3^ tissue)^-1^ s^-1^ |
| L-sR1 | 1.0 x 10^12^ | 1.0 x 10^12^ | 1.0 x 10^12^ | 5.0 x 10^10^ | 5.0 x 10^10^ | (moles/cm^3^ tissue)^-1^ s^-1^ |
| L-M | 5.3 x 10^9^ | - | 5.3 x 10^9^ | - | 7.3 x 10^10^ | (moles/cm^3^ tissue)^-1^ s^-1^ |
| (M-L)-R1 | 1.0 x 10^12^ | - | 1.0 x 10^12^ | - | 5.0 x 10^10^ | (moles/cm^3^ tissue)^-1^ s^-1^ |
| (M-L)-R2 | 3.3 x 10^11^ | - | 3.3 x 10^11^ | - | - | (moles/cm^3^ tissue)^-1^ s^-1^ |
| (M-L)-sR1 | 1.0 x 10^12^ | - | 1.0 x 10^12^ | - | 5.0 x 10^10^ | (moles/cm^3^ tissue)^-1^ s^-1^ |
| M-(L-R1) | 5.3 x 10^9^ | - | 5.3 x 10^9^ | - | 7.3 x 10^9^ | (moles/cm^3^ tissue)^-1^ s^-1^ |
| M-(L-R2) | 5.3 x 10^9^ | - | 5.3 x 10^9^ | - | - | (moles/cm^3^ tissue)^-1^ s^-1^ |
| M-(L-sR1) | 5.3 x 10^9^ | - | 5.3 x 10^9^ | - | 7.3 x 10^9^ | (moles/cm^3^ tissue)^-1^ s^-1^ |
| (L-sR1)-M | - | 1.4 x 10^10^ | - | 1.4 x 10^10^ | - | (moles/cm^3^ tissue)^-1^ s^-1^ |
| (M-sR1)-L | - | 1.0 x 10^12^ | - | 1.0 x 10^12^ | - | (moles/cm^3^ tissue)^-1^ s^-1^ |
| (N1-L)-R2 | 1.4 x 10^12^ | - | 1.4 x 10^12^ | - | - | (moles/cm^3^ tissue)^-1^ s^-1^ |
| N1-(L-R2) | 4.2 x 10^11^ | - | 4.2 x 10^11^ | - | - | (moles/cm^3^ tissue)^-1^ s^-1^ |
| (L-R1)-N1 | - | 1.4 x 10^12^ | - | 1.4 x 10^12^ | - | (moles/cm^3^ tissue)^-1^ s^-1^ |
| (L-sR1)-N1 | - | 1.9 x 10^11^ | - | 1.9 x 10^11^ | - | (moles/cm^3^ tissue)^-1^ s^-1^ |
| (N1-R1)-L | - | 1.0 x 10^12^ | - | 5.0 x 10^10^ | - | (moles/cm^3^ tissue)^-1^ s^-1^ |
| (N1-sR1)-L | - | 1.0 x 10^12^ | - | 5.0 x 10^10^ | - | (moles/cm^3^ tissue)^-1^ s^-1^ |
|  |  |  |  |  |  |  |
| Other | N1-R1 | 1.4 x 10^12^ | (moles/cm^3^ tissue)^-1^ s^-1^ | |  |  |
|  | sR1-N1 | 1.9 x 10^11^ | (moles/cm^3^ tissue)^-1^ s^-1^ | |  |  |
|  | sR1-M | 1.4 x 10^11^ | (moles/cm^3^ tissue)^-1^ s^-1^ | |  |  |
