# Supplementary material for: A computational analysis of in vivo VEGFR activation by multiple co-expressed ligands
Source: PLoS Comput Biol. 2017 Mar 20;13(3):e1005445. doi: 10.1371/journal.pcbi.1005445 (PMC5378411; doi:10.1371/journal.pcbi.1005445)
Supplement: S6 Table — (DOCX) [file pcbi.1005445.s011.docx]

**S6 Table. Binding/Unbinding Reactions: k_on_ in plasma**

| k_on_ | VEGF_165_ | VEGF_121_ | VEGF_189_ | PlGF1 | PlGF2 | Units |
| --- | --- | --- | --- | --- | --- | --- |
| L-sR1 | 3.3 x 10^10^ | 3.3 x 10^10^ | 3.3 x 10^10^ | 2.5 x 10^9^ | 2.5 x 10^9^ | (moles/cm^3^ tissue)^-1^ s^-1^ |
