# Supplementary material for: A computational analysis of in vivo VEGFR activation by multiple co-expressed ligands
Source: PLoS Comput Biol. 2017 Mar 20;13(3):e1005445. doi: 10.1371/journal.pcbi.1005445 (PMC5378411; doi:10.1371/journal.pcbi.1005445)
Supplement: S7 Table — (DOCX) [file pcbi.1005445.s012.docx]

**S7 Table. Geometric Parameterization** [1]

|  | Main Body Mass Value | Calf Muscle Value | Units |
| --- | --- | --- | --- |
| Compartment Volume | 60,453 | 868 | cm^3^ |
| Individual Muscle Fiber |  |  |  |
| Diameter | 71 | 73 | μm |
| Perimeter correction factor | 1.14 | 1.14 |  |
| Perimeter | 253 | 261 | μm |
| FCSA | 3904 | 4173 | μm^2^ |
| Myonuclear density | 120 | 150 | mm^-1^ |
| MDSA | 2104 | 1740 | μm^2^/MD |
| Muscle Fiber Space |  |  |  |
| Muscle fiber density | 242 | 199 | fibers/mm^2^  tissue |
| FSAV | 611 | 520 | cm^2^/cm^3^ tissue |
| Muscle fiber space volume fraction | 94.4% | 83.1% | cm^3^/cm^3^ tissue |
| Individual Capillary |  |  |  |
| Luminal diameter | 4.86 | 3.97 | μm |
| Endothelium thickness | 0.77 | 0.78 | μm |
| Abluminal diameter | 6.39 | 5.53 | μm |
| Perimeter correction factor | 1.1 | 1.1 |  |
| Abluminal perimeter | 22.1 | 19.1 | μm |
| CCSA | 32.1 | 24.0 | μm^2^ |
| Lumen CSA | 18.6 | 12.4 | μm^2^ |
| Endothelium CSA | 13.5 | 11.6 | μm^2^ |
| ECSA (abluminal) | 1000 | 1000 | μm^2^/EC |
| Capillary Space |  |  |  |
| Capillary:fiber ratio | 1.36 | 1.16 |  |
| Capillary density | 329 | 231 | capillaries/mm^2^ tissue |
| ESAV (abluminal) | 73 | 44 | cm^2^/cm^3^ tissue |
| Capillary space volume fraction | 1.1% | 0.6% | cm^3^/cm^3^ tissue |
| Endothelium space | 0.4% | 0.3% | cm^3^/cm^3^ tissue |
| Vascular space | 0.6% | 0.3% | cm^3^/cm^3^ tissue |
| Interstitial Space |  |  |  |
| IS volume fraction | 4.5% | 16.3% | cm^3^/cm^3^ tissue |
| IF volume fraction | 3.7% | 13.7% | cm^3^/cm^3^ tissue |
| Available IF volume fraction | 3% | 11% | cm^3^/cm^3^ tissue |
| Extracellular Matrix (ECM) |  |  |  |
| ECM volume | 3.9% | 14.9% | cm^3^/cm^3^ tissue |
|  | 86.72% | 91.24% | cm^3^/cm^3^ IS |
| Solid fraction | 13.40% | 13.40% | cm^3^/cm^3^ ECM |
| Fluid volume in ECM | 3.38% | 12.92% | cm^3^/cm^3^ tissue |
|  | 91.13% | 94.25% | cm^3^/cm^3^ IF |
| Available fluid volume in ECM | 2.87% | 10.98% | cm^3^/cm^3^ tissue |
| Endothelial Basement Membrane (EBM) |  |  |  |
| Thickness | 87.5 | 254 | nm |
| EBM volume | 0.06% | 0.11% | cm^3^/cm^3^ tissue |
|  | 1.41% | 0.69% | cm^3^/cm^3^ IS |
| Solid fraction | 45% | 45% | cm^3^/cm^3^ BME |
| Fluid volume in EBM | 0.03% | 0.06% | cm^3^/cm^3^ tissue |
|  | 0.94% | 0.45% | cm^3^/cm^3^ IF |
| Available fluid volume in EBM | 0.01% | 0.02% | cm^3^/cm^3^ tissue |
| EBM Thickness accessible to EC receptors* | **25** | **25** | **nm** |
| Fraction EBM accessible to EC receptors | **28.6%** | **9.84%** | **cm^3^/cm^3^ BME** |
| Parenchymal Basement Membrane (PBM) |  |  |  |
| Thickness | 87.5 | 254 | nm |
| PBM volume | 0.53% | 1.32% | cm^3^/cm^3^ tissue |
|  | 11.87% | 8.07% | cm^3^/cm^3^ IS |
| Solid fraction | 45% | 45% | cm^3^/cm^3^ BMP |
| Fluid volume in PBM | 0.29% | 0.73% | cm^3^/cm^3^ tissue |
|  | 7.92% | 5.30% | cm^3^/cm^3^ IF |
| Available fluid volume in PBM | 0.10% | 0.24% | cm^3^/cm^3^ tissue |
| Blood Compartment |  |  |  |
| Total Volume | 5 |  | L |
| Plasma Fraction | 60% |  | cm^3^/cm^3^ blood |

*Based on length of ErbB2 and ErbB3 extracellular domains (11.3-16.4nm [2-4]), assuming some flexibility in cell shape and position.

**Bold:** new parameters

**Supplemental References**

1. Wu FTH, Stefanini MO, Gabhann FM, Popel AS. A Compartment Model of VEGF Distribution in Humans in the Presence of Soluble VEGF Receptor-1 Acting as a Ligand Trap. Plos One. 2009;4(4). doi: 10.1371/journal.pone.0005108. PubMed PMID: WOS:000265505700013.

2. Hu S, Sun Y, Meng Y, Wang X, Yang W, Fu W, et al. Molecular architecture of the ErbB2 extracellular domain homodimer. Oncotarget. 2015;6(3):1695-706. PubMed PMID: WOS:000352689800031.

3. Cho HS, Leahy DJ. Structure of the extracellular region of HER3 reveals an interdomain tether. Science. 2002;297(5585):1330-3. doi: 10.1126/science.1074611. PubMed PMID: WOS:000177573900042.

4. Cho HS, Mason K, Ramyar KX, Stanley AM, Gabelli SB, Denney DW, et al. Structure of the extracellular region of HER2 alone and in complex with the Herceptin Fab. Nature. 2003;421(6924):756-60. doi: 10.1038/nature01392. PubMed PMID: WOS:000180938000047.
