# Supplementary material for: A computational analysis of in vivo VEGFR activation by multiple co-expressed ligands
Source: PLoS Comput Biol. 2017 Mar 20;13(3):e1005445. doi: 10.1371/journal.pcbi.1005445 (PMC5378411; doi:10.1371/journal.pcbi.1005445)
Supplement: S8 Table — (DOCX) [file pcbi.1005445.s013.docx]

**S8 Table. Trafficking Parameters** [1]

| Species Units: s^-1^ | k_int_ (from surface) | k_rec4_  (from Rab4/5) | k_rec11_ (from Rab11) | k_4to11_  (from Rab4/5) | k_degr_  (from Rab4/5) |
| --- | --- | --- | --- | --- | --- |
| R2 | 2.6 x 10^-3^ | 3.8 x 10^-3^ | 1.4 x 10^-4^ | 1.0 x 10^-5^ | 8.6 x 10^-6^ |
| V·R2 | 3.12 x 10^-2^ | 3.8 x 10^-3^ | 1.4 x 10^-4^ | 1.0 x 10^-5^ | 8.6 x 10^-5^ |
| M$\boldsymbol{\cdot}$V$\boldsymbol{\cdot}$R2 | 0 | - | - | - | - |
| L | 0 | 0 | 0 | 0 | 1.2 x 10^-2^ |
| N1 | 2.6 x 10^-3^ | 3.8 x 10^-5^ | 1.4 x 10^-2^ | 1.9 x 10^-2^ | 3.8 x 10^-4^ |
| V·N1 | 2.6 x 10^-3^ | 3.8 x 10^-5^ | 1.4 x 10^-2^ | 1.9 x 10^-2^ | 3.8 x 10^-4^ |
| V·N1·R2 | 3.12 x 10^-2^ | 3.8 x 10^-5^ | 1.4 x 10^-2^ | 1.9 x 10^-2^ | 6.8 x 10^-4^ |
| R1 | 2.6 x 10^-3^ | 3.8 x 10^-3^ | 1.4 x 10^-4^ | 1.0 x 10^-5^ | 8.6 x 10^-5^ |
| N1·R1 | 2.6 x 10^-3^ | 3.8 x 10^-5^ | 1.4 x 10^-2^ | 1.9 x 10^-2^ | 3.8 x 10^-4^ |
| L·R1 | 3.12 x 10^-2^ | 3.8 x 10^-3^ | 1.4 x 10^-4^ | 1.0 x 10^-5^ | 8.6 x 10^-5^ |
| L·N1·R1 | 3.12 x 10^-2^ | 3.8 x 10^-5^ | 1.4 x 10^-2^ | 1.9 x 10^-2^ | 3.8 x 10^-4^ |
| M$\boldsymbol{\cdot}$V$\boldsymbol{\cdot}$R1 | 0 | - | - | - | - |
| L·sR1 | 0 | 0 | 0 | 0 | 1.2 x 10^-2^ |
| sR1·N1 | 2.6 x 10^-3^ | 3.8 x 10^-5^ | 1.4 x 10^-2^ | 1.9 x 10^-2^ | 3.8 x 10^-4^ |
| L·sR1·N1 | 2.6 x 10^-3^ | 3.8 x 10^-5^ | 1.4 x 10^-2^ | 1.9 x 10^-2^ | 3.8 x 10^-4^ |

L: ligand- all trafficking parameters independent of ligand identify. Free sR1 trafficked with same rates as L·sR1.

**Supplemental References**

1. Clegg LW, Mac Gabhann F. Site-Specific Phosphorylation of VEGFR2 Is Mediated by Receptor Trafficking: Insights from a Computational Model. PLoS Comput Biol. 2015;11(6):e1004158. doi: 10.1371/journal.pcbi.1004158.
