# Supplementary material for: A computational analysis of in vivo VEGFR activation by multiple co-expressed ligands
Source: PLoS Comput Biol. 2017 Mar 20;13(3):e1005445. doi: 10.1371/journal.pcbi.1005445 (PMC5378411; doi:10.1371/journal.pcbi.1005445)
Supplement: S9 Table — (DOCX) [file pcbi.1005445.s014.docx]

**S9 Table. Phosphorylation Parameters** [1]

|  | Free R2 | | | V-R2 (not affected by N1 or M) | | |
| --- | --- | --- | --- | --- | --- | --- |
|  | Cell Surface | Rab4/5 Endosomes | Rab11 Endosomes | Cell Surface | Rab4/5 Endosomes | Rab11 Endosomes |
| k_p_ (s^-1^) | 0 | 0 | 0 | 1 | 1 | 1 |
| k_dp,Y951_ (s^-1^) | 30 | 30 | 30 | 0.043 | 75 | 30 |
| k_dp,Y1775_ (s^-1^) | 30 | 30 | 30 | 4.98 | 0.00972 | 30 |
| k_dp,Y1214_ (s^-1^) | 30 | 30 | 30 | 1.06 | 0.0307 | 30 |

Notes:

k_p_: phosphorylation rate constant for all tyrosine sites

k_dp_: site-specific dephosphorylation rate constant

**Supplemental References**

1. Clegg LW, Mac Gabhann F. Site-Specific Phosphorylation of VEGFR2 Is Mediated by Receptor Trafficking: Insights from a Computational Model. PLoS Comput Biol. 2015;11(6):e1004158. doi: 10.1371/journal.pcbi.1004158.
