# Supplementary material for: A computational analysis of in vivo VEGFR activation by multiple co-expressed ligands
Source: PLoS Comput Biol. 2017 Mar 20;13(3):e1005445. doi: 10.1371/journal.pcbi.1005445 (PMC5378411; doi:10.1371/journal.pcbi.1005445)
Supplement: S10 Table — (DOCX) [file pcbi.1005445.s015.docx]

**S10 Table. Transport Parameters** [1]

|  | Permeability  (bi-directional) | Lymphatic Drainage  (main body mass to blood) | Lymphatic Drainage (calf muscle to blood) | Clearance from Blood |
| --- | --- | --- | --- | --- |
| VEGF | 4.39 x 10^-8^ cm/s | 0.1418 cm^3^/s | 0.0026 cm^3^/s | 1.08 x 10^-3^ s^-1^ |
| PlGF | 4.39 x 10^-8^ cm/s | 0.1418 cm^3^/s | 0.0026 cm^3^/s | 1.08 x 10^-3^ s^-1^ |
| sFlt1 | 1.86 x 10^-8^ cm/s | 0.1418 cm^3^/s | 0.0026 cm^3^/s | 5.0 x 10^-6^ s^-1^ |
| VEGF-sFlt1 | 1.86 x 10^-8^ cm/s | 0.1418 cm^3^/s | 0.0026 cm^3^/s | 5.0 x 10^-6^ s^-1^ |
| PlGF-sFlt1 | 1.86 x 10^-8^ cm/s | 0.1418 cm^3^/s | 0.0026 cm^3^/s | 5.0 x 10^-6^ s^-1^ |

Note: Permeability rates apply to both calf muscle & main body mass. Geometric unit conversions applied [1].

**Supplemental References**

1. Wu FTH, Stefanini MO, Gabhann FM, Popel AS. A Compartment Model of VEGF Distribution in Humans in the Presence of Soluble VEGF Receptor-1 Acting as a Ligand Trap. Plos One. 2009;4(4). doi: 10.1371/journal.pone.0005108. PubMed PMID: WOS:000265505700013.
