# Supplementary material for: A computational analysis of in vivo VEGFR activation by multiple co-expressed ligands
Source: PLoS Comput Biol. 2017 Mar 20;13(3):e1005445. doi: 10.1371/journal.pcbi.1005445 (PMC5378411; doi:10.1371/journal.pcbi.1005445)
Supplement: S11 Table — (DOCX) [file pcbi.1005445.s016.docx]

**S11 Table. Available Matrix Site Densities** [1]

|  | ECM | EBM | PBM |
| --- | --- | --- | --- |
| Value (μM) | 0.75 | 20 | 20 |
| Main Body Mass (moles/cm^3^ tissue) | 2.15 x 10^-11^ | 2.0 x 10^-12^ | 2.0 x 10^-11^ |
| Calf Muscle (moles/cm^3^ tissue) | 8.24 x 10^-11^ | 4.0 x 10^-12^ | 4.8 x 10^-11^ |

Note: Unit conversions described in [1, 2].

**Supplemental References**

1. Wu FTH, Stefanini MO, Gabhann FM, Popel AS. A Compartment Model of VEGF Distribution in Humans in the Presence of Soluble VEGF Receptor-1 Acting as a Ligand Trap. Plos One. 2009;4(4). doi: 10.1371/journal.pone.0005108. PubMed PMID: WOS:000265505700013.

2. Wu FT, Stefanini MO, Mac Gabhann F, Kontos CD, Annex BH, Popel AS. VEGF and soluble VEGF receptor-1 (sFlt-1) distributions in peripheral arterial disease: an in silico model. Am J Physiol Heart Circ Physiol. 2010;298(6):H2174-91. Epub 2010/04/13. doi: ajpheart.00365.2009 [pii]

10.1152/ajpheart.00365.2009. PubMed PMID: 20382861; PubMed Central PMCID: PMC2886617.
