# Supplementary material for: A computational analysis of in vivo VEGFR activation by multiple co-expressed ligands
Source: PLoS Comput Biol. 2017 Mar 20;13(3):e1005445. doi: 10.1371/journal.pcbi.1005445 (PMC5378411; doi:10.1371/journal.pcbi.1005445)
Supplement: S12 Table — (DOCX) [file pcbi.1005445.s017.docx]

**S12 Table. Production and secretion rates for “MLR” cases** (**Figure 7**)

| Species | Target Location | Baseline | Cell Only | sR1 Only | No MLR | Production Units |
| --- | --- | --- | --- | --- | --- | --- |
| VEGFR1 | Main Body Mass | 1.162 | 1.20 | 1.14 | 1.175 | Change from No VEGF SS |
|  | Calf | 1.32 | 1.42 | 1.42 | 1.38 | Change from No VEGF SS |
| VEGFR2 | Main Body Mass | 32.09 | 31.10 | 29.12 | 28.763 | Change from No VEGF SS |
|  | Calf | 53.96 | 52.27 | 48.9 | 48.295 | Change from No VEGF SS |
| NRP1 | Main Body Mass | 1.295 | 1.285 | 1.267 | 1.262 | Change from No VEGF SS |
|  | Calf | 1.502 | 1.482 | 1.455 | 1.445 | Change from No VEGF SS |
| sR1 | Plasma | 0.0893 | 0.0838 | 0.0856 | 0.0813 | molec/EC/s |
| PlGF | Plasma | 0.0146 | 0.0144 | 0.0142 | 0.0142 | molec/MD/s |
| VEGF | Plasma | 0.2830 | 0.2733 | 0.2560 | 0.2522 | molec/MD/s |

*Note: Both M-L-sR1 and L-sR1-M complexes are included/excluded in “sR1” reactions.

SS: steady-state
