# Supplementary material for: A computational analysis of in vivo VEGFR activation by multiple co-expressed ligands
Source: PLoS Comput Biol. 2017 Mar 20;13(3):e1005445. doi: 10.1371/journal.pcbi.1005445 (PMC5378411; doi:10.1371/journal.pcbi.1005445)
Supplement: S13 Table — (DOCX) [file pcbi.1005445.s018.docx]

**S13 Table. Production and secretion rates for Single VEGF Isoform cases**
(**Figure 8**)

| Species | Target Location | Baseline | VEGF_121_ | VEGF_165_ | VEGF_189_ | Production Units |
| --- | --- | --- | --- | --- | --- | --- |
| VEGFR1 | Main Body Mass | 1.162 | 3.40 | 0.93 | 0.93 | Change from No VEGF SS |
|  | Calf | 1.32 | 5.51 | 0.932 | 0.935 | Change from No VEGF SS |
| VEGFR2 | Main Body Mass | 32.09 | 14.3 | 31.465 | 52.91 | Change from No VEGF SS |
|  | Calf | 53.96 | 30.2 | 52.81 | 89.42 | Change from No VEGF SS |
| NRP1 | Main Body Mass | 1.295 | 1.195 | 1.285 | 1.485 | Change from No VEGF SS |
|  | Calf | 1.502 | 1.27 | 1.482 | 1.823 | Change from No VEGF SS |
| sR1 | Plasma | 0.0893 | 0.5564 | 0.0588 | 0.0588 | molec/EC/s |
| PlGF | Plasma | 0.0146 | 0.0144 | 0.0146 | 0.0146 | molec/MD/s |
| VEGF | Plasma | 0.2830 | 0.3080 | 0.2637 | 0.4485 | molec/MD/s |

SS: steady-state
